# Reduced Expression of Selected Exosomal MicroRNAs Is Associated with Poor Outcomes in Patients with Acute Stroke Receiving Reperfusion Therapy—Preliminary Study

**DOI:** 10.3390/ijms26199533

**Published:** 2025-09-29

**Authors:** Daria Gendosz de Carrillo, Olga Kocikowska, Aleksandra Krzan, Sebastian Student, Małgorzata Rak, Magdalena Nowak-Andraka, Junqiao Mi, Małgorzata Burek, Anetta Lasek-Bal, Halina Jędrzejowska-Szypułka

**Affiliations:** 1 Department of Physiology, Faculty of Medicine, Medical University of Silesia in Katowice, 40-752 Katowice, Poland; olga.kocikowska@sum.edu.pl (O.K.); malgorzata.rak@sum.edu.pl (M.R.); s83314@365.sum.edu.pl (M.N.-A.); hszypulka@sum.edu.pl (H.J.-S.); 2 Department of Histology and Cell Pathology, Faculty of Medical Sciences in Zabrze, Medical University of Silesia in Katowice, 40-752 Katowice, Poland; 3 Department of Engineering and Systems Biology, Faculty of Automatic Control, Electronics and Computer Science, Silesian University of Technology, 44-100 Gliwice, Poland; sebastian.student@polsl.pl; 4 Department of Neurology, School of Health Sciences, Medical University of Silesia in Katowice, 40-752 Katowice, Poland; aleksandra.krzan@sum.edu.pl (A.K.); alasek@gcm.pl (A.L.-B.); 5 Department of Neurology, Upper-Silesian Medical Center of the Silesian Medical University, 40-752 Katowice, Poland; 6 Biotechnology Centre, Silesian University of Technology, 44-100 Gliwice, Poland; 7 Department of Anaesthesiology, Intensive Care, Emergency and Pain Medicine, University Hospital Wuerzburg, 97080 Wuerzburg, Germany; mi_j@ukw.de (J.M.); burek_m@ukw.de (M.B.)

**Keywords:** stroke, reperfusion treatment, MT, rt-PA, exosomal miRNA, mRS, enrichment analysis

## Abstract

Reperfusion therapy uses thrombolysis and clot removal to restore blood flow in the brain after stroke; however, three months after reperfusion therapy, roughly 46% of stroke patients become independent again. MiRNAs (micro RNA) regulate cerebral ischemia/reperfusion injury, and their transfer between cells via exosomes may differentially affect recipient cells. We examined serum exosomal miRNA levels, stroke treatments, and functional outcomes in stroke patients, and we explored the potential role of estimated differentially expressed miRNA (DEmiRNA) target genes in the brain’s reaction to reperfusion after ischemia. The patients in the study received aspirin or reperfusion therapy with either intravenous thrombolysis (rt-PA), mechanical thrombectomy (MT), or a combination of both (rt-PA/MT). Serum samples were collected from stroke patients on days 1 and 10 post-stroke. Serum exosomes’ miRNA was analyzed using qRT-PCR. We identified DEmiRNAs, estimated their targets, and performed enrichment analysis. Functional outcomes were assessed using the modified Rankin Scale (mRS) on days 10 and 90 post-stroke. Among studied treatments, only rt-PA/MT lowered DEmiRNA by day 10 vs. other groups. Specifically, patients with unfavorable mRS score exhibited decreased levels of miR-17, miR-20, miR-186 and miR-222 after combined stroke therapy. Functional analysis identified target genes and pathways associated with cytoskeleton remodeling, cell death, autophagy, inflammation, and dementia. In conclusion, unfavorable stroke outcomes following poor rt-PA/MT response could result from lower miRNA expression levels, thus activating cell death and neurodegenerative processes in brain.

## 1. Introduction

Stroke is one of the leading causes of death and long-term disabilities in adults worldwide. The Global Burden of Diseases report states that the number of strokes increased worldwide between 1990 and 2019, particularly in the population of patients under the age of 65 [1]. The modern reperfusion treatment (RT) of ischemic stroke allows for lysis and/or the removal of thrombus from the closed lumen of the carotid or cerebral arteries via intravenous thrombolysis with RT-plasminogen activator (rt-PA), and/or mechanical thrombectomy (MT). These methods increase the likelihood of penumbra reperfusion, which occurs in the ischemic region around the ischemic core. RT (i.e., intravenous rt-PA and/or MT) aims to preserve as many hypoxic cells as possible in this zone, including neuronal, endothelial, and glial cells.

Intravenous thrombolysis (rt-PA) has been the standard treatment for stroke since 1995. The administration time, initially 3 h post-disease onset, has increased to 4.5 h [2]. However, in about half of the patients, rt-PA may not adequately restore blood flow, or may increase the risk of bleeding in the brain [3]. Introduction of MT in 2016 was a breakthrough in the treatment of acute ischemic stroke caused by large-vessel occlusion (LVO). Based on the results of the meta-analysis, the use of MT in stroke patients results in the recanalization of over 80% of the arteries that underwent the intervention and a return to full patient independence in approximately 45% of patients within 3 months after stroke. However, according to international stroke registries, mechanical reperfusion has been unsuccessful in up to 30% of treated patients, with potentially detrimental consequences for functional outcomes [2,3,4]. This reveals that a significant proportion of patients have futile recanalization (as their angiogram showed recanalization). To date, evidence of the efficacy and safety of rt-PA preceding MT is scarce. There has been a debate whether MT alone is as effective as the combined treatment of rt-PA with MT regarding clinical outcomes of LVO-stroke patients. Some authors suggest that a combined treatment is associated with higher improvement in the functional outcomes of patients at 90 days post-stroke compared with MT alone, while others did not report any additional clinical benefit of a combined treatment [5,6,7]. We expect the novel thrombolytic agent tenecteplase to enhance the clinical effectiveness of combined reperfusion therapy. There are likely various factors that influence the clinical outcome of MT. The unfavorable prognostic parameters identified in the subpopulation of endovascularly treated stroke patients include older age, diabetes mellitus, severe neurological deficit on the first day of stroke, and a low ASPECTS scale score [8,9,10,11]. Two recent studies (SWIFT DIRECT and DIRECT-SAFE) did not show noninferiority of MT in the comparison to rt-PA with MT in terms of functional outcomes [12,13]. Recent studies and meta-analyses have highlighted MT as an important treatment modality for stroke because of its potential to increase the length of the therapeutic window [14,15]. According to the latest guidelines, the MT can be performed up to 24 h after stroke onset in precisely selected patients, based on radiological and clinical findings [16]. Regardless, up to 9% of patients reocclude within 48 h after complete recanalization. Administration of antiplatelet therapy (aspirin, clopidogrel) is recommended in patients with AIS within 24 to 48 h after the onset. For those treated with reperfusion therapy (MT, rt-PA), antiplatelet treatment is started after a CT of the head performed 24 h after procedure. For patients with double occlusion disease (severe carotid stenosis and MCA occlusion) undergoing carotid angioplasty with stent placement, and potentially additional MT, antiplatelet therapy begins up to 12 h post-procedure. For vulnerable patients, such as those with atrial fibrillation, anticoagulants are used in the acute stroke phase following a brief period of antiplatelet therapy.

In the age of emerging personalized medicine, biochemical and molecular biomarkers are being investigated to improve the prognostic value regarding the course of stroke and post-stroke disability. Exosomes are the smallest subtype of extracellular vesicles and are secreted by almost all eukaryotic cells. The vesicles are between 50 and 150 nm in size, are surrounded by a lipid bilayer, and are estimated to contain 0.0001% of the cellular volume [17], in which we may find various soluble substances such as lipids, protein, DNA, and RNA, including miRNA [18]. Exosomes released from cells can transfer miRNAs between cells and tissues, significantly impacting recipient cell physiology and gene expression [19,20]. Examination of exosome content in body fluids is a widely used method for biomarker investigation. Various studies currently focus on discovering miRNAs for use as potential biomarkers for stroke diagnosis and prognosis [21]; therefore, by examining the patient’s miRNA profile in the first hours and days after ischemic stroke, one could predict which processes (i.e., neuroprotection or neurodegeneration) will predominate in that patient [22,23,24,25].

Identifying parameters that diminish the clinical benefits of MT involves understanding the specific molecular processes linked to restoring blood flow in hypoxic areas and the mechanisms causing reperfusion injury. This understanding could aid in predicting clinical deficits in patients who have experienced an ischemic stroke, ultimately enhancing the guidance of stroke reperfusion treatment. The search for the most effective tools for stroke treatment has been ongoing for years. Incorporating patients’ miRNA profiles in the selection process for MT could assist neurologists in minimizing the risk of failure and enhancing the clinical outcomes of the procedure. This study is the first to report on changes in exosomal miRNA expression during the acute phase of stroke in patients who underwent different endovascular treatments. The clinical condition of patients can vary in the hours and days following a stroke. Therefore, we examined how individual therapies (antithrombotic, rt-PA or MT) or combined therapy (rt-PA/MT) influenced the exosomal miRNA profile 10 days after the stroke occurred. We also compared the levels of different miRNAs assessed on days 1 and 10 to the 10-day and 90-day mRS functional outcome scale (good functional outcome—mRS 0–2, and poor functional outcome—mRS 3–6). Finally, we conducted a functional analysis of the target genes of the miRNAs to determine their involvement in metabolic pathways. In order to identify and understand the biological role of miRNAs in various stroke treatments and patients’ functional outcomes, we examined how changes in miRNA expression profile induced by reperfusion might impact the estimated targets (ETs) and their relationship to ischemic stroke treatment.

This study is the first to analyze changes in exosomal miRNA expression during the acute phase of stroke in patients who received different endovascular treatments. Patients’ clinical condition can change in the hours and days after a stroke. We investigated how individual therapies (antithrombotic, rt-PA, or MT) or combined therapy (rt-PA/MT) affected the exosomal miRNA profile after 10 days post-stroke. We compared miRNA levels on days 1 and 10, with the 10-day and 90-day mRS functional outcome scale in stroke patients (good functional outcome—mRS 0–2, poor functional outcome—mRS 3–6). Additionally, we analyzed the target genes of the miRNAs to understand their role in metabolic pathways. By examining changes in miRNA expression induced by reperfusion, we aimed to understand their impact on estimated targets (ETs) and their relation to ischemic stroke treatment and patients’ functional outcomes.

## 2. Results

### 2.1. Study Design

**Figure 1:**
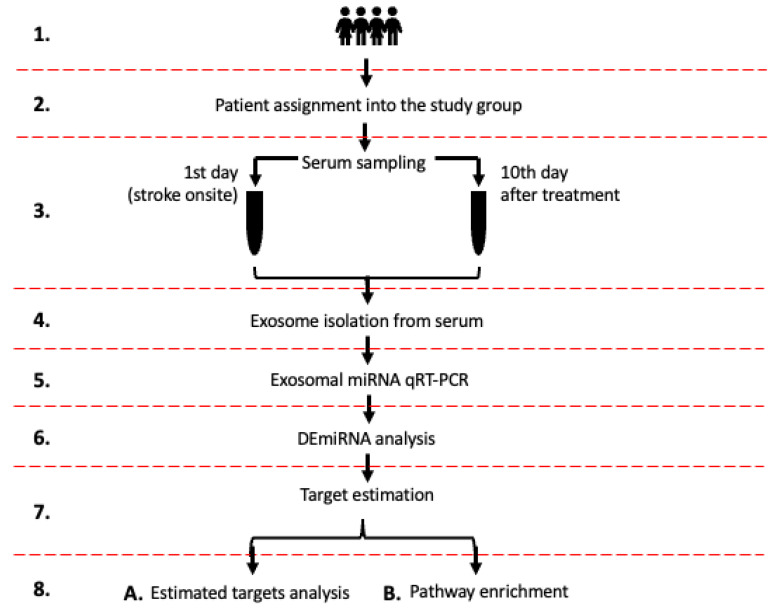
The flowchart illustrates the study design. (1) Total of 72 stroke patients included in the study; (2) Patient assignment into the study group: (i) aspirin, (ii) rt-PA, (iii) MT, and (iv) rt-Pa/MT; (3) blood sampling on 1st day (stroke onsite) and on 10th day (patient discharge), and serum isolation; (4) exosome isolation from serum samples; (5) exosomal miRNA qRT-PCR; (6) determining DEmiRNAs in each study group and between study groups; (7) miRNAs’ gene target estimation [27,28,29]; (8A) DEmiRNAs estimated targets analysis; (8B) DEmiRNAs enrichment analysis.

We examined 47 miRNAs in exosomes from serum samples from stroke patients on days 1 and 10 after stroke onset. These 47 miRNAs were selected based on the pilot analysis of exosomal miRNAs from human serum using Human TaqMan Advanced miRNA Array Cards A (Thermo Fisher Scientific, Waltham, USA), which contained 384 human miRNAs. MiRNAs well expressed in exosomes were selected for further analysis. Our objective was to identify specific miRNAs that exhibited varying regulation in patients who underwent different treatments (antithrombotic, rt-PA, MT, or rt-PA/MT combination) within first 10 days from stroke onset and had different functional outcomes (good or poor) as evaluated by the modified Rankin scale [26] on day 10 and 90 post-stroke. In addition, we conducted gene target estimation and enrichment analysis to determine the significant gene targets affected by these miRNAs and the biological pathways they are involved in. Figure 1 illustrates the study’s design, while Table 1 summarizes the patients’ comprehensive clinical characteristics that were analyzed in the study.

### 2.2. DEmiRNA Identification

In this study, we analyzed 47 selected miRNAs. These miRNAs included let-7g-5p, miR-15a-5p, miR-16-5p, miR-17-5p, miR-20a-5p, miR-21-5p, miR-23a-3p, miR-26b-5p, miR-30b-5p, miR-92a-3p, miR-93-5p, miR-103a-3p, miR-107, miR-125b-5p, miR-126-3p, miR-130a-3p, miR-142-3p, miR-143-3p, miR-148a-3p, miR-150-5p, miR-152-3p, miR-153-3p, miR-181c-5p, miR-185-5p, miR-186-5p, miR-193b-3p, miR-193a-5p, miR-199a-3p, miR-205-5p, miR-210-3p, miR-221-3p, miR-222-3p, miR-223-3p, miR-224-5p, miR-326, miR-339-5p, miR-342-3p, miR-361-5p, miR-376a-3p, miR-423-5p, miR-424-5p, miR-484, miR-486-5p, miR-505-3p, miR-576-5p, miR-652-3p, and miR-744-5p (Figure S1).

**Figure 2:**
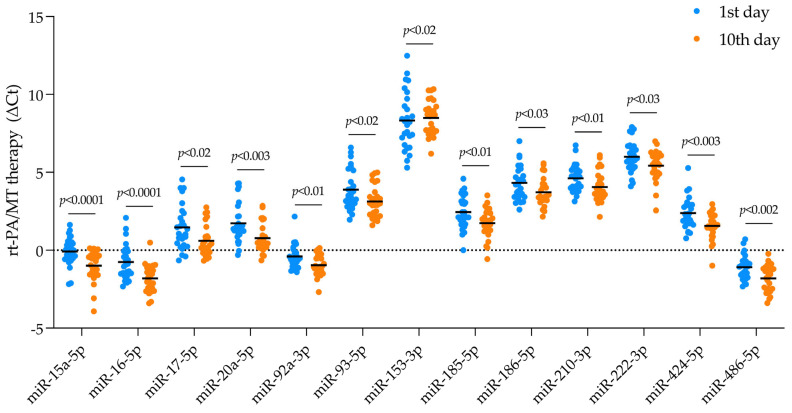
The expression of DEmiRNAs in patients receiving combined rt-PA/MT therapy. Dot plots represent individual values for presented miRNAs, where levels were significantly different between day 10 (orange dots) and day 1 (blue dots) (normalized to miR-320a). ∆Ct = Ct (target miRNA) − Ct (control miR-320a); *p*-value < 0.05. See Supplementary Table S19.

We compared the mean Ct levels of the studied miRNAs between day 10 and day 1 after ischemic stroke onset in each group (Table 2). No DEmiRNAs were identified in the group treated with the aspirin or MT alone. However, in the group treated with rt-PA alone, the level of miR-152-3p was higher on the 10th day compared to the 1st day after stroke. In contrast, when patients underwent combined treatment with both rt-PA/MT, we found 13 DEmiRNAs that were downregulated on the 10th day after stroke onset: miR-15a-5p, miR-16-5p, miR-17-5p, miR-20a-5p, miR-92a-3p, miR-93-5p, miR-152-3p, miR-153-3p, miR-185-5p, miR-186-5p, 210-3p, miR-222-3p, miR-424-5p, and miR-486-5p (Figure 2).

Next, we compared the levels of miRNAs (the fold change) between the studied groups (Table 3). In the analysis’s result, we found eight DEmiRNAs between studied groups; among them were miR-15a-5p, miR-16-5p, miR-17-5p, miR-142-3p, miR-152-3p, miR-486-5p, miR-505-3p, and miR-744-5p. The levels of miR-15a-5p, miR-17-5p, miR-16-5p, miR-142-3p, miR-486-5p and 505-3p were lower in the group treated with combined treatment (rt-PA/MT), but (i) miR-15a-5p and miR-17-5p compared to aspirin treatment, (ii) miR-15a-5p, miR-16-5p, miR-142-3p, miR-486-5p, and miR-505-3p compared to rt-PA treatment, and (iii) miR-505-3p compared to MT treatment. The level of miR-152-3p was significantly decreased in the group treated with MT compared to aspirin treatment, when miR-744-5p expression was significantly downregulated in groups treated with rt-PA or groups treated with MT when compared to aspirin.

### 2.3. Identifying DEmiRNA Related to Stroke Patient’s Functional Outcome

**Figure 3:**
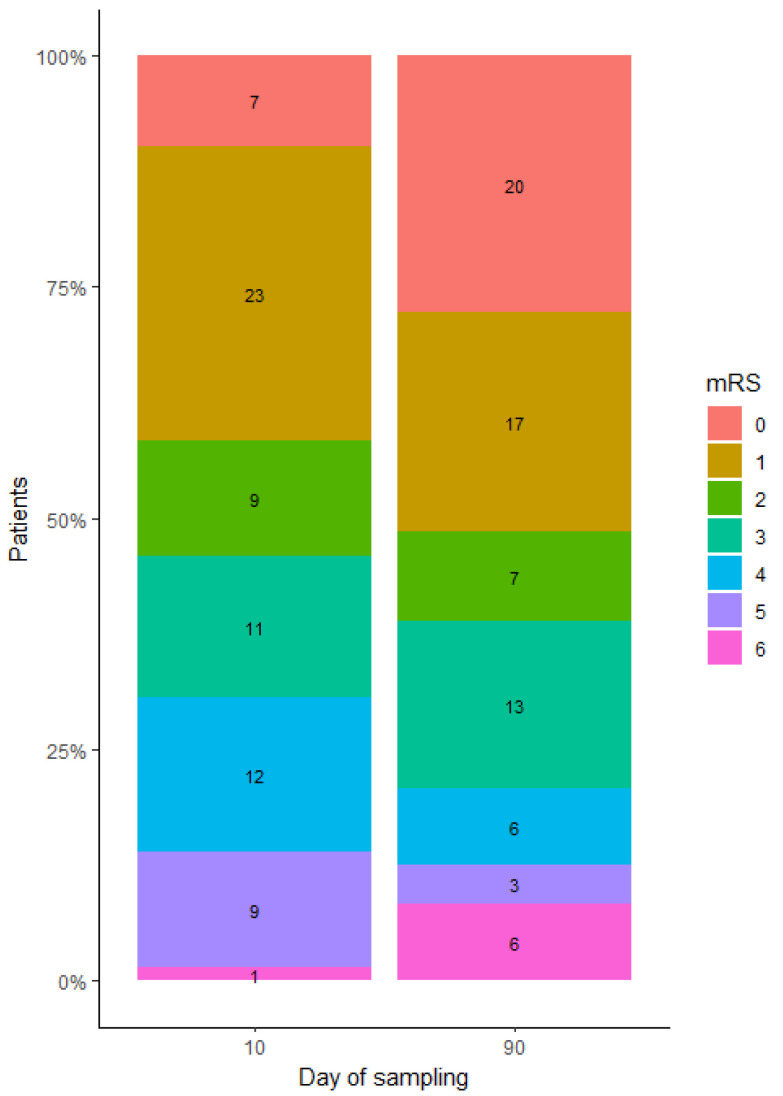
The distribution of 10-day mRS and 90-day mRS assessment among stroke patients. Each segment is colored differently to represent the score on the mRS scale (ranging from 0 to 6). The number within each segment corresponds to the number of patients who scored a specific number of points on the mRS scale during neurological evaluation.

We used the mRS scale to categorize patients as functionally poor (mRS ≥ 3) or functionally good (mRS < 3) based on their discharge (10-day mRS) and 3-month post-stroke functional status (90-day mRS). Of the study participants, 58.3% (42 patients) had a mRS score of 3 or higher at 10 days, compared to 48.6% (32 patients) at 90 days (Figure 3).

**Figure 4:**
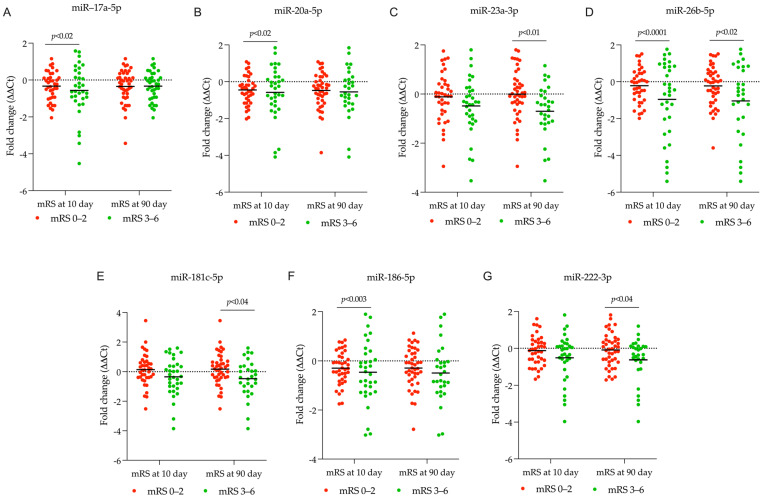
Identified DEmiRNA related to stroke patient’s functional outcome. Expression level of DEmiRNA within 10 days after stroke in patients categorized based on 10-day mRS and 90-day mRS score (mRS score 0–2, showed by red dots; mRS score 3–6, showed by green dots). Dot plots display the fold-change values (DDCt = DCt _miRNA on day 10_ − DCt _miRNA on day 1_) for the following identified DEmiRNAs: (**A**) miR-17a-5p, (**B**) miR-20a-5p, (**C**) miR-23a-3p, (**D**) miR-26b-5p, (**E**) miR-181c-5p, (**F**) miR-186-5p, and (**G**) miR-222-3p. *p*-value < 0.05. See Supplementary Table S20.

In the presented study, we found that a decline in miR-17-5p, miR-20a-5p, miR-186-5p, and miR-26b-5p levels between days 1 and 10 after a stroke was associated with patients’ 10-day mRS scores of 3–6, which is an indicator of a negative stroke outcome (Figure 4A,B,D,F, respectively). This downregulation of miR-17-5p, miR-20a-5p, and miR-186-5p was also observed in patients receiving rt-PA/MT therapy (Figure 2). We compared prevalent miRNA profiles (days 1–10 post-stroke) in patients experiencing poor functional outcomes three months later (90-day mRS 3–6) with good ones (90-day mRS 0–2), for predictive purposes. These patients exhibited downregulation of miR-23a-3p, miR-26b-5p, miR-181c-5p, and miR-222-3p in the first 10 days after stroke (Figure 4C–E,G, respectively). Patients treated with rt-PA/MT also showed decreased miR-222-3p levels (Figure 2).

### 2.4. Estimating Gene Targets for Differentially Expressed miRNAs and Their Enrichment Analysis

Our study design (Figure 1) involved estimating gene targets and performing enrichment analysis for each group. However, we only analyzed the rt-PA/MT patient group, as it was the only one with more than one differentially expressed miRNA.

#### 2.4.1. Estimating Gene Targets for DEmiRNA in Patients Receiving rt-PA/MT Treatment

In the group of patients receiving the rt-PA/MT treatment, we conducted target estimation analysis for the four DEmiRNAs with the highest fold-change: miR-15a-5p, miR-16-5p, miR-20a-5p, and miR-424-5p, using the MSigDB database [29]. We found that these DEmiRNAs collectively affect 1738 estimated gene targets. To narrow down the targets, we focused on the ones shared by these four DEmiRNAs (Figure S2A). Specifically, we found that miR-15a-5p, miR-16-5p, miR-20a-5p, and miR-424-5p have 64 shared distinct targets, including genes *ABHD2*, *ABL2*, *ACTR2*, *AGO4*, *APP*, *ARHGAP12*, *ATG14*, *ATXN7L3B*, *BTN3A3*, *BZW1*, *CAPZA2*, *CCND1*, *CCND2*, *CCDC88C*, *CHIC1*, *CLIP4*, *CNKSR3*, *CRIM1*, *CRK*, *DCTN5*, *DNAJC10*, *EIF2B2*, *ELK4*, *FOXK1*, *FZD9*, *HSPA4L*, *HSPA8*, *ITGA2*, *KIF23*, *KLHL15*, *LAMC1*, *L2HGDH*, *MAP2K3*, *MINK1*, *MTMR3*, *N4BP1*, *NUFIP2*, *OCRL*, *PHLPP2*, *PLRG1*, *PPP6R3*, *RAB3IP*, *RACGAP1*, *RFK*, *RPL14*, *RPRD2*, *SHOC2*, *SIK1*, *SMAD7*, *SNTB2*, *SOCS5*, *SPRED1*, *SSRP1*, *TMEM100*, *TMEM245*, *TNRC6B*, *TXNIP*, *USP48*, *WNK3*, *WEE1*, *VEGFA*, *YTHDC1*, and *ZMAT3*. Among brain diseases, only 37 ETs have been recorded, with stroke being the subject of 24 of them. In our literature research, we discovered 13 potential neuro-restoring ETs: *CAPZA2*, *KIF23*, *ACTR2*, *SHOC2*, *FZD9*, *CRIM1*, *LAMC1*, *YTHDC1*, *EIFB2*, *BZW1*, *SKI*, *HSPA8*, and *ATG14* (Table 4). The 21 remaining ETs, including *ARHGAP12*, *RAB3IP*, *ABL2*, *MAP2K3*, *WNK3*, *SIK1*, *AGO4*, *RPL14*, *VEGFR2*, *SPRED1*, *CRKL*, *ITGA2*, *CCDN1*, *ZMAT3*, *APP*, *SMAD7*, *SOC5*, *BTN3A3*, *RFK*, *TXNIP*, and *DNAJC10*, may contribute to neurodegeneration and lead to unfavorable functional outcomes (Table 4).

#### 2.4.2. Estimating Gene Targets for DEmiRNA in Patients Categorized Based on the 10-Day mRS Score

In our analysis, we estimated the gene targets of four differentially expressed miRNAs: miR-17a-5p, miR-20a-5p, miR-26b-5p, and miR-186-5p. These miRNAs showed significantly decreased expression levels within first 10 days in patients with a 10-day mRS score ≥ 3 compared to those with a 10-day mRS score < 3. Using the MSigDB database [29] for gene target estimation, we identified 2429 gene targets affected by these DEmiRNAs. To further refine our analysis, we focused on the targets shared by all four miRNAs (Figure S2B). Among the miR-17a-5p, miR-20a-5p, miR-26b-5p, and miR-186-5p, we identified 21 common targets: *ABHD2, BTG2, BTN3A3, CNOT4, CSNK1A1, DCBLD2, EIF4G2, MCL1, NUFIP1, PANK3, PLS1, PMAIP1, PPP1R15B, PTP4A1, RBM12B, RNASEH1, SLC28A1, SREK1IP1, VPS13C, ZNF426*, and *ZNF652.* Out of these, only six have been reported in brain diseases, with four specifically related to stroke: *MCL1*, *SLC28A1*, *BTG2*, *EIF4G2*, *PMAIP1*, and *RBM12B* (Table 4).

#### 2.4.3. Estimating Gene Targets for DEmiRNA in Patients Categorized Based on the 90-Day mRS Score

We conducted the gene target estimation analysis for miR-23a-3p, miR-26b-5p, miR-181c-5p, and miR-222-3p. These DEmiRNAs showed significantly decreased expression within first 10 days after stroke in patients with 90-day mRS scores ≥ 3 compared to patients with 90-day mRS scores < 3. From the gene target estimation analysis in the MSigDB database [29], we identified 3941 gene targets affected by these DEmiRNAs. We then focused on the targets that were common for these DEmiRNAs (Figure S2C). Among miR-23a-3p, miR-26b-5p, miR-181c-5p, and miR-222-3p, we found one shared target: *PTEN* (Table 4).

### 2.5. Analysis of the Enriched Pathways Targeted by DEmiRNAs Using Various Databases, Including PANTHER, Disease Alliance, HALLMARK, WIKI, KEGG, and Elsevier

#### 2.5.1. Analysis of the Enriched Pathways Targeted by DEmiRNAs in the Patients Receiving rt-PA/MT Treatment

**Figure 5:**
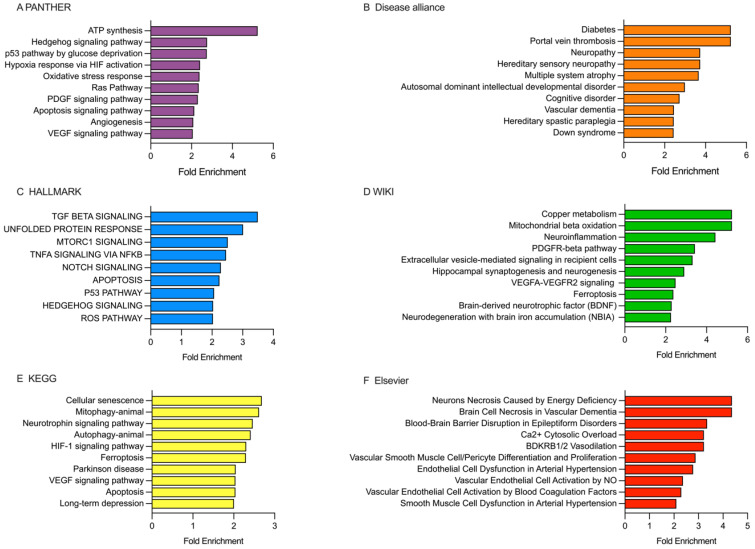
Enrichment analysis for patients receiving the rt-PA/MT treatment was performed using PANTHER, Disease alliance, HALLMARK, WIKI, KEGG, and Elsevier. (**A**) PANTHER’s gene terms. (**B**) The Disease alliance’s top terms. (**C**) HALLMARK’s top terms. (**D**) WIKI’s gene terms. (**E**) KEGG’s top terms. (**F**) Elsevier’s top terms. See Supplementary Tables S1–S6.

Enrichment analysis for rt-PA/MT group was performed for hsa-miR-15a-5p, hsa-miR-16-5p, hsa-miR-20a-5p, and hsa-miR-424-5p, which showed the highest fold change in the group. We used six open access databases: PANTHER, Disease alliance, HALLMARK, WIKI, KEGG, and Elsevier (Figure 5).

PANTHER database analysis showed that the ETs were enriched in various pathways such as ATP synthesis, Hedgehog pathway, p53 pathway by glucose deprivation, hypoxia response to HIF activation, oxidative stress response, PDGF signaling, apoptosis signaling, angiogenesis, and VEGF signaling (Figure 5A). The Disease alliance database revealed the ETs were primarily associated with conditions like diabetes, portal vein thrombosis, neuropathy, multiple system atrophy, cognitive disorder, and vascular dementia (Figure 5B). In the HALLMARK database, the ETs were found to be involved in pathways such as TGFβ signaling, unfolded protein response, mTORC1 signaling, TNFα signaling via NFκB, NOTCH signaling, apoptosis, p53 pathway, hedgehog signaling, and ROS pathway (Figure 5C). According to the WIKI database, the ETs were primarily associated with copper metabolism, mitochondrial β-oxidation, neuroinflammation, PDGFR-β pathway, extracellular vesicle-mediated signaling in recipient cells, hippocampal synaptogenesis and neurogenesis, VEGFA-VEGHR2 signaling, ferroptosis, BDNF, and neurodegeneration with brain iron accumulation (NBIA) (Figure 5D). In the KEGG database, the ETs were enriched in pathways such as cellular senescence, mitophagy, neurotrophin signaling, autophagy, HIF-1 signaling, ferroptosis, Parkinson’s disease, VEGF signaling, apoptosis, and long-term depression (Figure 5E). Lastly, the Elsevier database screening revealed that the ETs were enriched in pathways such as neurons necrosis caused by energy deficiency, brain cell necrosis by vascular dementia, blood-brain barrier disruption by epileptiform disorders, Ca^2+^ cytosolic overload, vascular smooth muscle cell/pericyte differentiation and proliferation, endothelial cell dysfunction in arterial hypertension, vascular endothelial cell activation by NO, and smooth muscle cell dysfunction in arterial hypertension (Figure 5F).

#### 2.5.2. Analysis of the Enriched Pathways Targeted by DEmiRNAs in Patients Categorized Based on the 10-Day mRS Score

**Figure 6:**
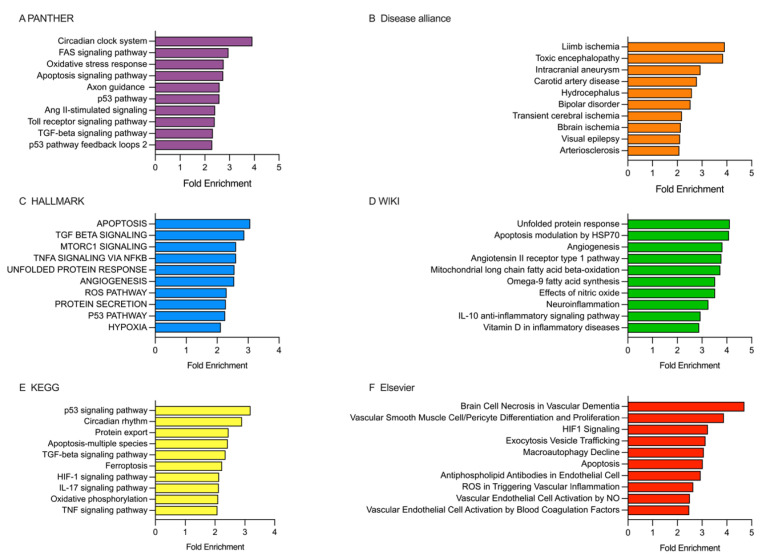
Analysis of the enriched pathways targeted by DEmiRNAs in patients categorized based on the 10-day mRS score, performed using PANTHER, Disease alliance, HALLMARK, WIKI, KEGG, and Elsevier. (**A**) PANTHER’s gene terms. (**B**) The Disease alliance’s gene terms. (**C**) HALLMARK’s gene terms. (**D**) WIKI’s gene terms. (**E**) KEGG’s gene terms. (**F**) Elsevier’s gene terms. See Supplementary Tables S7–S12.

Enrichment analysis (Figure 6) was conducted using the estimated targets (ETs) of DEmiRNAs in the first 10 days after stroke onset in patients categorized based on a 10-day mRS score (Figure 4).

PANTHER database analysis showed that ETs were enriched in various pathways, such as circadian clock system, FAS signaling, oxidative stress response, apoptosis signaling, axon guidance, p53 pathway, Ang II-stimulated signaling, Toll receptor signaling, TGF-beta signaling, and p53 pathway feedback loops 2 (Figure 6A). The Disease alliance database revealed ETs in conditions like intracranial aneurysm, carotid artery disease, bipolar disorder, transient cerebral ischemia, brain ischemia, and arteriosclerosis (Figure 6B). In the HALLMARK database, the ETs were involved in pathways like apoptosis, TGF-beta signaling, mTORC1 signaling, TNF-alpha signaling via NF-kB, unfolded protein response, angiogenesis, ROS pathway, protein secretion, p53 pathway, and hypoxia (Figure 6C). According to the WIKI database, the ETs primarily augmented pathways related to unfolded protein response, apoptosis modulation by HSP70, angiogenesis, angiotensin II receptor type 1 pathway, mitochondrial long-chain fatty acid beta-oxidation, omega-9 fatty acid synthesis, effects of nitric oxide, neuroinflammation, and Il-10 anti-inflammatory signaling pathway (Figure 6D). In the KEGG database, we found ETs enriched in pathways, such as p53 signaling, circadian rhythm, protein export, apoptosis, TGF-beta signaling, ferroptosis, HIF-1 signaling pathway, IL-17 signaling, oxidative phosphorylation, and TNF signaling (Figure 6E). Lastly, screening the Elsevier database revealed ETs enriched in pathways related to brain cell necrosis in vascular dementia, vascular smooth muscle cell/pericyte differentiation and proliferation, HIF-1 signaling, exocytosis vesicle trafficking, macroautophagy decline, apoptosis, ROS in triggering vascular inflammation, and vascular endothelial cell activation by blood coagulation factors (Figure 6F).

#### 2.5.3. Analysis of the Enriched Pathways Targeted by DEmiRNAs in Patients Categorized Based on the 90-Day mRS Score

**Figure 7:**
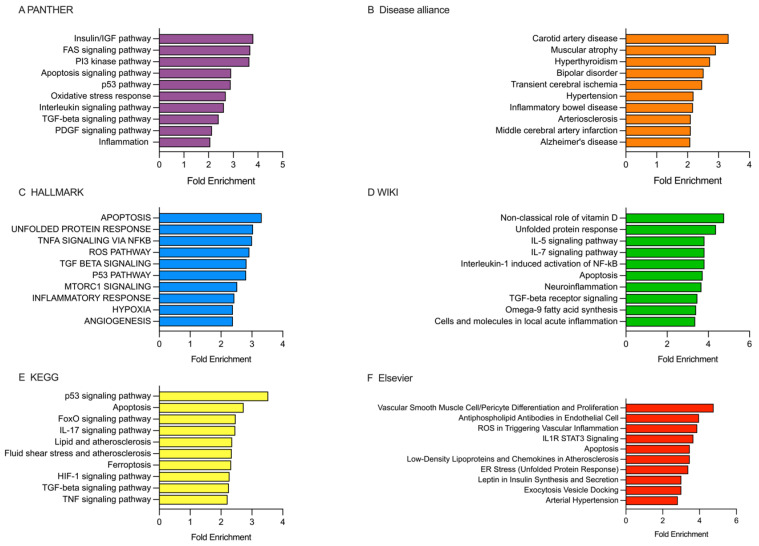
Analysis of the enriched pathways targeted by DEmiRNAs in patients categorized based on the 90-day mRS score, performed using PANTHER, Disease alliance, HALLMARK, WIKI, KEGG, and Elsevier. (**A**) PANTHER’s gene terms. (**B**) The Disease alliance’s gene terms. (**C**) HALLMARK’s gene terms. (**D**) WIKI’s gene terms. (**E**) KEGG’s gene terms. (**F**) Elsevier’s gene terms. See Supplementary Tables S13–S18.

Enrichment analysis (Figure 7) was conducted using the estimated targets (ETs) of DEmiRNAs in the first 10 days after stroke onset in patients categorized based on the 90-day mRS score (Figure 4).

PANTHER database analysis showed that the ETs were enriched in various pathways, including the insulin/IGF pathway, FAS signaling, PI3 kinase pathway, apoptosis signaling, p53 pathway, oxidative stress response, interleukin signaling pathway, TGFβ signaling, PDGF signaling, and inflammation (Figure 7A). The Disease alliance database revealed the ETs were associated with carotid artery disease, muscular atrophy, bipolar disorder, transient cerebral ischemia, hypertension, arteriosclerosis, middle cerebral artery infraction, and Alzheimer’s disease (Figure 7B). In the HALLMARK database, the ETs were found to be involved in pathways such as apoptosis, unfolded protein response, TNFα signaling via NFκB, ROS pathway, TGFβ signaling, p53 pathway, mTORC1 signaling, inflammatory response, hypoxia, and angiogenesis (Figure 7C). According to the WIKI database, the ETs were primarily associated with non-classical roles of vitamin D, unfolded protein response, IL-5 signaling, IL-7 signaling, Interleukin-1 induced activation of NFκB, apoptosis, neuroinflammation, TGFβ receptor signaling, omega-9 fatty acid synthesis, and cells and molecules in local acute inflammation (Figure 7D). In the KEGG database, the ETs were enriched in pathways such as p53 signaling, apoptosis, FoxO signaling, IL-17 signaling, lipid signaling, fluid shear stress and atherosclerosis, ferroptosis, HIF-1 signaling, TGFβ signaling, and TNF signaling (Figure 7E). Finally, in the Elsevier database, the ETs were enriched in pathways related to vascular smooth muscle cell/pericyte differentiation and proliferation, antiphospholipid antibodies in endothelial cells, ROS in triggering vascular inflammation, IL1R STAT3 signaling, apoptosis, low-density lipoproteins and chemokines in atherosclerosis, ER stress (unfolded protein response), leptin in insulin synthesis and secretion, and arterial hypertension (Figure 7F).

## 3. Discussion

The endovascular and pharmacological treatment for ischemic stroke has improved over the years, leading to a higher survival rate and better recovery prognosis for patients. However, there is still a high percentage of patients who do not recover or have a low recovery rate compared to others with similar clinical profiles. Currently, the main method of treating cerebral infarction is rt-PA, but its application is limited because of the short time window of 4.5 h or 9 h for precisely selected acute ischemic stroke (AIS) patients. Endovascular treatment (MT) has increased the recanalization rate of occluded vessels and extended the time window for stroke intervention to 24 h. The combined treatment of rt-PA/MT is considered the most effective, but the biological mechanism behind its effectiveness is not fully understood. The complex network of miRNAs, a type of non-coding RNA molecules, plays a role in neurological changes during and after ischemic stroke. Some miRNAs have been suggested as potential biomarkers for stroke risk assessment and early detection [77,78], but no studies have focused on the relevance of exosomal miRNAs in stroke therapy effectiveness or their role in patient enrollment for endovascular recanalization treatment or recovery rate. Further research is necessary to fully understand MT’s effects, particularly by analyzing the exosomal miRNA profile, given exosomes’ ability to transfer miRNA between cells and tissues via bodily fluids. Thus, gene expression may be impacted in organs both near and far from the brain.

The aim of this research project was to determine the impact of stroke treatment on specific miRNAs found in circulating blood exosomes. In addition, we assessed the miRNA that varied between patients of different functional outcomes at day 10 and 90 post-stroke. In this research, we integrated preliminary data of 47 exosomal miRNA’s expressions and functional outcomes in stroke patients after one of four different treatments: (i) antithrombotic therapy, (ii) rt-PA, (iii) MT and (iv) combined therapy of rt-PA/MT, with detailed bioinformatics–enrichment analyses.

Our study revealed a significant decrease in the expression levels of miR-15a-5p, miR-16-5p, miR-17-5p, miR-20a-5p, miR-92a-3p, miR-93-5p, miR-153-3p, miR-185-5p, miR-210-3p, miR-222-3p, miR-424-5p and miR-486-5p in the EVs of patients treated with rt-PA/MT upon their hospital discharge. The miR-152-3p was the only miRNA that showed a significant decrease in expression levels between the time of rt-PA treatment and the day of discharge. However, when comparing different treatment groups, the analysis showed different results of fold-change levels analysis for each miRNA. Compared to antithrombotic therapy, miR-15a-5p and miR-17-5p were lower in the rt-PA/MT group, miR-152-3p and miR-744-5p in MT group, and miR-744-5p in rt-PA group. Compared to rt-PA, we found decreased level of miR-15a-5p, miR-16-5p, miR-142-3p, miR-486-5p, and miR-505-3p in the rt-PA/MT group. Only the miR-505-3p expression level differed between the MT group and the rt-PA/MT group.

We compared the levels of specific miRNAs between the 1st and 10th day after stroke, and looked at the functional status of the patients at the 10th and 90th day after stroke.

Our study found that a decline in miR-17-5p, miR-20a-5p, miR-186-5p, and miR-26b-5p levels between days 1 and 10 after a stroke was associated with patients’ 10-day mRS scores of 3–6, which is an indicator of a negative stroke outcome. This downregulation of miR-17-5p, miR-20a-5p, and miR-186-5p was also observed in patients receiving rt-PA/MT therapy. We compared miRNA profiles (days 1–10 post-stroke) in patients experiencing poor functional outcomes three months later (90-day mRS 3–6) with good ones (90-day mRS 0–2), for predictive purposes. These patients exhibited downregulation of miR-23a-3p, miR-26b-5p, miR-181c-5p, and miR-222-3p in the first 10 days after stroke. Patients treated with rt-PA/MT also showed decreased miR-222-3p levels.

Previous research, primarily focused on diagnostics, had examined a subset of the miRNAs tested here within serum extracellular vesicles from ischemic stroke patients. These miRNAs include miR-15a-5p, miR-16-5p, miRNA-17-5p, miR-23a-3p, miR-93-5p, miR-152-3p, miR-186-5p, miR-424-5p, miR-505-3p, and miR-744-5p. Compared to healthy controls, the levels of miR-15a-5p [79], miR-152-3p [80], and miR-424 [79] decreased in serum EVs of IS patients. Conversely, the levels of miR-16-5p [81,82], miRNA-17-5p [83], miR-23a-3p [82], and miR-93-5p [83,84] in serum EVs were significantly increased. Notably, there was no difference in the levels of miR-186-5p [85], miR-505-3p, and miR-744-5p between IS patients and healthy controls [82].

Because of the scarcity of miRNA research on circulating EVs, we compared our serum EV stroke findings to those from studies using other blood sources in stroke patients.

However, the reported changes in miRNA levels varied significantly across stroke studies, likely because of differences in the blood samples used. We previously examined serum samples from stroke patients and found that miR-9-3p and miR-9-5p levels increased from day one to day ten after stroke in patients treated with reperfusion therapy [86]; nonetheless, these miRNAs were not found in exosomes in our different investigations, which focused on identifying miRNAs with significant expression in circulating exosomes [87]. In our analysis of existing research on miRNAs, we identified a comparable trend: the expression of miRNAs varied based on the blood sample type employed in the study. The levels of miR-15a-5p were found to be increased in serum from IS patients [88]. Conversely, miR-424-5p concentration was lower in the plasma of AIS patients [89], but higher in their serum compared to the control group [90]. In plasma, the level of miR-16-5p showed an increase [88,91]. Similar results were seen in the plasma [92] and PBMC [93] levels of miR-17-5p, as well as in serum levels [88], which were upregulated in IS patients. However, miR-93-5p levels in plasma and blood neutrophils of AIS patients were notably decreased compared to the control group [94]. Serum levels of miR-186-5p were upregulated in IS patients compared to healthy controls [95,96]. Interestingly, plasma miR-186-5p expression was significantly upregulated in IS patients with normal platelet activation on the first day after stroke onset. After seven days, the levels of plasma miR-186-5p significantly decreased in the same patients with normal platelet reactivity [85].

Even though the expression levels of miR-20a-5p, miR-23a-3p, miR-26b-5p, miR-92a-3p, miR-142-3p, miR-153-3p, miR-181c-5p, miR-185-5p, miR-210-3p, miR-222-3p, and miR-486-5p in serum extracellular vesicles (EVs) have not been studied, they were examined in various blood-related samples taken from patients with IS and compared to those from healthy individuals. There were no significant differences in the plasma levels of miR-20a-5p [97] or the serum levels of miR-23a [98] between IS patients and control subjects. However, in patients with acute ischemic stroke, the expression of plasma miR-26b-5p [81] was downregulated. The serum levels of miR-92a-3p were also reported to decrease in IS patients [81,99]. Interestingly, the use of miR-92a-3p showed high accuracy in diagnosing AIS patients [99]. In a different study, the expression of miR-142-3p in serum [100] and miR-181c-5p in plasma [101] was downregulated in stroke patients compared to healthy controls. Conversely, the serum [81,99] and plasma [92] levels of miR-185-5p increased in IS patients. Another study found a positive correlation between serum miR-185 and NIHSS or mRS score, as well as the area of cerebral infarction [90]. Among the studied miRs, miR-210 was the only one that was examined in time manner. It was found to decrease in serum between admission and 3 months after stroke [102,103]. However, in studies where whole-blood samples from IS patients were tested between 48 h and 10 days from stroke onset [104], and blood leukocytes at 7 and 14 days from stroke onset [105], high levels of miR-210 were correlated with good functional outcomes. In IS patients, the levels of miR-222 decreased in plasma [102]. Conversely, miR-486-5p in peripheral NK cells increased compared to healthy controls [106].

It is widely agreed that stroke is a complex condition with a polygenic nature. The findings from the enrichment analyses and DEmiRNAs target estimation correspond to stroke pathology. Within a 10-day timeframe following a stroke, the expression of all delineated DEmiRNAs diminished, which could subsequently induce upregulation of their targets associated with stroke pathology (see Table 4), encompassing both neurorestorative and neurodegenerative potentials. Stroke events induce significant cellular stress in the brain, resulting from an insufficient oxygen and glucose supply. Under stress, there is a marked increase in mitochondrial β-oxidation in response to glucose deficiency; simultaneously, oxidative stress response and cellular senescence pathways are activated, resulting in irreversible cellular arrest. Acute and delayed cell death inevitably occurs through a regulated apoptosis, ferroptosis because of ion accumulation, or uncontrolled necrosis. Autophagy, with its diverse mechanisms, is critical in post-stroke pathobiology. This may have pro-survival potential through the activation of chaperone proteins. However, when dysregulated, it may cause aberrant protein synthesis and accumulation, potentially causing delayed dementia in cognitive disorders such as Alzheimer’s and Parkinson’s disease, as well as mental disorders like depression and bipolar disorder. Conversely, differentially expressed genes can activate pathways that regulate cellular development through neuro- and synapto-genesis, re-networking of stroke-affected brain regions, and by augmenting synaptic plasticity. Concurrently with resolving post-stroke tissue response, angiogenesis facilitates vascular smooth muscle cell/pericyte differentiation and proliferation, which is vital for efficient blood perfusion to stroke-affected tissues, thereby protecting neural cells from demise; however, its immaturity may concurrently induce intracerebral brain hemorrhages. Analogous to autophagy, neuroinflammation is essential for an appropriate stroke response and the removal of stroke-related debris; however, its progression into chronic inflammation can cause severe secondary brain damage.

The ultimate functional outcome following a stroke is determined by the critical equilibrium between neurodegenerative and neuroprotective elements, influencing whether patients achieve full recovery or experience delayed neuropathy, multiple system atrophy, cognitive and psychological disorders, or secondary transient cerebral ischemia.

### Limitations

Our study has a few limitations. First, the were a limited number of patients (total n = 72) and small group sizes of each tested treatment. The individual variations in patients, like the patient’s medication dosage, economic situation, rehabilitation, and the patient’s mental condition between day 10 and day 90, may significantly affect the functional outcome evaluation on day 90. As a result, it may mask the biological effect of tested miRNA on the patient’s functional outcome in the presented preliminary study. Therefore, further studies should be performed on a bigger cohort of patients, together with sub-grouping patients according to their TICI (thrombolysis in cerebral infraction) scores, and a healthy control group to establish the baseline expression level of miRNAs. Second, the target estimation research was based on a recently published data package, but did not include the most recent studies. The estimated targets for analyzed DEmiRNAs were enriched by the analysis of the OMIC databases and need further in situ revalidation.

Although exosomes constitute a tiny fraction (0.0001%) of a cell’s volume, and represent only a small part of their parent cells, even minute alterations could significantly affect the intricate pathophysiology of ischemic stroke. The local abundance of miRNAs and their targets determine their ability to optimize cellular responses to abrupt or chronic changes, while miRNA expression can depend on many individual factors [107,108]. Therefore, further large-scale population studies are needed to confirm the clinical significance of identified DEmiRNAs.

## 4. Materials and Methods

This study included patients hospitalized at the Department of Neurology Upper Silesian Medical Center of the Silesian Medical University in Katowice between 2020 and 2022 because of stroke. The patients suffered from ischemic stroke related to large vessel occlusion, and were treated with aspirin (19 patients included in this study), or rt-PA (15 samples included in this study), or MT (11 patients included in this study), or both rt-PA /MT (27 patients included in this study) in ultra-acute stroke phase.

### 4.1. Inclusion Criteria

We included patients who met the main criteria: (1) age 50–85 years (2), first-ever symptomatic ischemic stroke diagnosed according to WHO definition and head CT and/or MRI result, (3) pre-stroke status of 0–2 mRankin, (4) no history of intracranial bleeding including the hemorrhagic transformation of the ischemic lesion, and (5) lack of other severe and/or disabling neurological disorders. All patients signed their informed consent. The main exclusion criteria were (1) pregnancy, (2) alcohol abuse/chronic use of psychostimulant, (3) brain tumor, (4) chronic infection/active neoplastic disease, (5) renal/hepatic failure, and (6) surgery in the last three months. The 72 patients who finally qualified into the study were divided into four groups: A total of 19 patients received antithrombotic therapy with aspirin; 15 patients received intravenous thrombolysis with alteplase (rt-PA); 11 patients underwent mechanical thrombectomy (MT); and 27 patients were treated with intravenous thrombolysis (alteplase) and mechanical thrombectomy (rt-PA/MT) (see flow-chart, Figure 1). The characteristics of the 72 qualified patients are presented in the Table 1.

### 4.2. Serum Sampling

Blood samples (5 mL) were collected twice from each patient by venipuncture into serum separator tubes (BD, Franklin Lakes, NJ, USA) on days 1 and 10 after the stroke onset. After incubation at room temperature for 30–45 min. to allow clotting, the samples were then centrifuged at 1940× *g* for 10 min at room temperature. The supernatant was collected and pipetted into aliquots (500 µL). Samples were stored at −80 °C until further analysis.

### 4.3. Exosome Isolation from Serum

Exosomes were isolated as described previously [87,108]. Briefly, exosomes were isolated using the Total Exosome Isolation kit (from serum) (Thermo Fisher Scientific, Waltham, MA, USA) according to manufacturer’s instruction. Serum samples were subjected to centrifugation at 2000× *g* for 30 min to pellet cellular debris. Then, supernatant containing the clarified serum (500 µL) was transferred to a new tube without disturbing the pellet, and placed on ice until ready to perform the isolation. Next, for precipitation, the Total Exosome Isolation kit was added to the cell and debris-free serum (1:2 with exosome isolation reagent and serum, respectively). Serum and the exosome isolation reagents were mixed by brief vortexing and incubated at 4 °C for 30 min before being centrifugated at 4 °C at 10,000× *g* for 1 h. The pellet containing pre-enriched exosomes was resuspended in 200 µL Exosome Resuspension Buffer (Thermo Fisher Scientific, Waltham, MA, USA). Exosomes isolated using this method expressed the exosome markers CD9 and CD63, and ranged in size between 30 to 200 nm, as shown elsewhere [87,109]. MiRNAs in exosomes are protected by a lipid bilayer and therefore resistant to degradation by circulating ribonucleases [110]. Because of the stable expression of the endogenous control miRNA-320a, stability of the miRNA in our exosomal preparations and between treatments can also be assumed.

### 4.4. Exosomal miRNA qPCR/RT

Total RNA was isolated using the Total Exosome RNA and Protein Isolation Kit (Thermo Fisher Scientific, Waltham, MA, USA) according to manufacturer’s instruction. 200 µL of exosome solution was mixed in 200 µL of 2× Denaturing Solution. Subsequently, 400 µL of Acid-Phenol:Chloroform was used for phase separation. Then, 312.5 µL of 100% ethanol was added on the aqueous phase (250 µL) from acid-phenol:chloroform extraction, and then mixed thoroughly. Finally, RNA was eluted in 50 µL of Elution Solution after being washed three times in RNA Wash Solution. Total RNA (2 µL) from the serum was reverse transcribed using the TaqMan^®^ Advanced miRNA Assays (Thermo Fisher Scientific, Waltham, MA, USA). TaqMan^TM^ Advanced miRNA Custom Array Cards for 48 selected miRNAs (Figure S1) were used with the TaqMan^®^ Fast Advanced Master Mix in the QuantStudio^TM^ 7 Flex Real-Time PCR System (Thermo Fisher Scientific, Waltham, MA, USA). The 48 miRNAs were selected based on the pilot analysis of 384 exosomal miRNAs using Human TaqMan Advanced miRNA Array Cards A (Thermo Fisher Scientific, Waltham, MA, USA). MiRNAs with robust expression levels in serum exosomes (Ct < 35) were selected [90].

### 4.5. MiRNAs Quantification and Differential Expression Analysis

MiRNA-320a was selected as an endogenous control based on stable expression between samples and the literature [90,111].

Data from Polymerase Chain Reaction was normalized to the endogenous miRNA-320a, and mean expression levels of normalized data were later used for all statistical analyses [112].

Statistical analysis was divided into three parts: analysis of DEmiRNAs between the 1st and 10th day post-stroke within each treatment group, analysis of DEmiRNAs fold-changes between treatment groups, and analysis of DEmiRNAs fold-changes in patients categorized based on the 10-day and 90-day mRS score, where good functional outcome was mRS < 3 and poor functional outcome was mRS ≥ 3, according to the modified Rankin Scale (mRS) [26].

We used Student’s *t*-tests (or Welch’s *t*-tests for non-normally distributed data) and Mann–Whitney U tests (for data that did not meet the assumptions of normality) to analyze differentially expressed miRNAs. Four-group comparisons were analyzed using one-way ANOVA with Tukey’s post hoc test if data were normally distributed; otherwise, Kruskal–Wallis tests with Dunn’s post hoc tests were employed. Normality of the data was assessed using the Shapiro–Wilk test, with the Q-Q plot used for a visual check to see if data transformation was required. Variance homogeneity was assessed with Levene’s test. A *p*-value below 0.05 was considered statistically significant. The significance of confounding variables, including diabetes mellitus, dyslipidemia, atrial fibrillation, and sex, was assessed using Spearman’s correlation and Multiple Linear Regression (Tables S19 and S20). No statistically significant findings were observed. This showed that the DEmiRNA findings were independent of confounding variables.

### 4.6. Estimation of DEmiRNAs Gene Targets and Enrichment Analysis

In part 2, we identified DEmiRNAs using ANOVA for parametric data with equal variances, Welch’s ANOVA for parametric data with unequal variances, and the Kruskal–Wallis test for non-parametric data lapping targets for DEmiRNAs in each analyzed group. We linked previously experimentally validated DEmiRNA targets and linked them to terms in the PANTHER (Protein ANalysis THrough Evolutionary Relationships) Classification System [113,114], DISEASE [115], MSig HALLMARK [29], WiKi Pathways [116], Kyoto Encyclopedia of Genes and Genomes (KEGG) [117,118] and Disease Pathways Elsevier databases [119]. To determine the *p*-value for enrichment analysis, the target occurrence number was compared to the occurrence expected by random chance. By setting the threshold at *p*-value < 0.05 and Enrichment FDR < 0.05, we could identify significant pathways of DEmiRNAs targets. A fold-change greater than or equal to 2 was significant according to the Fold-change-Specific Enrichment Analysis (FSEA) [86,120].

## 5. Conclusions

For the first time, our study showed that administering rt-PA/MT altered the exosomal miRNA profile in stroke patients, which might negatively impact their functional outcome. The levels of all the DEmiRNAs in this group were lower on the day of discharge compared to the day of admission, as well as compared to the fold-changes observed in the other study groups. Furthermore, the study revealed an association between reduced miRNA expression and negative stroke outcomes; patients with unfavorable functional outcomes at discharge presented with lower levels of miR-17, miR-20, miR-186, and miR-26b-5p. Similarly, patients with decreased levels of miR-222 at day 10 had a poor score on the mRS scale measured 90 days after suffering a stroke. The functional analysis revealed gene targets and enriched pathways associated with various processes, such as cytoskeleton remodeling, apoptosis, necrosis, ferroptosis, autophagy, inflammation, and dementia.

We would like to emphasize that it is unclear why only 50% of positive reperfusion therapy outcomes, as confirmed by post-thrombectomy angiography, result in a favorable patient status. Our aim has been to identify supplementary clinical and non-clinical parameters to refine patient selection for reperfusion therapy and potentially serve as targets for intervention during the initial phase following a stroke. This report details the potential correlation between the expression of specific mRNAs and the outcomes of post-stroke patients, with functional analysis indicating that certain processes may contribute to cell death and neurodegeneration. Further investigation is required to fully validate the relationship with larger patient cohorts.

## Figures and Tables

**Table 1:** Characteristics of the patients included in the study.

Baseline Demographic and Clinical Characteristics	
Cohort, n	72
Age, mean., med. [ref.]	70, 72.5 [35–93]
Gender M/F	37/35
BMI (kg/m^2^), med. [ref.]	27.5 [17–36]
Medical history	
Atrial Fibrillation	28 (39%)
Arterial Hypertension	65 (90%)
Diabetes Mellitus	25 (35%)
Coronary Artery Disease	21 (29%)
Peripheral Artery Disease	25 (35%)
Lipid Disorders	31 (43%)
Smoking	18 (25%)
Stroke Characteristics	
Occluded artery	
Left Middle cerebral artery	23 (32%)
Right Middle cerebral artery	14 (19%)
Left Internal carotid artery	4 (6%)
Basilar artery	1 (1%)
No thrombus	30 (42%)
Circulation territory (Oxfordshire community stroke project)	
Total anterior cerebral artery	20 (28%)
Partial anterior cerebral artery	23 (32%)
Lacunar infarct	23 (32%)
Posterior circulation infarct	6 (8%)
Stroke etiology (TOAST-trial of ORG 10172 in acute stroke treatment)	
Atherosclerosis	23 (32%)
Cardioembolism	29 (40%)
Small vessel occlusion	14 (19%)
Unknown/others origin of stroke	6 (8%)
Cohort Treatment	
Antithrombotic therapy (aspirin)	19 (26%)
Reperfusion therapy	
rt-Pa	17 (24%)
MT	9 (12.5%)
rt-Pa + MT	27 (27.5%)
Mechanical Thrombectomy	
MT (n = 36) Stent retriever Aspiration	25 (69%) 11 (31%)
Stroke onset-groin puncture, mean [ref.] min.	262 [140–360]
TICI (Treatment in cerebral infarction)	
0	4 (11%)
1	0 (0%)
2b	1 (3%)
2c	2 (5.5%)
3	29 (80.5%)
Successful recanalization (TICI 2b/2c/3)	32 (89%)
Blood Tests	
Day 1 [normal range]	
RBC_1	4.33 × 10^6^/µL [4.00–5.00]
WBC_1	9.68 × 10^3^/µL [4.00–10.00]
Lymphocyte_1	1.77 × 10^3^/µL [1.00–4.50]
Neutrophile_1	6.11 × 10^3^/µL [2.00–6.14]
Basophile_1	0.03 × 10^3^/µL [0.00–0.10]
Eosinophile_1	0.07 × 10^3^/µL [0.05–0.50]
PLT_1	200 × 10^3^/µL [135–350]
HCT_1	37.93% [36.00–47.00]
Hb_1	13.19 g/dL [12.00–16.00]
creatinine	2.39 mg/dL [0.51–0.95]
eGFR	73 mL/min/1.73 m^2^ [>60]
CRP	13 mg/L [<5.0]
Day 10 [normal range]	
RBC_10	4.36 × 10^6^/µL [4.00–5.00]
WBC_10	7.94 × 10^6^/µL [4.00–10.00]
PLT_10	272 × 103/µL [135–350]
HCT_10	38.17% [36.00–47.00]
Hb_10	13.12 g/dL [12.00–16.00]
Functional Outcome	
NIHSS day 1, med. [ref.]	9 [0–28]
NIHSS day 2	4 [0–28]
NIHSS day 10	2 [0–24]
10-day mRS, med. [ref.]	2 [0–6]
90-day mRS	1 [0–6]

rt-PA—recombinant tissue plasminogen activator, MT—mechanical thrombectomy, RBC_1—red blood cells on the 1st day, RBC_10—red blood cells on the 10th day, WBC_1—white blood cells on the 1st day, WBC_10—white blood cells on the 10th day, PLT_1—platelets on the 1st day, PLT_10—platelets on the 10th day, HCT_1—hematocrit on the 1st day, HCT_10—hematocrit on the 10th day, Hb_1—hemoglobin on the 1st day, Hb_10—hemoglobin on the 10th day, eGFR—estimated glomerular filtration rate, CRP—C-reactive protein, NIHSS—National Institutes of Health Stroke Scale, mRS—modified Rankin Scale, 10-day mRS—mRS at discharge, 90-day mRS. For laboratory tests, values in the square [ ] brackets show reference values for the presented parameter. In other cases, the square [ ] brackets enclose the lowest and highest value for a parameter assessed in patients included in the study.

**Table 2:** Mean Ct levels of miRNAs from day 1 and day 10 for ischemic stroke patients treated with aspirin, rt-PA, MT, or rt-PA with MT.

	TREATMENT (Mean Ct level)							
miRNA	Aspirin		rt-PA		MT		rt-PA + MT	
	Day		Day		Day		Day	
	1st	10th	1st	10th	1st	10th	1st	10th
let-7g-5p	4.90	4.83	5.08	4.80	5.02	4.45	5.21	4.60
**miR-15a-5p**	−0.07	−0.17	−0.17	−0.29	−0.43	−0.71	**−0.08**	**−0.99** *
**miR-16-5p**	−1.02	−1.30	−1.15	−1.29	−1.23	−1.38	**−0.76**	−1.80 *
**miR-17-5p**	1.15	1.17	1.25	1.10	1.40	0.67	**1.46**	0.61 *
**miR-20a-5p**	1.22	1.10	1.37	1.07	1.21	0.82	**1.73**	**0.78** *
miR-21-5p	0.64	0.78	0.87	0.68	0.27	−0.16	0.38	−0.10
miR-23a-3p	0.99	0.90	0.72	0.53	0.66	−0.14	0.53	0.22
miR-26b-5p	−0.09	0.00	0.32	−0.54	0.11	−0.78	0.17	−0.54
miR-30b-5p	1.37	1.13	1.41	1.06	1.58	0.97	1.78	1.05
**miR-92a-3p**	−0.51	−0.65	−0.58	−0.80	−0.70	−0.71	**−0.40**	**−0.96** *
**miR-93-5p**	3.87	3.52	3.98	3.67	3.75	3.46	**3.89**	**3.12** *
miR-103a-3p	3.29	2.77	3.06	3.01	3.23	2.68	3.11	2.49
miR-107	3.66	3.21	4.09	3.47	3.93	3.18	4.17	3.62
miR-125b-5p	5.84	5.86	6.11	5.59	5.46	4.90	5.24	4.77
miR-126-3p	0.25	0.48	0.16	0.11	−0.24	−1.06	0.17	0.03
miR-130a-3p	3.17	3.69	3.25	3.33	3.59	2.93	2.86	2.57
miR-142-3p	2.11	2.27	1.62	2.21	1.86	1.38	2.53	1.76
miR-143-3p	3.45	3.55	3.99	3.17	3.44	2.15	2.96	2.65
miR-148a-3p	3.61	3.73	3.77	3.74	3.31	3.05	3.09	2.80
miR-150-5p	2.25	2.20	2.22	1.88	3.81	1.97	2.37	2.03
**miR-152-3p**	4.31	4.64	**4.25**	**5.21** *	4.94	3.67	4.24	3.80
**miR-153-3p**	8.46	8.06	8.60	8.48	7.72	7.40	**8.32**	**8.49** *
miR-181c-5p	6.81	6.99	6.72	6.73	6.94	6.10	7.00	6.92
**miR-185-5p**	2.07	2.03	2.02	2.05	1.97	1.99	**2.45**	**1.74** *
**miR-186-5p**	4.14	4.06	4.08	3.79	4.11	3.64	**4.32**	**3.72** *
miR-193b-3p	6.82	6.44	6.64	6.43	5.63	5.82	5.87	5.96
miR-193a-5p	4.88	5.24	5.27	4.62	4.28	2.82	3.86	4.20
miR-199a-3p	2.69	3.02	2.89	2.62	3.04	1.78	2.53	2.37
miR-205-5p	2.13	1.96	0.39	0.52	2.27	1.34	1.43	1.07
**miR-210-3p**	4.22	4.68	4.92	4.85	4.21	4.28	**4.62**	**4.05** *
miR-221-3p	−0.01	0.21	−0.24	−0.41	−0.33	−0.91	−0.34	−0.47
**miR-222-3p**	6.27	6.43	6.48	6.33	5.98	5.23	**6.00**	**5.24** *
miR-223-3p	−0.87	−0.85	−0.61	−1.09	−0.65	−1.84	−0.62	−1.16
miR-224-5p	9.50	8.90	9.32	8.60	8.74	7.21	8.72	8.08
miR-326	2.34	2.86	2.18	2.04	2.40	1.93	2.12	2.09
miR-339-5p	3.60	3.74	3.86	3.54	3.70	3.02	3.53	3.07
miR-342-3p	5.20	5.26	5.51	5.45	5.77	4.76	5.56	5.06
miR-361-5p	5.79	5.71	5.80	5.50	5.63	4.57	5.67	5.16
miR-376a-3p	5.98	5.60	5.58	5.28	5.24	4.85	5.63	6.23
miR-423-5p	1.60	1.54	1.77	1.66	1.62	1.81	1.53	1.43
**miR-424-5p**	2.62	2.22	2.80	2.49	2.68	1.83	**2.38**	**1.56** *
miR-484	1.47	1.50	1.55	1.62	1.67	1.25	1.48	1.20
**miR-486-5p**	−1.46	−1.62	−1.42	−1.29	−1.60	−1.28	**−1.08**	**−1.80** *
miR-505-3p	6.71	6.77	6.17	6.70	4.79	5.66	6.07	5.25
miR-576-5p	2.97	2.78	2.82	2.35	2.76	4.25	3.34	2.81
miR-652-3p	3.70	3.68	3.79	3.48	3.64	3.21	3.73	3.46
miR-744-5p	2.36	2.76	2.66	2.13	2.14	1.45	2.70	2.64

Mean Ct from day 10th day vs. mean Ct from 1st day; * *p*-value < 0.05. Statistically significant comparisons within the treatment groups are indicated by bold formatting.

**Table 3:** MiRNAs fold-change levels for ischemic stroke patients treated with aspirin, rt-PA, MT, or rt-PA with MT.

	TREATMENT (deltaCt)			
miRNA	Aspirin	rt-PA	MT	rt-PA + MT
let-7g-5p	−0.0694	−0.2738	−0.5620	−0.6083
**miR-15a-5p**	**−0.1049**	**−0.1199**	−0.2750	−0.9136 ^A^*^,B^*
**miR-16-5p**	−0.2776	**−0.1423**	−0.1535	−1.0421 ^B^*
**miR-17-5p**	**0.0247**	−0.1487	−0.7379	−0.8489 ^A^*
miR-20a-5p	−0.1165	−0.2951	−0.3943	−0.9500
miR-21-5p	0.1431	−0.1907	−0.4275	−0.4769
miR-23a-3p	−0.0880	−0.1931	−0.8014	−0.3126
miR-26b-5p	0.0941	−0.8565	−0.8884	−0.7090
miR-30b-5p	−0.2360	−0.2725	−0.6944	−0.7352
miR-92a-3p	−0.1393	−0.2153	−0.0081	−0.5579
miR-93-5p	−0.3415	−0.3073	−0.2982	−0.7645
miR-103a-3p	−0.5179	−0.0470	−0.5547	−0.6271
miR-107	−0.4478	−0.6237	−0.7437	−0.5458
miR-125b-5p	0.0212	−0.5198	−0.5544	−0.4658
miR-126-3p	0.2287	−0.0438	−0.8228	−0.1455
miR-130a-3p	0.5158	0.0752	−0.4787	−0.8639
**miR-142-3p**	0.1614	**0.5838**	−0.4787	−0.8639 ^B^*
miR-143-3p	0.0969	−0.8183	−1.2904	−0.3064
miR-148a-3p	0.1146	−0.0216	−0.2655	−0.2943
miR-150-5p	−0.0463	−0.3419	−1.8395	−0.3348
**miR-152-3p**	**0.3355**	0.9672	−1.2705 ^D^*	−0.4368 ^B^*
miR-153-3p	−0.3953	−0.1152	−0.3229	0.1719
miR-181c-5p	0.1744	0.0159	−0.8334	−0.0790
miR-185-5p	−0.0397	0.0226	0.0298	−0.7151
miR-186-5p	−0.0813	−0.2855	−0.4653	−0.6009
miR-193b-3p	0.2956	−0.0798	−0.7962	0.4360
miR-193a-5p	−0.3834	−0.2044	0.1922	0.0928
cmiR-199a-3p	0.3269	−0.2650	−1.2600	−0.1582
miR-205-5p	0.4140	−0.7130	−0.1341	−0.5216
miR-210-3p	0.4585	−0.0682	0.0717	−0.5635
miR-221-3p	0.2189	−0.1704	−0.5812	−0.1273
miR-222-3p	0.1647	−0.1553	−0.7543	−0.5779
miR-223-3p	0.0165	−0.4776	−1.1839	−0.5409
miR-224-5p	−0.6883	−0.3592	−1.5274	−0.6447
miR-326	0.5234	−0.1400	−0.4710	−0.0317
miR-339-5p	0.1477	−0.3231	−0.6830	−0.4544
miR-342-3p	0.0605	−0.0595	−1.0074	−0.5013
miR-361-5p	−0.0882	−0.2986	−1.0626	−0.5139
miR-376a-3p	−0.3842	−0.3047	−0.3927	0.6082
miR-423-5p	−0.0533	−0.1089	0.1881	−0.0986
miR-424-5p	−0.3941	−0.3157	−0.8417	−0.8297
miR-484	0.0297	0.0716	−0.4219	−0.2730
**miR-486-5p**	−0.1620	**0.1333**	0.3238	−0.7204 ^B^*
**miR-505-3p**	0.0578	**0.5247**	**0.8710**	−0.8124 ^B^*^,C^*
miR-576-5p	−0.2973	−0.4956	−0.3756	−0.7959
miR-652-3p	−0.0238	−0.3095	−0.4288	−0.2717
**miR-744-5p**	**0.4028**	−0.5303 ^F^*	−0.6964 ^D^*	0.0582

Fold change (DCt = Ct _target miRNA_ − Ct _control miR-320a_); groups comparison order: ^A^ 3 vs. 0, ^B^ 3 vs. 1, ^C^ 3 vs. 2, ^D^ 2 vs. 0, ^E^ 2 vs. 1, ^F^ 1 vs. 0; * *p*-value < 0.05. Statistically significant comparisons between the treatment groups are indicated by bold formatting.

**Table 4:** Physiological effect of decreased expression of DEmiRNA via estimated targets (ET).

DEmiRNA Effect via Estimated Targets in Stroke		
General biological effect	Potential positive effect of targets on stroke-cell survival	Potential negative effect of targets on stroke-cell death
Neurogenesis, neurite outgrowth, neuronal re-networking, synaptic plasticity	*CAPZA2* [30], *KIF23* [31], *ACTR2* [32], *SHOC2* [33], *FZD9* [34], *CRIM1* [35,36], *LAMC1* [37], *SKI* [38], *EIFG2* [39]	
Oxidative stress response	*YTHDC1* 79, *EIF2B2* 80, *BZW1* 83, *MCL1* 138	*SIK1* [40]
Autophagy	*HSPA8* [41,42], *ATG14* [43]	
Neurite/synapse rejuvenation		*ARHGAP12* [44]
Acute/delayed cell death, apoptosis regulation	*MCL1* [45],	*RAB3IP* [46], *ABL2* [47,48], *MAP2K3* [49], *CCDN1* [50], *ZMAT3* [51], *PMAIP1* [52], *PTEN* [53]
Tissue ions and nucleotide homoeostasis	*SLC28A1* [54]	*WNK3* [55]
Translation regulation		*AGO4* [56]
Protein synthesis	*RBM12B* [57]	*RPL14* [58]
Angiogenesis		*VEGFR2* [59], *SPRED1* [60]
Platelets aggregation		*CRKL* [61], *ITGA2* [62,63]
Protein aggregation—dementia		*APP* [64,65], *SMAD7* [66], *DNAJC* [67,68],
Neuroinflammation	*BTG2* [69] 142	*SOC5* [70], *BTN3A3* [71], *RFK* [72,73], *TXNIP* [74,75], *PTEN* [76]

See the Abbreviation list for the full names of the genes.

## Data Availability

Data is contained within the article and Supplementary Materials.

## Supplementary Materials

The following supporting information can be downloaded at: https://www.mdpi.com/article/10.3390/ijms26199533/s1.

## Abbreviations

The following abbreviations are used in this manuscript:

ABL2	Abelson Tyrosine-Protein Kinase 2
ACTR2	Actin-Related Protein 2
AGO4	Argonaute RISC Component 4
APP	Amyloid Precursor Protein
ARHGAP12	Rho GTPase-Activating Protein 12
ATG14	Autophagy-Related Protein 14
BTG2	BTG Anti-Proliferation Factor 2
BTN3A3	Butyrophilin Subfamily 3 Member A3
BZW1	Basic Leucine Zipper and W2 Domains 1
CAPZA2	Capping Actin Protein of Muscle Z-Line Subunit Alpha 2
CCND1	Cyclin D1
CRIM1	Cysteine-Rich Motor Neuron 1
CRKL	CT10 Regulator of Kinase
DNAJC10	DnaJ Heat Shock Protein Family Member C10
EIF2B2	Eukaryotic Translation Initiation Factor 2B Subunit Beta
EIF4G2	Eukaryotic Translation Initiation Factor 4 Gamma 2
FZD9	Frizzled Class Receptor 9
HSPA8	Heat Shock Protein Family A (Hsp70) Member 8
ITGA2	Integrin Subunit Alpha 2
KIF23	Kinesin Family Member 23
LAMC1	Laminin Subunit Gamma 1
MAP2K3	Mitogen-Activated Protein Kinase 3
MCL1	Myeloid Cell Leukemia 1
PMAIP1	Phorbol-12-Myristate-13-Acetate-Induced Protein 1
PTEN	Phosphatase and Tensin Homolog
RAB3IP	RAB3A Interacting Protein
RBM12B	RNA Binding Motif Protein 12B
RFK	Riboflavin Kinase
RPL14	Ribosomal Protein L14
RPRD2	Regulation Of Nuclear Pre-MRNA Domain Containing 2
SHOC2	Leucine-Rich Repeat Protein SHOC2
SIK1	Salt-Inducible Kinase 1
SKI	c-ski protooncogene
SLC28A1	Solute Carrier Family 28 Member 1
SMAD7	SMAD Family Member 7
SOCS5	Suppressor Of Cytokine Signaling 5
SPRED1	Sprouty-Related, EVH1 Domain-Containing Protein 1
TXNIP	Thioredoxin Interacting Protein
VEGFR2	Vascular Endothelial Growth Factor Receptor 2
WNK3	WNK Lysine Deficient Protein Kinase 3
YTHDC1	YTH Domain Containing 1
ZMAT3	Zinc Finger Matrin-Type 3

## References

[1] Murray C.J.L., Afshin A., Alam T., Ashbaugh C., Barthelemy C., Biehl M., Brauer M., Compton K., Cromwell E., Dandona L. (2020). Global Burden of 369 Diseases and Injuries in 204 Countries and Territories, 1990–2019: A Systematic Analysis for the Global Burden of Disease Study 2019. Lancet.

[2] El Nawar R., Lapergue B., Piotin M., Gory B., Blanc R., Consoli A., Rodesch G., Mazighi M., Bourdain F., Kyheng M. (2019). Higher Annual Operator Volume Is Associated with Better Reperfusion Rates in Stroke Patients Treated by Mechanical Thrombectomy: The ETIS Registry. JACC Cardiovasc. Interv..

[3] Froehler M.T., Saver J.L., Zaidat O.O., Jahan R., Aziz-Sultan M.A., Klucznik R.P., Haussen D.C., Hellinger F.R., Yavagal D.R., Yao T.L. (2017). Interhospital Transfer Before Thrombectomy Is Associated with Delayed Treatment and Worse Outcome in the STRATIS Registry (Systematic Evaluation of Patients Treated with Neurothrombectomy Devices for Acute Ischemic Stroke). Circulation.

[4] Kaesmacher J., Gralla J., Mosimann P.J., Zibold F., Heldner M.R., Piechowiak E., Dobrocky T., Arnold M., Fischer U., Mordasini P. (2018). Reasons for Reperfusion Failures in Stent-Retriever-Based Thrombectomy: Registry Analysis and Proposal of a Classification System. AJNR Am. J. Neuroradiol..

[5] Coutinho J.M., Liebeskind D.S., Slater L.A., Nogueira R.G., Clark W., Dávalos A., Bonafé A., Jahan R., Fischer U., Gralla J. (2017). Combined Intravenous Thrombolysis and Thrombectomy vs Thrombectomy Alone for Acute Ischemic Stroke: A Pooled Analysis of the SWIFT and STAR Studies. JAMA Neurol..

[6] Goyal M., Menon B.K., Van Zwam W.H., Dippel D.W.J., Mitchell P.J., Demchuk A.M., Dávalos A., Majoie C.B.L.M., Van Der Lugt A., De Miquel M.A. (2016). Endovascular Thrombectomy After Large-Vessel Ischaemic Stroke: A Meta-Analysis of Individual Patient Data from Five Randomised Trials. Lancet.

[7] Mistry E.A., Mistry A.M., Nakawah M.O., Chitale R.V., James R.F., Volpi J.J., Fusco M.R. (2017). Mechanical Thrombectomy Outcomes with and Without Intravenous Thrombolysis in Stroke Patients: A Meta-Analysis. Stroke.

[8] Kim J.T., Liebeskind D.S., Jahan R., Menon B.K., Goyal M., Nogueira R.G., Pereira V.M., Gralla J., Saver J.L. (2018). Impact of Hyperglycemia According to the Collateral Status on Outcomes in Mechanical Thrombectomy. Stroke.

[9] Goyal N., Tsivgoulis G., Pandhi A., Dillard K., Katsanos A.H., Magoufis G., Chang J.J., Zand R., Hoit D., Safouris A. (2018). Admission Hyperglycemia and Outcomes in Large Vessel Occlusion Strokes Treated with Mechanical Thrombectomy. J. Neurointerv. Surg..

[10] Broocks G., Kemmling A., Aberle J., Kniep H., Bechstein M., Flottmann F., Leischner H., Faizy T.D., Nawabi J., Schön G. (2020). Elevated Blood Glucose Is Associated with Aggravated Brain Edema in Acute Stroke. J. Neurol..

[11] Zhang Y.H., Shi M.C., Wang Z.X., Li C., Sun M.Y., Zhou J., Zhang W.B., Huo L.W., Wang S.C. (2021). Factors Associated with Poor Outcomes in Patients Undergoing Endovascular Therapy for Acute Ischemic Stroke Due to Large-Vessel Occlusion in Acute Anterior Circulation: A Retrospective Study. World Neurosurg..

[12] Fischer U., Kaesmacher J., Strbian D., Eker O., Cognard C., Plattner P.S., Bütikofer L., Mordasini P., Deppeler S., Pereira V.M. (2022). Thrombectomy Alone versus Intravenous Alteplase plus Thrombectomy in Patients with Stroke: An Open-Label, Blinded-Outcome, Randomised Non-Inferiority Trial. Lancet.

[13] Mitchell P.J., Yan B., Churilov L., Dowling R.J., Bush S.J., Bivard A., Huo X.C., Wang G., Zhang S.Y., Ton M.D. (2022). Endovascular Thrombectomy versus Standard Bridging Thrombolytic with Endovascular Thrombectomy within 4·5 h of Stroke Onset: An Open-Label, Blinded-Endpoint, Randomised Non-Inferiority Trial. Lancet.

[14] Albers G.W., Marks M.P., Kemp S., Christensen S., Tsai J.P., Ortega-Gutierrez S., McTaggart R.A., Torbey M.T., Kim-Tenser M., Leslie-Mazwi T. (2018). Thrombectomy for Stroke at 6 to 16 Hours with Selection by Perfusion Imaging. N. Engl. J. Med..

[15] Jovin T.G., Saver J.L., Ribo M., Pereira V., Furlan A., Bonafe A., Baxter B., Gupta R., Lopes D., Jansen O. (2017). Diffusion-Weighted Imaging or Computerized Tomography Perfusion Assessment with Clinical Mismatch in the Triage of Wake up and Late Presenting Strokes Undergoing Neurointervention with Trevo (DAWN) Trial Methods. Int. J. Stroke.

[16] Powers W.J., Rabinstein A.A., Ackerson T., Adeoye O.M., Bambakidis N.C., Becker K., Biller J., Brown M., Demaerschalk B.M., Hoh B. (2019). Guidelines for the Early Management of Patients with Acute Ischemic Stroke: 2019 Update to the 2018 Guidelines for the Early Management of Acute Ischemic Stroke: A Guideline for Healthcare Professionals from the American Heart Association/American Stroke Association. Stroke.

[17] Chevillet J.R., Kang Q., Ruf I.K., Briggs H.A., Vojtech L.N., Hughes S.M., Cheng H.H., Arroyo J.D., Meredith E.K., Gallichotte E.N. (2014). Quantitative and Stoichiometric Analysis of the MicroRNA Content of Exosomes. Proc. Natl. Acad. Sci. USA.

[18] Krylova S.V., Feng D. (2023). The Machinery of Exosomes: Biogenesis, Release, and Uptake. Int. J. Mol. Sci..

[19] Hong S.B., Yang H., Manaenko A., Lu J., Mei Q., Hu Q. (2019). Potential of Exosomes for the Treatment of Stroke. Cell Transpl..

[20] Sun X., Jung J.H., Arvola O., Santoso M.R., Giffard R.G., Yang P.C., Stary C.M. (2019). Stem Cell-Derived Exosomes Protect Astrocyte Cultures from In Vitro Ischemia and Decrease Injury as Post-Stroke Intravenous Therapy. Front. Cell Neurosci..

[21] Sun A., Blecharz-Lang K.G., Małecki A., Meybohm P., Nowacka-Chmielewska M.M., Burek M., Decleces X., Vazquez Villasenor I. (2022). Role of MicroRNAs in the Regulation of Blood-Brain Barrier Function in Ischemic Stroke and Under Hypoxic Conditions In Vitro. Front. Drug Deliv..

[22] Vemuganti R. (2013). All’s Well That Transcribes Well: Non-Coding RNAs and Post-Stroke Brain Damage. Neurochem. Int..

[23] Long G., Wang F., Li H., Yin Z., Sandip C., Lou Y., Wang Y., Chen C., Wang D.W. (2013). Circulating MiR-30a, MiR-126 and Let-7b as Biomarker for Ischemic Stroke in Humans. BMC Neurol..

[24] Shah J.S., Soon P.S., Marsh D.J. (2016). Comparison of Methodologies to Detect Low Levels of Hemolysis in Serum for Accurate Assessment of Serum MicroRNAs. PLoS ONE.

[25] Motameny S., Wolters S., Nürnberg P., Schumacher B. (2010). Next Generation Sequencing of MiRNAs—Strategies, Resources and Methods. Genes.

[26] Weisscher N., Vermeulen M., Roos Y.B., de Haan R.J. (2008). What Should Be Defined as Good Outcome in Stroke Trials; a Modified Rankin Score of 0-1 or 0-2?. J. Neurol..

[27] Subramanian A., Tamayo P., Mootha V.K., Mukherjee S., Ebert B.L., Gillette M.A., Paulovich A., Pomeroy S.L., Golub T.R., Lander E.S. (2005). Gene Set Enrichment Analysis: A Knowledge-Based Approach for Interpreting Genome-Wide Expression Profiles. Proc. Natl. Acad. Sci. USA.

[28] Liberzon A., Subramanian A., Pinchback R., Thorvaldsdottir H., Tamayo P., Mesirov J.P. (2011). Molecular Signatures Database (MSigDB) 3.0. Bioinformatics.

[29] Liberzon A., Birger C., Thorvaldsdóttir H., Ghandi M., Mesirov J.P., Tamayo P. (2015). The Molecular Signatures Database Hallmark Gene Set Collection. Cell Syst..

[30] Fan Y., Tang X., Vitriol E., Chen G., Zheng J.Q. (2011). Actin Capping Protein Is Required for Dendritic Spine Development and Synapse Formation. J. Neurosci..

[31] Yu W., Cook C., Sauter C., Kuriyama R., Kaplan P.L., Baas P.W. (2000). Depletion of a Microtubule-Associated Motor Protein Induces the Loss of Dendritic Identity. J Neurosci.

[32] Spence E.F., Kanak D.J., Carlson B.R., Soderling S.H. (2016). The Arp2/3 Complex Is Essential for Distinct Stages of Spine Synapse Maturation, Including Synapse Unsilencing. J. Neurosci..

[33] Leon G., Sanchez-Ruiloba L., Perez-Rodriguez A., Gragera T., Martinez N., Hernandez S., Anta B., Calero O., Garcia-Dominguez C.A., Dura L.M. (2014). Shoc2/Sur8 Protein Regulates Neurite Outgrowth. PLoS ONE.

[34] Ramírez V.T., Ramos-Fernández E., Henríquez J.P., Lorenzo A., Inestrosa N.C. (2016). Wnt-5a/Frizzled9 Receptor Signaling through the Gαo-Gβγ Complex Regulates Dendritic Spine Formation. J. Biol. Chem..

[35] Zedde M., Grisendi I., Assenza F., Napoli M., Moratti C., Di Cecco G., D’Aniello S., Valzania F., Pascarella R. (2024). Stroke-Induced Secondary Neurodegeneration of the Corticospinal Tract—Time Course and Mechanisms Underlying Signal Changes in Conventional and Advanced Magnetic Resonance Imaging. J. Clin. Med..

[36] Sahni V., Itoh Y., Shnider S.J., Macklis J.D. (2021). Crim1 and Kelch-like 14 Exert Complementary Dual-Directional Developmental Control over Segmentally Specific Corticospinal Axon Projection Targeting. Cell Rep..

[37] Wu Q., Yuan K., Yao Y., Yao J., Shao J., Meng Y., Wu P., Shi H. (2024). LAMC1 Attenuates Neuronal Apoptosis via FAK/PI3K/AKT Signaling Pathway After Subarachnoid Hemorrhage. Exp. Neurol..

[38] Zhai Y., Ye S.Y., Wang Q.S., Xiong R.P., Fu S.Y., Du H., Xu Y.W., Peng Y., Huang Z.Z., Yang N. (2022). Overexpressed Ski Efficiently Promotes Neurorestoration, Increases Neuronal Regeneration, and Reduces Astrogliosis After Traumatic Brain Injury. Gene Ther..

[39] Hacisuleyman E., Hale C.R., Noble N., Luo J.D., Fak J.J., Saito M., Chen J., Weissman J.S., Darnell R.B. (2024). Neuronal Activity Rapidly Reprograms Dendritic Translation via EIF4G2:UORF Binding. Nat. Neurosci..

[40] Cheng J., Uchida M., Zhang W., Grafe M.R., Herson P.S., Hurn P.D. (2011). Role of Salt-Induced Kinase 1 in Androgen Neuroprotection against Cerebral Ischemia. J. Cereb. Blood Flow. Metab..

[41] Mohanan A., Deshpande S., Jamadarkhana P.G., Kumar P., Gupta R.C., Chauthaiwale V., Dutt C. (2011). Delayed Intervention in Experimental Stroke with TRC051384—A Small Molecule HSP70 Inducer. Neuropharmacology.

[42] Zhan X., Kim C., Sharp F.R. (2008). Very Brief Focal Ischemia Simulating Transient Ischemic Attacks (TIAs) Can Injure Brain and Induce Hsp70 Protein. Brain Res..

[43] Wang P., Shao B.Z., Deng Z., Chen S., Yue Z., Miao C.Y. (2018). Autophagy in Ischemic Stroke. Prog. Neurobiol..

[44] Deshpande S.S., Malik S.C., Conforti P., di Lin J., Chu Y.H., Nath S., Greulich F., Dumbach M.A., Uhlenhaut N.H., Schachtrup C. (2022). P75 Neurotrophin Receptor Controls Subventricular Zone Neural Stem Cell Migration after Stroke. Cell Tissue Res..

[45] Xingyong C., Xicui S., Huanxing S., Jingsong O., Yi H., Xu Z., Ruxun H., Zhong P. (2013). Upregulation of Myeloid Cell Leukemia-1 Potentially Modulates Beclin-1-Dependent Autophagy in Ischemic Stroke in Rats. BMC Neurosci..

[46] Chen F., Han J., Li X., Zhang Z., Wang D. (2021). Identification of the Biological Function of MiR-9 in Spinal Cord Ischemia-Reperfusion Injury in Rats. PeerJ.

[47] Yan C., Yu H., Liu Y., Wu P., Wang C., Zhao H., Yang K., Shao Q., Zhong Y., Zhao W. (2021). C-Abl Tyrosine Kinase-Mediated Neuronal Apoptosis in Subarachnoid Hemorrhage by Modulating the LRP-1-Dependent Akt/GSK3β Survival Pathway. J. Mol. Neurosci..

[48] Motaln H., Rogelj B. (2023). The Role of C-Abl Tyrosine Kinase in Brain and Its Pathologies. Cells.

[49] Yao X., Wang Y., Zhang D. (2018). MicroRNA-21 Confers Neuroprotection Against Cerebral Ischemia-Reperfusion Injury and Alleviates Blood-Brain Barrier Disruption in Rats via the MAPK Signaling Pathway. J. Mol. Neurosci..

[50] Liu Y., Shang G., Zhang X., Liu F., Zhang C., Li Z., Jia J., Xu Y., Zhang Z., Yang S. (2022). CAMTA1 Gene Affects the Ischemia-Reperfusion Injury by Regulating CCND1. Front. Cell Neurosci..

[51] Tomasevic G., Shamloo M., Israeli D., Wieloch T. (1999). Activation of P53 and Its Target Genes P21WAF1/Cip1 and PAG608/Wig-1 in Ischemic Preconditioning. Mol. Brain Res..

[52] Shibue T., Suzuki S., Okamoto H., Yoshida H., Ohba Y., Takaoka A., Taniguchi T. (2006). Differential Contribution of Puma and Noxa in Dual Regulation of P53-Mediated Apoptotic Pathways. EMBO J..

[53] Li X., An P., Han F., Yu M., Yu Z., Li Y. (2023). Silencing of YTHDF1 Attenuates Cerebral Stroke by Inducing PTEN Degradation and Activating the PTEN/AKT/MTOR Pathway. Mol. Biotechnol..

[54] Schädlich I.S., Winzer R., Stabernack J., Tolosa E., Magnus T., Rissiek B. (2023). The Role of the ATP-Adenosine Axis in Ischemic Stroke. Semin. Immunopathol..

[55] Begum G., Yuan H., Kahle K.T., Li L., Wang S., Shi Y., Shmukler B.E., Yang S.S., Lin S.H., Alper S.L. (2015). Inhibition of WNK3 Kinase Signaling Reduces Brain Damage and Accelerates Neurological Recovery after Stroke. Stroke.

[56] Koester S.K., Dougherty J.D. (2022). A Proposed Role for Interactions between Argonautes, MiRISC, and RNA Binding Proteins in the Regulation of Local Translation in Neurons and Glia. J. Neurosci..

[57] Trollmann R., Rehrauer H., Schneider C., Krischke G., Huemmler N., Keller S., Rascher W., Gassmann M. (2010). Late-Gestational Systemic Hypoxia Leads to a Similar Early Gene Response in Mouse Placenta and Developing Brain. Am. J. Physiol. Regul. Integr. Comp. Physiol..

[58] Johnson E.C.B., Dammer E.B., Duong D.M., Ping L., Zhou M., Yin L., Higginbotham L.A., Guajardo A., White B., Troncoso J.C. (2020). Large-Scale Proteomic Analysis of Alzheimer’s Disease Brain and Cerebrospinal Fluid Reveals Early Changes in Energy Metabolism Associated with Microglia and Astrocyte Activation. Nat. Med..

[59] Hu Y., Huang S., Shen T., Wang R., Geng M., Wang Y., Zheng Y., Luo Y., Li S. (2024). Prognostic Significance of Plasma VEGFA and VEGFR2 in Acute Ischemic Stroke-a Prospective Cohort Study. Mol. Neurobiol..

[60] Wei L., Li X., Wei Q., Chen L., Xu L., Zhou P. (2023). Oxidative Stress-Mediated Sprouty-Related Protein with an EVH1 Domain 1 Down-Regulation Contributes to Resisting Oxidative Injury in Microglia. Neuroscience.

[61] Cevik O., Baykal A.T., Sener A. (2016). Platelets Proteomic Profiles of Acute Ischemic Stroke Patients. PLoS ONE.

[62] Al-Qaisi R.M., Al-Ani L., Al-Halbosiy M.M.F. (2018). Genetic Polymorphism of ITGA2 Gene and the Risk of Heart Attack and Stroke in Al-Anbar Population/Iraq. J. Pharm. Sci. Res..

[63] Lu J.X., Lu Z.Q., Zhang S.L., Zhi J., Chen Z.P., Wang W.X. (2014). Polymorphism in Integrin ITGA2 Is Associated with Ischemic Stroke and Altered Serum Cholesterol in Chinese Individuals. Balk. Med. J..

[64] Mendes F.R., Leclerc J.L., Liu L., Kamat P.K., Naziripour A., Hernandez D., Li C., Ahmad A.S., Doré S. (2020). Effect of Experimental Ischemic Stroke and PGE2 EP1 Selective Antagonism in Alzheimer’s Disease Mouse Models. J. Alzheimer’s Dis..

[65] Goulay R., Mena Romo L., Hol E.M., Dijkhuizen R.M. (2020). From Stroke to Dementia: A Comprehensive Review Exposing Tight Interactions Between Stroke and Amyloid-β Formation. Transl. Stroke Res..

[66] Kashima R., Hata A. (2018). The Role of TGF-β Superfamily Signaling in Neurological Disorders. Acta Biochim. Biophys. Sin..

[67] Zhang H., Cao Y., Ma L., Wei Y., Li H. (2021). Possible Mechanisms of Tau Spread and Toxicity in Alzheimer’s Disease. Front. Cell Dev. Biol..

[68] Montibeller L., Tan L.Y., Kim J.K., Paul P., de Belleroche J. (2020). Tissue-Selective Regulation of Protein Homeostasis and Unfolded Protein Response Signalling in Sporadic ALS. J. Cell Mol. Med..

[69] Suzuki K., Shinohara M., Uno Y., Tashiro Y., Gheni G., Yamamoto M., Fukumori A., Shindo A., Mashimo T., Tomimoto H. (2021). Deletion of B-Cell Translocation Gene 2 (BTG2) Alters the Responses of Glial Cells in White Matter to Chronic Cerebral Hypoperfusion. J. Neuroinflammation.

[70] Zhao Y., Li T., Jiang Z., Gai C., Yu S., Xin D., Li T., Liu D., Wang Z. (2024). The MiR-9-5p/CXCL11 Pathway Is a Key Target of Hydrogen Sulfide-Mediated Inhibition of Neuroinflammation in Hypoxic Ischemic Brain Injury. Neural Regen. Res..

[71] Wang L., Yao C., Chen J., Ge Y., Wang C., Wang Y., Wang F., Sun Y., Dai M., Lin Y. (2022). Γδ T Cell in Cerebral Ischemic Stroke: Characteristic, Immunity-Inflammatory Role, and Therapy. Front. Neurol..

[72] Zou Y.X., Zhang X.H., Su F.Y., Liu X. (2012). Importance of Riboflavin Kinase in the Pathogenesis of Stroke. CNS Neurosci. Ther..

[73] Zhang M., Chen H., Zhang W., Liu Y., Ding L., Gong J., Ma R., Zheng S., Zhang Y. (2023). Biomimetic Remodeling of Microglial Riboflavin Metabolism Ameliorates Cognitive Impairment by Modulating Neuroinflammation. Adv. Sci..

[74] Nasoohi S., Ismael S., Ishrat T. (2018). Thioredoxin-Interacting Protein (TXNIP) in Cerebrovascular and Neurodegenerative Diseases: Regulation and Implication. Mol. Neurobiol..

[75] Liu Z., Cheng P., Feng T., Xie Z., Yang M., Chen Z., Hu S., Han D., Chen W. (2023). Nrf2/HO-1 Blocks TXNIP/NLRP3 Interaction via Elimination of ROS in Oxygen-Glucose Deprivation-Induced Neuronal Necroptosis. Brain Res..

[76] Mao L.L., Hao D.L., Mao X.W., Xu Y.F., Huang T.T., Wu B.N., Wang L.H. (2015). Neuroprotective Effects of Bisperoxovanadium on Cerebral Ischemia by Inflammation Inhibition. Neurosci. Lett..

[77] Deng Y., Huang P., Zhang F., Chen T. (2022). Association of MicroRNAs With Risk of Stroke: A Meta-Analysis. Front. Neurol..

[78] Eyileten C., Wicik Z., De Rosa S., Mirowska-Guzel D., Soplinska A., Indolfi C., Jastrzebska-Kurkowska I., Czlonkowska A., Postula M. (2018). Cells MicroRNAs as Diagnostic and Prognostic Biomarkers in Ischemic Stroke-A Comprehensive Review and Bioinformatic Analysis. Cells.

[79] Otero-Ortega L., Alonso-López E., Pérez-Mato M., Laso-García F., Gómez-De Frutos M.C., Diekhorst L., García-Bermejo M.L., Conde-Moreno E., Fuentes B., de Leciñana M.A. (2021). Circulating Extracellular Vesicle Proteins and MicroRNA Profiles in Subcortical and Cortical-Subcortical Ischaemic Stroke. Biomedicines.

[80] Song P., Sun H., Chen H., Wang Y., Zhang Q. (2020). Decreased Serum Exosomal MiR-152-3p Contributes to the Progression of Acute Ischemic Stroke. Clin. Lab..

[81] Wang W., Sun G., Zhang L., Shi L., Zeng Y. (2014). Circulating MicroRNAs as Novel Potential Biomarkers for Early Diagnosis of Acute Stroke in Humans. J. Stroke Cerebrovasc. Dis..

[82] Jiang S., Wu J., Geng Y., Zhang Y., Wang Y., Wu J., Lu C., Luo G., Zan J., Zhang Y. (2022). Identification of Differentially Expressed MicroRNAs Associated with Ischemic Stroke by Integrated Bioinformatics Approaches. Int. J. Genom..

[83] van Kralingen J.C., McFall A., Ord E.N.J., Coyle T.F., Bissett M., McClure J.D., McCabe C., Macrae I.M., Dawson J., Work L.M. (2019). Altered Extracellular Vesicle MicroRNA Expression in Ischemic Stroke and Small Vessel Disease. Transl. Stroke Res..

[84] Zhou X., Xu C., Chao D., Chen Z., Li S., Shi M., Pei Y., Dai Y., Ji J., Ji Y. (2022). Acute Cerebral Ischemia Increases a Set of Brain-Specific MiRNAs in Serum Small Extracellular Vesicles. Front. Mol. Neurosci..

[85] Eyileten C., Jakubik D., Shahzadi A., Gasecka A., van der Pol E., De Rosa S., Siwik D., Gajewska M., Mirowska-Guzel D., Kurkowska-Jastrzebska I. (2022). Diagnostic Performance of Circulating MiRNAs and Extracellular Vesicles in Acute Ischemic Stroke. Int. J. Mol. Sci..

[86] Gendosz de Carrillo D., Kocikowska O., Rak M., Krzan A., Student S., Jędrzejowska-Szypułka H., Pawletko K., Lasek-Bal A. (2024). The Relevance of Reperfusion Stroke Therapy for MiR-9-3p and MiR-9-5p Expression in Acute Stroke—A Preliminary Study. Int. J. Mol. Sci..

[87] Curtaz C.J., Reifschläger L., Strähle L., Feldheim J., Feldheim J.J., Schmitt C., Kiesel M., Herbert S.L., Wöckel A., Meybohm P. (2022). Analysis of MicroRNAs in Exosomes of Breast Cancer Patients in Search of Molecular Prognostic Factors in Brain Metastases. Int. J. Mol. Sci..

[88] Wu J., Du K., Lu X. (2015). Elevated Expressions of Serum MiR-15a, MiR-16, and MiR-17-5p Are Associated with Acute Ischemic Stroke. Int. J. Clin. Exp. Med..

[89] Zhao H., Wang J., Gao L., Wang R., Liu X., Gao Z., Tao Z., Xu C., Song J., Ji X. (2013). MiRNA-424 Protects against Permanent Focal Cerebral Ischemia Injury in Mice Involving Suppressing Microglia Activation. Stroke.

[90] Guo C., Yao Y., Li Q., Gao Y., Cao H. (2022). Expression and Clinical Value of MiR-185 and MiR-424 in Patients with Acute Ischemic Stroke. Int. J. Gen. Med..

[91] Tian C., Li Z., Yang Z., Huang Q., Liu J., Hong B. (2016). Plasma MicroRNA-16 Is a Biomarker for Diagnosis, Stratification, and Prognosis of Hyperacute Cerebral Infarction. PLoS ONE.

[92] Wang Y., Su X., Leung G.H.D., Ren B., Zhang Q., Xiong Z., Zhou J., Yang L., Lu G., Chan W.Y. (2023). Circulating MicroRNAs as Diagnostic Biomarkers for Ischemic Stroke: Evidence from Comprehensive Analysis and Real-World Validation. Int. J. Med. Sci..

[93] Huang H., Wei G., Wang C., Lu Y., Liu C., Wang R., Shi X., Yang J., Wei Y. (2019). A Functional Polymorphism in the Promoter of MiR-17-92 Cluster Is Associated with Decreased Risk of Ischemic Stroke. BMC Med. Genom..

[94] Ma Q., Li G., Tao Z., Wang J., Wang R., Liu P., Luo Y., Zhao H. (2019). Blood MicroRNA-93 as an Indicator for Diagnosis and Prediction of Functional Recovery of Acute Stroke Patients. J. Clin. Neurosci..

[95] Wang R., Bao H., Zhang S., Li R., Chen L., Zhu Y. (2018). MiR-186-5p Promotes Apoptosis by Targeting IGF-1 in SH-SY5Y OGD/R Model. Int. J. Biol. Sci..

[96] You J., Qian F., Huang Y., Guo Y., Lv Y., Yang Y., Lu X., Guo T., Wang J., Gu B. (2022). LncRNA WT1-AS Attenuates Hypoxia/Ischemia-Induced Neuronal Injury During Cerebral Ischemic Stroke via MiR-186-5p/XIAP Axis. Open Med..

[97] Tiedt S., Prestel M., Malik R., Schieferdecker N., Duering M., Kautzky V., Stoycheva I., Böck J., Northoff B.H., Klein M. (2017). RNA-Seq Identifies Circulating MIR-125a-5p, MIR-125b-5p, and MIR-143-3p as Potential Biomarkers for Acute Ischemic Stroke. Circ. Res..

[98] Chen Z., Wang K., Huang J., Zheng G., Lv Y., Luo N., Liang M., Huang L. (2018). Upregulated Serum MiR-146b Serves as a Biomarker for Acute Ischemic Stroke. Cell. Physiol. Biochem..

[99] Salman A.T., Shaker O., Elshaer S.S., Elshafei A. (2022). The Expression Profiling of Serum MiR-92a, MiR-134 and MiR-375 in Acute Ischemic Stroke. Future Sci. OA.

[100] Aldous E.K., Toor S.M., Parray A., Al-Sarraj Y., Diboun I., Abdelalim E.M., Arredouani A., El-Agnaf O., Thornalley P.J., Akhtar N. (2022). Identification of Novel Circulating MiRNAs in Patients with Acute Ischemic Stroke. Int. J. Mol. Sci..

[101] Ma Q., Zhao H., Tao Z., Wang R., Liu P., Han Z., Ma S., Luo Y., Jia J. (2016). MicroRNA-181c Exacerbates Brain Injury in Acute Ischemic Stroke. Aging Dis..

[102] Jin F., Xing J. (2017). Circulating Pro-Angiogenic and Anti-Angiogenic MicroRNA Expressions in Patients with Acute Ischemic Stroke and Their Association with Disease Severity. Neurol. Sci..

[103] Rahmati M., Ferns G.A., Mobarra N. (2021). The Lower Expression of Circulating MiR-210 and Elevated Serum Levels of HIF-1α in Ischemic Stroke; Possible Markers for Diagnosis and Disease Prediction. J. Clin. Lab. Anal..

[104] Zeng L.L., He X.S., Liu J.R., Zheng C.B., Wang Y.T., Yang G.Y. (2016). Lentivirus-Mediated Overexpression of MicroRNA-210 Improves Long-Term Outcomes After Focal Cerebral Ischemia in Mice. CNS Neurosci. Ther..

[105] Zeng L., Liu J., Wang Y., Wang L., Weng S., Tang Y., Zheng C., Cheng Q., Chen S., Yang G.Y. (2011). MicroRNA-210 as a Novel Blood Biomarker in Acute Cerebral Ischemia. Front. Biosci..

[106] Kong Y., Li S., Cheng X., Ren H., Zhang B., Ma H., Li M., Zhang X.A. (2020). Brain Ischemia Significantly Alters MicroRNA Expression in Human Peripheral Blood Natural Killer Cells. Front. Immunol..

[107] van Wijk N., Zohar K., Linial M. (2022). Challenging Cellular Homeostasis: Spatial and Temporal Regulation of MiRNAs. Int. J. Mol. Sci..

[108] Burek M., König A., Lang M., Fiedler J., Oerter S., Roewer N., Bohnert M., Thal S.C., Blecharz-Lang K.G., Woitzik J. (2019). Hypoxia-Induced MicroRNA-212/132 Alter Blood-Brain Barrier Integrity Through Inhibition of Tight Junction-Associated Proteins in Human and Mouse Brain Microvascular Endothelial Cells. Transl. Stroke Res..

[109] Teles R.H.G., Engelmayr D., Meybohm P., Burek M. (2024). Isolation of Extracellular Vesicles Using Formulas to Adapt Centrifugation to Different Centrifuges. Methods Mol. Biol..

[110] Exosomal miRNAs A., Dugandži A., Maria Ciaccio A., Tuttolomondo A. (2023). Exosomal MiRNAs as Biomarkers of Ischemic Stroke. Brain Sci..

[111] Eskandar S. Exploring Common Distance Measures for Machine Learning and Data Science: A Comparative | Medium [Internet]. https://medium.com/@eskandar.sahel/exploring-common-distance-measures-for-machine-learning-and-data-science-a-comparative-analysis-ea0216c93ba3.

[112] Hirschberger S., Hübner M., Strauß G., Effinger D., Bauer M., Weis S., Giamarellos-Bourboulis E.J., Kreth S. (2019). Identification of Suitable Controls for MiRNA Quantification in T-Cells and Whole Blood Cells in Sepsis. Sci. Rep..

[113] Mi H., Muruganujan A., Thomas P.D. (2013). PANTHER in 2013: Modeling the Evolution of Gene Function, and Other Gene Attributes, in the Context of Phylogenetic Trees. Nucleic Acids Res..

[114] Thomas P.D., Ebert D., Muruganujan A., Mushayahama T., Albou L., Mi H. (2022). PANTHER: Making Genome-scale Phylogenetics Accessible to All. Protein Sci..

[115] Grissa D., Junge A., Oprea T.I., Jensen L.J. (2022). Diseases 2.0: A Weekly Updated Database of Disease-Gene Associations from Text Mining and Data Integration. Database.

[116] Agrawal A., Balcı H., Hanspers K., Coort S.L., Martens M., Slenter D.N., Ehrhart F., Digles D., Waagmeester A., Wassink I. (2024). WikiPathways 2024: Next Generation Pathway Database. Nucleic Acids Res..

[117] Kanehisa M., Sato Y. (2020). KEGG Mapper for Inferring Cellular Functions from Protein Sequences. Protein Sci..

[118] Kanehisa M., Sato Y., Kawashima M. (2022). KEGG Mapping Tools for Uncovering Hidden Features in Biological Data. Protein Sci..

[119] Nesterova A.P., Klimov E.A., Zharkova M., Sozin S., Sobolev V., Ivanikova N.V., Shkrob M., Yuryev A. (2019). Disease Pathways: An Atlas of Human Disease Signaling Pathways. Disease Pathways: An Atlas of Human Disease Signaling Pathways.

[120] Wiebe D.S., Omelyanchuk N.A., Mukhin A.M., Grosse I., Lashin S.A., Zemlyanskaya E.V., Mironova V.V. (2020). Fold-Change-Specific Enrichment Analysis (FSEA): Quantification of Transcriptional Response Magnitude for Functional Gene Groups. Genes.

