# Supplementary material for: Reduced Expression of Selected Exosomal MicroRNAs Is Associated with Poor Outcomes in Patients with Acute Stroke Receiving Reperfusion Therapy—Preliminary Study
Source: Int J Mol Sci. 2025 Sep 29;26(19):9533. doi: 10.3390/ijms26199533 (PMC12524926; doi:10.3390/ijms26199533)
Supplement: Supplementary file 1 [file ijms-26-09533-s001.zip › ijms-3791834-supplementary.pdf]

## Supplementary material

*Article*

# Reduced expression of selected exosomal microRNAs is associated with poor outcomes in patients with acute stroke receiving reperfusion therapy-preliminary study.

**Daria Gendosz de Carrillo [1,2,*], Olga Kocikowska [1,3] , Aleksandra Krzan [4,5], Sebastian Student [3,6] , Małgorzata Rak [1], Magdalena Nowak [1] , Junqiao Mi[7], Małgorzata Burek[7], Anetta Lasek-Bal [4,5], Halina Jędrzejowska-Szypułka [1]**

[1] Department of Physiology, Faculty of Medicine, Medical University of Silesia in Katowice, 40-752 Katowice, Poland;
[2] Department of Histology and Cell Pathology, Faculty of Medical Sciences in Zabrze, Medical University of Silesia in Katowice, 40-752 Katowice, Poland
[3] Department of Engineering and Systems Biology, Faculty of Automatic Control, Electronics and Computer Science, Silesian University of Technology, 44-100 Gliwice, Poland;
[4] Department of Neurology, School of Health Sciences, Medical University of Silesia in Katowice, 40-752 Katowice, Poland;
[5] Department of Neurology, Upper-Silesian Medical Center of the Silesian Medical University, 40-752 Katowice, Poland
[6] Biotechnology Centre, Silesian University of Technology, 44-100 Gliwice, Poland
[7] University Hospital Wuerzburg, Department of Anaesthesiology, Intensive Care, Emergency and Pain Medicine, Wuerzburg, Germany
*correspondence dgendosz@sum.edu.pl

ORCID 0000-0001-5090-8365

Academic Editors: Kyriacos N. Felekkis and Christos Papaneophytou

Received: 16 July 2025
Revised: 10 September 2025
Accepted: 12 September 2025
Published: 26 September 2025

**Citation:** Gendosz de Carrillo, D.; Kocikowska, O.; Krzan, A.; Student, S.; Rak, M.; Nowak-Andraka, M.; Mi, J.; Burek, M.; Lasek-Bal, A.; Jędrzejowska-Szypułka, H. Reduced Expression of Selected Exosomal MicroRNAs Is Associated with Poor Outcomes in Patients with Acute Stroke Receiving Reperfusion Therapy—Preliminary Study. *Int. J. Mol. Sci.* **2025**, *26*, x. https://doi.org/10.3390/xxxxx

**Copyright:** © 2025 by the authors. Licensee MDPI, Basel, Switzerland. This article is an open access article distributed under the terms and conditions of the Creative Commons Attribution (CC BY) license (https://creativecommons.org/licenses/by/4.0/).

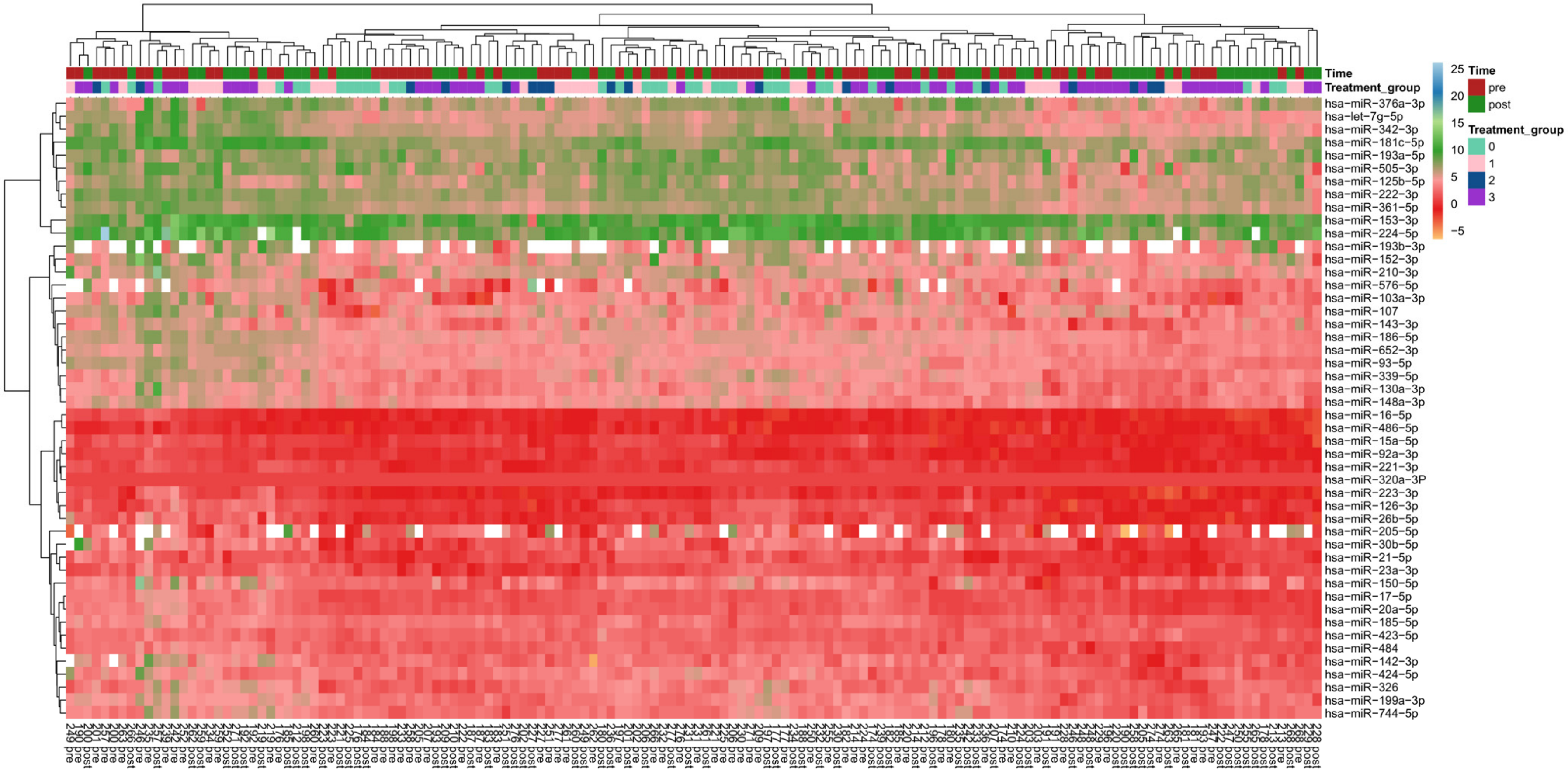


Figure S1. Represents normalized global expression of analyzed miRNA heat map in patients pre and post stroke treatment with canberra clusterization, where annotations: red–day 1; green–day 10 post-treatment; aquamarine–patients treated with aspirin; pink–patients treated with rt-PA; blue–patients treated with MT; purple–patients treated with rt-PA/MT [120].

A rt-PA/MT

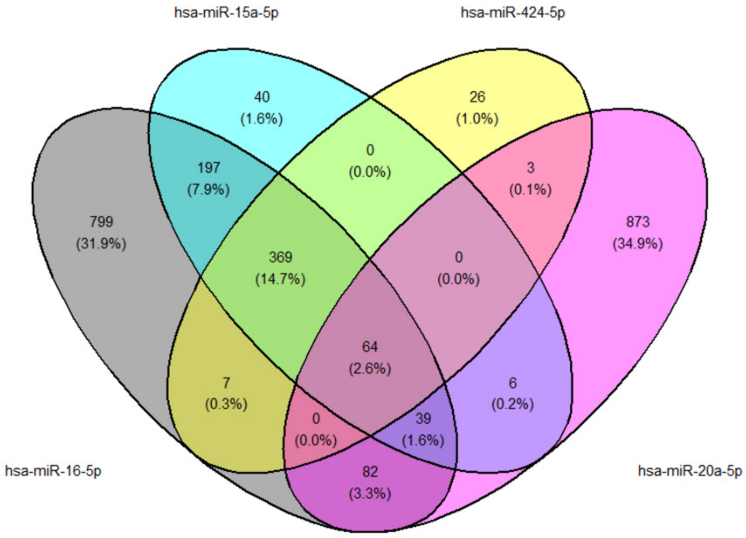


B 10-day mRS

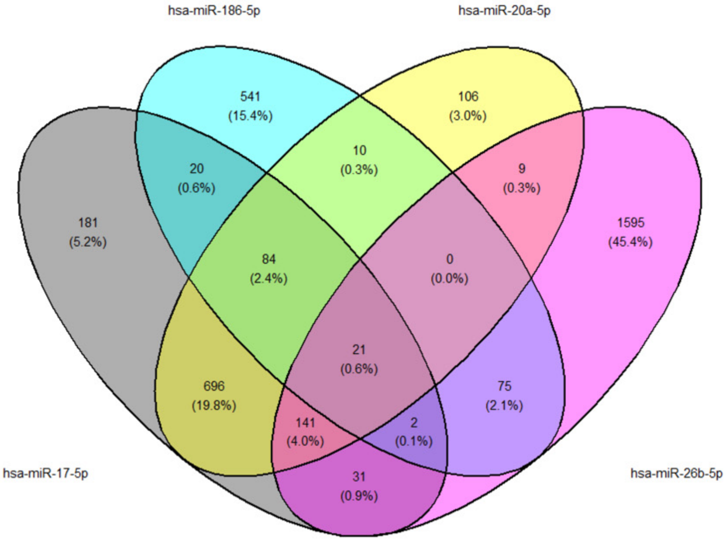


C 90-day mRS

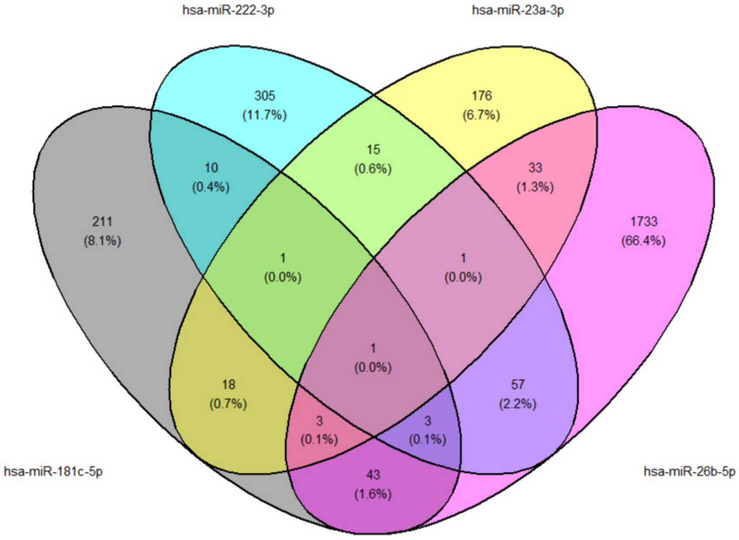

Figure S2. A) The Venn diagram displays the ETs of hsa-miR-15a-5p, hsa-miR-16-5p, hsa-miR-20a-5p, and hsa-miR-424-5p, which showed the highest fold change in the rt-PA/MT group. The values in overlapping circles represent the estimated target genes for each individual DEmiRNA or shared between two, three, or all four of them. B) The Venn diagram displays the ETs of hsa-miR-17-5p, hsa-miR-20a-5p, hsa-miR-26b-5p and hsa-miR-186-5p. The values in overlapping circles represent the estimated target genes for each individual DEmiRNA or shared between two, three, or all four of them. C) The Venn diagram displays the ETs of hsa-miR-23a-3p, hsa-miR-26b-5p, hsa-miR- 181c-5p and hsa-miR-222-3p. The values in overlapping circles represent the estimated target genes for each individual DEmiRNA or shared between two, three, or all four of them.

Table S1. Pathway enrichment. Figure 5A detailed data.

| Enrichment FDR | nGenes | Pathway Genes | Fold Enrichment | Pathway | Genes |
|---|---|---|---|---|---|
| 0.001361119 | 5 | 5 | 5.232334782 | P02721 ATP synthesis | CYCS MT-ATP6 ATP5F1A ATP5F1B ATP5F1C |
| 0.004778507 | 10 | 19 | 2.753860411 | P00025 Hedgehog signaling pathway | BTRC FBXW11 UBR5 CSNK1A1 CSNK1E GLI3 GSK3B PRKAR1A PRKAR2A PTCH1 |
| 0.003048741 | 11 | 21 | 2.74074679 | P04397 p53 pathway by glucose deprivation | RHEB RPS6KB1 STK11 TP53 TSC1 TSC2 TP63 EIF4EBP1 AKT1 PRKAA1 PRKAB2 |
| 0.006344333 | 12 | 26 | 2.414923745 | P00030 Hypoxia response via HIF activation | TXN EGLN2 EGLN3 TXN2 AKT1 MTOR HIF1A PIK3CA PIK3CD PIK3R1 PIK3R2 PTEN |
| 0.000284793 | 21 | 46 | 2.388674574 | P00046 Oxidative stress response | STYX TXN DUSP18 EEF2K DUSP10 DUSP2 DUSP4 DUSP5 DUSP7 ELK1 JUN MAP2K3 MAP2K4 MAPK14 MAPK8 MAPK9 |

| | | | | | |
|---|---|---|---|---|---|
| | | | | | MEF2C MKNK2 MYC ATF2 BCL2 |
| 1.07E-05 | 31 | 69 | 2.350759105 | P04393 Ras Pathway | RPS6KA3 SRF STAT3 ELK1 ETS1 AKT1 GRB2 GSK3B JUN KRAS RHOA RHOB RHOC MAP2K1 MAP2K3 MAP2K4 MAP3K4 MAPK1 MAPK14 MAPK8 MAPK9 MAPKAPK2 ATF2 NRAS PAK2 PDPK1 PIK3CA PIK3CD RAF1 RALA RALGDS |
| 9.91E-09 | 55 | 125 | 2.302227304 | P00047 PDGF signaling pathway | RPS6KA3 RPS6KA4 RPS6KA5 RPS6KB1 SRF STAT2 STAT3 STAT5A VAV2 VAV3 GAB2 NIN ARHGAP12 SHC3 CHUK ARHGAP10 ARHGAP42 SHC2 ELF4 ELK1 ELK4 ETS1 FOS GAB1 GABPA GRB2 GSK3B ELP1 ITPR1 ITPR3 JAK1 JAK2 JUN ARHGAP1 ARHGAP5 MAP2K1 MAP3K2 MAP3K4 MAPK1 MAPK6 MAPK8 MAPKAPK2 MKNK2 MYC NRAS PDGFB PDPK1 |

| | | | | | |
|---|---|---|---|---|---|
| | | | | | PIK3CA PIK3CD PIK3R1 PIK3R2 PLCG1 PRKCA RAF1 RASA1 |
| 3.17E-06 | 44 | 108 | 2.131691948 | P00006 Apoptosis signaling pathway | TMBIM6 TNFRSF10A TNFRSF10B FAS TNFSF10 FASLG TP53 CHUK CYCS DIABLO EIF2S1 FOS AKT1 BCL2L2 HSPA5 HSPA8 IGF2R XIAP JUN MAP2K3 MAP2K4 MAP3K5 MAP4K2 MAPK1 MAPK8 MAPK9 MCL1 NFKB1 NFKBIA ATF2 ATF3 ATF6 ATF7 PIK3CA PIK3CD PRKCA PRKCB PRKCD BAG4 BAK1 BCL2 BCL2L11 REL RELA |
| 7.18E-08 | 62 | 156 | 2.07951767 | P00005 Angiogenesis | STAT3 TCF7L2 VEGFA WNT2B WNT5A RBPJL WNT5B FRS2 CRK CRKL DLK1 SHC2 PLA2G4D DVL3 EFNB1 EFNB2 EPHB2 ETS1 F2R F3 FGFR1 FOS AKT1 GRB2 GSK3B HIF1A RBPJ APC BIRC5 JAG2 JAK1 JUN KDR RHOA RHOB RHOC ARHGAP1 MAP2K1 MAP2K4 |

| | | | | | |
|---|---|---|---|---|---|
| | | | | | MAPK1 MAPK14 MAPK8 MAPKAPK2 NOTCH2 NRAS PAK2 PDGFB PIK3CA PIK3CD PIK3R1 PIK3R2 AXIN1 PLCG1 PRKCA PRKCB PRKCD PRKCI PRKD3 PTPN6 PXN RAF1 RASA1 |
| 0.001084517 | 24 | 61 | 2.058623521 | P00056 VEGF signaling pathway | VEGFA SHC2 ETS1 AKT1 HIF1A KDR ARHGAP1 MAP2K1 MAPK1 MAPK14 MAPKAPK2 NRAS PIK3CA PIK3CD PIK3R1 PIK3R2 PLCG1 PRKCA PRKCB PRKCD PRKCI PRKD3 PXN RAF1 |

Table S2: Pathway enrichment. Figure 5B detailed data.

| Enrichment FDR | nGenes | Pathway Genes | Fold Enrichment | Pathway | Genes |
|---|---|---|---|---|---|
| 0.001086262 | 6 | 6 | 5.232334782 | DOID:0081062 diabetes | TGFB1 VIM HTRA1 EGFR CDH1 IGF1R |
| 0.017873126 | 4 | 4 | 5.232334782 | DOID:11695 portal vein thrombosis | F2 MTHFR SERPINE1 IGF1R |
| 0.038927671 | 5 | 7 | 3.737381987 | DOID:870 neuropathy | SLC25A46 MED24 YARS1 NEMF CTSS |
| 0.038927671 | 5 | 7 | 3.737381987 | DOID:0050548 hereditary sensory neuropathy | SPTLC1 CCT4 WNK1 DNMT1 KIF1A |

| | | | | | |
|---|---|---|---|---|---|
| 0.009489937 | 7 | 10 | 3.662634347 | DOID:4752 multiple system atrophy | SLC1A1 FAS IGF2 AMBRA1 SLC6A6 SNCA COQ2 |
| 0.022094792 | 8 | 14 | 2.989905589 | DOID:0060307 autosomal dominant intellectual developmental disorder | ZBTB18 AUTS2 TRIO SET CAMK2G ATP2B1 AP2M1 MED13 |
| 0.006332782 | 12 | 23 | 2.729913799 | DOID:1561 cognitive disorder | DBN1 EPHB2 APP FGF2 LAMP2 COMT MMP3 IRAK1 PPARG APBB2 BDNF DMD |
| 0.005664544 | 15 | 32 | 2.452656929 | DOID:8725 vascular dementia | SNCG ATP5F1A SNCA SREBF2 EIF4E FGFR1 BDNF GSK3B MTOR RPS6 TP53 MMP2 IGF1R PON2 GCLM |
| 0.000630099 | 21 | 45 | 2.441756231 | DOID:2476 hereditary spastic paraplegia | SEC23IP ATP13A3 KIF5A RTN4 KIF5B ATP5MC2 AFG3L2 ATL2 RTN2 DDHD2 ARL6IP1 ATL3 ATP5MC3 PNPLA6 RTN3 AP5Z1 L2HGDH ALDH18A1 BSCL2 KIF1A HSPD1 |
| 0.000998134 | 20 | 43 | 2.433644084 | DOID:14250 Down syndrome | TUBA1A PCNT DYRK1A HSPD1 GDI2 CTSS DBN1 IGFBP3 ACTR1A ERCC3 ERCC2 RCAN1 RCAN3 DYRK3 DYRK2 DOP1B SYNJ1 RPLP0 CBS MTHFR |

Table S3. Pathway enrichment. Figure 5C detailed data.

| Enrichment FDR | nGenes | Pathway Genes | Fold Enrichment | Pathway | Genes |
|---|---|---|---|---|---|
| 1.84E-13 | 36 | 54 | 3.488223188 | HALLMARK TGF BETA SIGNALING | TGFBR1 SMAD7 TGFB1 SMURF2 BMPR2 SKIL SKI ACVR1 PMEPA1 NCOR2 SERPINE1 SMAD1 SMAD6 PPP1R15A TGIF1 FURIN SMAD3 FKBP1A MAP3K7 BMPR1A HIPK2 KLF10 BMP2 APC PPM1A XIAP CDH1 SLC20A1 CDK9 ARID4B PPP1CA SPTBN1 FNTA HDAC1 LTBP2 RHOA |
| 1.47E-18 | 65 | 113 | 3.009750096 | HALLMARK UNFOLDED PROTEIN RESPONSE | HERPUD1 HSP90B1 XBP1 HSPA5 ATF3 EXOSC1 ASNS SRPRB DNAJB9 TSPYL2 CALR KHSRP NOP14 ATF6 RRP9 CNOT4 DCP2 PDIA6 PREB SERP1 NFYB DCTN1 SLC7A5 EDEM1 EDC4 MTHFD2 SRPRA NOLC1 ERN1 FKBP14 DKC1 |

| | | | | | |
|---|---|---|---|---|---|
| | | | | | EIF4EBP1 EIF2S1 TATDN2 XPOT CHAC1 STC2 NABP1 EIF4A2 EIF4E GEMIN4 ALDH18A1 DDIT4 HSPA9 SEC11A POP4 CEBPG CEBPB CNOT2 SPCS3 RPS14 NPM1 CNOT6 PSAT1 EIF4G1 YWHAZ KIF5B SPCS1 DNAJA4 VEGFA TTC37 TUBB2A EXOSC10 EEF2 PAIP1 |
| 2.14E-19 | 96 | 200 | 2.511520695 | HALLMARK MTORC1 SIGNALING | DDIT4 CALR PGK1 SLC7A5 SLC1A5 M6PR TFRC TMEM97 IFRD1 PSAT1 CORO1A MTHFD2 VLDLR WARS1 SCD ACTR2 SERPINH1 PSPH NAMPT CDKN1A BHLHE40 HSPA9 HSPA5 EGLN3 PNP XBP1 DDX39A ACLY SLC7A11 SQSTM1 PSMC2 SERP1 HMGCS1 TPI1 ELOVL6 ASNS PSMA4 PPA1 AURKA HMGCR GAPDH IMMT YKT6 INSIG1 IGFBP5 ENO1 SHMT2 TXNRD1 RAB1A EBP PNO1 LDLR SLC2A3 ELOVL5 EDEM1 TES SLC6A6 CCNF BTG2 NMT1 FDXR RRM2 DHCR24 PSME3 POLR3G PSMD12 NIBAN1 TBK1 SEC11A BCAT1 PSMB5 PSMD13 PLK1 GLRX COPS5 ETF1 GSK3B NUP205 RRP9 MLLT11 TCEA1 MAP2K3 HSPD1 PPP1R15A UNG HSP90B1 CDC25A PDAP1 BUB1 ARPC5L ATP2A2 GGA2 SKAP2 STIP1 ABCF2 CCT6A |
| 2.19E-18 | 94 | 200 | 2.459197347 | HALLMARK TNFA SIGNALING VIA NFKB | ATF3 NFKBIA PTGS2 TNFAIP2 KLF6 PLAUR ICAM1 JUN PPP1R15A SOD2 BTG2 MAP3K8 F3 KDM6B NFKB1 FOSL1 NR4A1 RCAN1 DUSP2 IER2 REL TNFSF9 CXCL10 BHLHE40 EGR2 SOCS3 PTGER4 DUSP5 SERPINE1 TIPARP RELA LITAF CD44 MYC NAMPT PNRC1 CD69 EFNA1 PFKFB3 YRDC SQSTM1 MCL1 VEGFA MAP2K3 CDKN1A TANK TUBB2A IRF1 FOS RHOB ZBTB10 PLPP3 KLF4 SAT1 |

| | | | | | |
|---|---|---|---|---|---|
| | | | | | CSF1 GPR183 PMEPA1 KLF10 LAMB3 CEBPB TRIP10 F2RL1 KLF9 LDLR TGIF1 RNF19B B4GALT1 DNAJB4 SNN STAT5A DENND5A CCND1 B4GALT5 SIK1 NFE2L2 PER1 NFAT5 ATP2B1 IL12B IL6ST ABCA1 HES1 SLC2A3 SMAD3 IFIH1 PANX1 FJX1 EIF1 BMP2 DUSP4 PDLIM5 GFPT2 KLF2 MXD1 |
| 0.002111894 | 14 | 32 | 2.289146467 | HALLMARK NOTCH SIGNALING | NOTCH2 HES1 CCND1 FZD7 WNT5A SAP30 PPARD KAT2A HEYL RBX1 TCF7L2 ARRB1 PRKCA FBXW11 |
| 2.00E-11 | 69 | 161 | 2.242429192 | HALLMARK APOPTOSIS | PMAIP1 JUN BCL2L11 MCL1 DIABLO BID CDKN1A CDKN1B TNFSF10 SQSTM1 FASLG CD44 FAS CCND1 F2R SATB1 TNFRSF12A RHOB GPX3 CCND2 XIAP IRF1 ADD1 TIMP2 BTG2 TIMP3 IGF2R AIFM3 PPP3R1 HGF CLU ATF3 SMAD7 HMOX1 MMP2 VDAC2 APP BRCA1 SOD2 BMF EREG RELA WEE1 CD69 PEA15 CASP2 BMP2 HMGB2 TGFBR3 EBP TXNIP ANKH RHOT2 CYLD MGMT PPT1 F2 RNASEL CAV1 DNM1L TOP2A SLC20A1 SAT1 PDCD4 BCL2L2 DNAJA1 DAP3 FDXR ETF1 |
| 7.22E-11 | 79 | 200 | 2.066772239 | HALLMARK P53 PATHWAY | CDKN1A BTG2 MDM2 FAS TOB1 CDKN2B ZMAT3 DDIT4 ATF3 SESN1 FDXR SAT1 CDKN2A AEN PPM1D FOXO3 TXNIP TP53 FOS FBXW7 AK1 BAK1 CCND2 RALGDS SERPINB5 KLF4 TNFSF9 RB1 PPP1R15A RAB40C TGFB1 TP63 HSPA4L TRAF4 TAX1BP3 PLXNB2 HEXIM1 NOL8 SP1 JAG2 CCP110 MXD1 RAD51C ITGB4 VAMP8 CTSD ELP1 POM121 EPHA2 JUN SLC7A11 SERTAD3 IRAK1 ABCC5 RPL36 RNF19B CDK5R1 STEAP3 GM2A RPS12 TM4SF1 MKNK2 RACK1 TGFA ISCU LDHB APP F2R ACVR1B |

| | | | | | |
|---|---|---|---|---|---|
| | | | | | BMP2 TM7SF3 ZBTB16 TSPYL2 SLC3A2 GLS2 CCND3 DNTTIP2 SEC61A1 HMOX1 |
| 0.006382377 | 14 | 36 | 2.03479686 | HALLMARK HEDGEHOG SIGNALING | PTCH1 NRP1 AMOT CDK6 NRP2 TLE3 L1CAM RASA1 MYH9 VEGFA CELSR1 CDK5R1 VLDLR DPYSL2 |
| 0.001923812 | 19 | 49 | 2.028864507 | HALLMARK REACTIVE OXYGEN SPECIES PATHWAY | PRDX2 TXNRD1 GCLM TXN SOD2 PRDX4 PDLIM1 LAMTOR5 PFKP GPX3 SCAF4 FTL ATOX1 STK25 EGLN2 ERCC2 PTPA GLRX PRNP |

Table S4. Pathway enrichment. Figure 5D detailed data.

| Enrichment FDR | nGenes | Pathway Genes | Fold Enrichment | Pathway | Genes |
|---|---|---|---|---|---|
| 0.00409044 | 4 | 4 | 5.232334782 | WP5189 Copper metabolism | ATP7B SLC11A2 ATP7A SLC31A1 |
| 0.000236001 | 6 | 6 | 5.232334782 | WP5241 Mitochondrial beta oxidation | DECR1 ECI1 ACAA2 ACADSB HSD17B10 ECHS1 |
| 5.21E-06 | 11 | 13 | 4.4273602 | WP4919 Neuroinflammation | NFKBIA RELA FOS CHUK MAPK14 MTOR MAPK8 MT-CO1 MT-CO2 ASCC1 JUN |
| 6.12E-07 | 19 | 29 | 3.428081409 | WP3972 PDGFR-beta pathway | STAT3 FOS JAK1 JAK2 GRB2 ELK1 PLCG1 MAPK8 SRF PRKCA PRKCB PIK3R1 RAF1 PIK3CA STAT5A MAP2K4 RASA1 MAP2K1 JUN |
| 1.20E-06 | 19 | 30 | 3.313812028 | WP2870 Extracellular vesicle-mediated signaling in recipient cells | HGF TGFBR1 TGFBR2 NRAS AXIN1 TGFBR3 TGFB1 EGFR WNT3A SMAD2 WNT5A SMAD3 SMAD4 TGFA RAF1 APC MTOR AKT1 KRAS |
| 0.000139588 | 15 | 27 | 2.906852656 | WP5231 Hippocampal synaptogenesis and neurogenesis | NRXN3 CREB1 BCL2 CCND2 MAPK14 SYT2 PRKCA CAMK4 CAMK2B NRXN1 MAPK1 NCAM1 RPS6KA5 CAMKK2 BDNF |
| 3.90E-38 | 204 | 431 | 2.47655753 | WP3888 VEGFA-VEGFR2 signaling pathway | CLTC TBCA FOXO3 FOXO1 GAB1 PNP LUC7L FLNB PXN PSMD11 GIPC1 TRAF3IP2 ARPC5L CALR PSMD4 CALU ELOC ELOA MYH9 KDR RELA MYO1C CAMKK2 GJA1 DNAJA1 MAPK9 MAP2K1 MAP2K3 CREB1 FHOD1 HSP90AA1 SND1 CAPZB MYO6 TMOD3 ASCC3 EIF2A TXNIP CNP |

| | | | | | |
|---|---|---|---|---|---|
| | | | | | ETS1 GAPDH ATF2 MTOR PTGS2 ITGB1 NFKB1 NFKBIA NAP1L1 PBXIP1 CRK CXCL8 ELK1 AFDN SRF CAV1 PTMA PTPN1 RAF1 COPG1 CBL MMP2 SRPK1 STIP1 PTPN9 NCL PDIA6 IGFBP3 F3 HTRA1 PTPN6 MEF2C VPS39 SHC2 INPP4B PTPRJ MOV10 PRRC2C ZC3H15 MAP3K5 ATP6V1E1 PRKAA1 STAT3 ICAM1 BMP2 EPHB2 NRP2 SOD2 IQGAP1 SLC7A1 CACNA2D1 HMGB1 MAP2K4 SET PLAUR PRKCD PRKCI NR4A1 RPL13A PLCG1 SLC8A1 PRKCA PRKCB PFN1 CCT7 ERN1 PRKG1 PGK1 MAPK1 VAV2 BSG AKT1 PGD CYCS HERPUD1 VEGFA HDAC1 BIRC5 MAPK8 VCL SYNJ1 EZR RAB11A PPP1CA ADAM10 SMARCA2 EEA1 CHAC1 CCND1 BCL2 FAS ADAMTS1 P4HB FXR2 EIF3H DNAJB9 EIF3F RPL5 FJX1 FBXW11 TUBA1C MAPKAPK5 MICAL2 RICTOR MDM2 RPL7 RPL18A PIK3R1 PIK3R2 UBAP2L PIK3CA PDPK1 ARF6 RHOA RHOC AKT1S1 RPL27 PRDX2 PAK2 PDE4DIP ABCF2 AP2A1 C15orf39 RCAN1 SPIRE1 QKI TFAM FAF1 ACTG1 GRSF1 RACK1 ACOT9 GSK3B LRRFIP2 EPHA2 DUSP5 TAOK2 USP10 RPS6KB1 FMNL3 RPS6 APOLD1 EIF4E ATF6 FRS2 TPM3 TXN EIF4G2 EIF4G1 RPS6KA5 MAPKAPK2 TKT GLUD1 AMOT DECR1 JUN TMSB10 DNAJB4 KANK1 RAPGEF1 GRB10 GRB2 MAPK14 LIMK1 OCRL |
| 1.14E-05 | 29 | 64 | 2.370901698 | WP4313 Ferroptosis | SLC11A2 SLC1A5 BACH1 STEAP3 HMOX1 CBS CISD1 SAT1 AIFM2 ATG7 TXNRD1 SLC3A2 SLC7A11 FTL PRNP SAT2 FTH1 VDAC2 SLC39A14 TFRC TP53 LPCAT3 HMGCR PCBP2 |

| | | | | | |
|---|---|---|---|---|---|
| | | | | | ACSL4 SLC38A1 COQ2 GCLM POR |
| 3.09E-10 | 63 | 144 | 2.289146467 | WP2380 Brain-derived neurotrophic factor (BDNF) signaling pathway | RPS6KA3 RPS6KB1 RPS6 BCL2L11 CSNK2A1 FOXO3 EIF4EBP1 MEF2C EIF4E SHC2 FRS2 SIRPA PRKAA1 STAT3 YBX1 BMP2 CAMK4 PIK3R1 PIK3R2 RELA RPS6KA5 PDPK1 NTRK3 KIDINS220 DPYSL2 STAT5A MAPK9 MAP2K1 PRKCD CDKL5 CDK5R1 CREB1 FOS SORT1 JAK2 VAV3 PLCG1 EEF2 MAPK1 VAV2 MAP3K2 CHUK APC MTOR AKT1 TSC2 SHC3 JUN NFKB1 NFKBIA GRB2 DOCK3 MAPK14 ELK1 MAPK8 EGR2 RACK1 NCAM1 RAF1 EIF2S1 GSK3B IKBKG BDNF |
| 0.000869305 | 19 | 44 | 2.259417292 | WP4577 Neurodegeneration with brain iron accumulation (NBIA) subtypes pathway | RB1CC1 PRKAA1 STK11 TSC1 SPTLC1 PANK2 DCAF17 C19orf12 MECP2 RHEB ULK1 ATG7 ATG14 FTL WIPI2 AKT1S1 ATG2A MTOR TSC2 |

Table S5. Pathway enrichment. Figure 5E detailed data.

| Enrichment FDR | nGenes | Pathway Genes | Fold Enrichment | Pathway | Genes |
|---|---|---|---|---|---|
| 1.35E-17 | 80 | 156 | 2.683248606 | Path:hsa04218 Cellular senescence | HIPK3 CDK4 CDK6 CDKN1A CDKN2A CDKN2B TRAF3IP2 CHEK1 MAPK14 E2F1 E2F3 E2F5 EIF4EBP1 HIPK1 AKT1 ETS1 RRAS2 MRAS FOXM1 FOXO1 FOXO3 FBXW11 SIRT1 MTOR HIPK2 SLC25A6 IGFBP3 CXCL8 ITPR1 ITPR3 KRAS SMAD2 SMAD3 MDM2 MRE11 MYBL2 MYC ATM NFATC3 NFKB1 NRAS SERPINE1 PIK3CA PIK3CD PIK3R1 PIK3R2 ATR PPP1CA PPP1CC PPP3R1 MAPK1 MAP2K1 MAP2K3 RAF1 RB1 RBL1 RBL2 CCND1 RELA RHEB ZFP36L2 TGFB1 TGFBR1 TGFBR2 TP53 TSC1 TSC2 VDAC1 VDAC2 CALM3 RASSF5 CCNB1 CCND2 BTRC |

| | | | | | |
|---|---|---|---|---|---|
| | | | | | CCND3 CCNE1 MCU CCNE2 MAPKAPK2 CDK1 |
| 2.35E-08 | 36 | 72 | 2.616167391 | Path:hsa04137 Mitophagy-animal | CALCOCO2 GABARAP GABARAPL2 CSNK2A1 E2F1 RRAS2 MRAS FOXO3 GABARAPL1 TBK1 HIF1A RAB7B JUN KRAS NBR1 MITF NRAS RHOT1 AMBRA1 MFN1 MAPK8 MAPK9 RELA RPS27A TBC1D15 SP1 TP53 UBA52 UBC ATG9A TBC1D17 ULK1 TAX1BP1 RHOT2 USP8 MFN2 |
| 6.28E-11 | 56 | 119 | 2.462275191 | Path:hsa04722 Neurotrophin signaling pathway | SH2B3 FRS2 PRDM4 IRAK3 CRK CRKL MAPK14 AKT1 FOXO3 GAB1 SHC2 BEX3 GRB2 RAPGEF1 GSK3B FASLG IRAK1 JUN KRAS RHOA ARHGDIA MAP3K3 MAP3K5 NFKB1 NFKBIA NRAS NTRK3 IRAK4 PDPK1 PIK3CA PIK3CD PIK3R1 PIK3R2 PLCG1 SHC3 PRKCD MAPK1 MAPK8 MAPK9 MAP2K1 KIDINS220 RAF1 BCL2 RELA RPS6KA3 BDNF SORT1 TP53 CALM3 CAMK4 CAMK2B CAMK2D CAMK2G RPS6KA5 MAPKAPK2 MAGED1 |
| 5.94E-12 | 65 | 141 | 2.412069226 | Path:hsa04140 Autophagy-animal | TANK ATG7 CAMKK2 GABARAP GABARAPL2 CTSD DAPK3 EIF2S1 AKT1 ERN1 RRAS2 MRAS ATG14 ATG2A GABARAPL1 MTOR WIPI2 TBK1 HIF1A HMGB1 RAB7B IGF1R ITPR1 KRAS LAMP2 RAB8A NRAS SH3GLB1 PDPK1 PIK3CA PIK3CD PIK3R1 PIK3R2 DDIT4 STX17 ATG2B PRKAA1 AMBRA1 PRKACB PRKCD MAPK1 MAPK8 MAPK9 MAP2K1 RRAGD RAB1A RAF1 BCL2 RHEB RPS6KB1 MTMR14 STK11 MAP3K7 TSC1 TSC2 UVRAG ATG9A VMP1 ULK1 AKT1S1 IRS4 VAMP8 MTMR3 MTMR4 RUBCN |
| 1.72E-08 | 48 | 109 | 2.304147427 | Path:hsa04066 HIF-1 signaling pathway | CDKN1A CDKN1B EGLN2 EGLN3 EGFR EIF4E EIF4EBP1 ENO1 EP300 AKT1 MTOR GAPDH MKNK2 HIF1A HK1 |

| | | | | | |
|---|---|---|---|---|---|
| | | | | | HMOX1 IFNG IGF1R LDHB NFKB1 SERPINE1 PDHB PFKFB3 PFKM PFKP PGK1 PIK3CA PIK3CD PIK3R1 PIK3R2 PLCG1 PRKCA PRKCB MAPK1 MAP2K1 BCL2 RELA RPS6 RPS6KB1 STAT3 ELOC ELOB TFRC VEGFA CAMK2B CAMK2D CAMK2G CUL2 |
| 0.000699268 | 18 | 41 | 2.297122587 | Path:hsa04216 Ferroptosis | ATG7 SAT2 ACSL4 SLC39A14 SLC7A11 FTH1 FTL GCLM HMOX1 SLC11A2 PCBP2 STEAP3 PRNP SAT1 SLC3A2 TFRC TP53 VDAC2 |
| 7.04E-13 | 104 | 266 | 2.045724877 | Path:hsa05012 Parkinson disease | PPIF TRAP1 TUBB3 TUBB4B ADRM1 PARK7 COX4I1 COX6A1 COX6B1 COX7B ADORA2A EIF2S1 ERN1 ATF6 SLC39A14 SLC39A6 TXN2 GNAI1 GNAI2 GNAI3 GNAL GNAS SLC25A6 HSPA5 TUBB2B ITPR1 ITPR3 KIF5A KIF5B MAP3K5 MT-ATP6 MT-CO1 MT-CO2 MT-CO3 MT-CYB MT-ND2 MT-ND4 MT-ND5 NDUFA2 NDUFA4 NDUFA5 NDUFA7 NDUFA9 NDUFB6 NDUFB10 NDUFV3 NFE2L2 SLC11A2 ATP5F1A ATP5F1B ATP5F1C UBE2J1 ATP5MC2 ATP5MC3 ATP5PF PLCG1 CYCS SLC39A9 MFN1 PRKACB MAPK8 MAPK9 PSMA4 PSMA7 PSMB5 PSMB6 PSMC1 PSMC2 PSMC3 PSMD2 PSMD3 PSMD4 PSMD8 PSMD9 PSMD11 PSMD12 SLC39A10 PSMD13 RPS27A SDHA SDHD KLC2 SNCA TP53 TUBB2A TXN UBA52 UBC UBA1 UBE2L3 UCHL1 UQCRC1 UQCRFS1 VDAC1 VDAC2 XBP1 TUBA1A CALM3 CAMK2B CAMK2D CAMK2G TUBA1C MCU COX5A |
| 0.000862493 | 23 | 59 | 2.039723728 | Path:hsa04370 VEGF signaling pathway | MAPK14 AKT1 PLA2G4F SHC2 PLA2G4D KDR KRAS NRAS PIK3CA PIK3CD PIK3R1 PIK3R2 PLCG1 PPP3R1 PRKCA PRKCB MAPK1 MAP2K1 PTGS2 |

| | | | | | |
|---|---|---|---|---|---|
| | | | | | PXN RAF1 VEGFA MAPKAPK2 |
| 2.84E-07 | 53 | 136 | 2.039071643 | Path:hsa04210 Apoptosis | BCL2L11 CHUK CTSD CTSS DAB2IP EIF2S1 AKT1 ERN1 FOS BBC3 XIAP BIRC5 FAS FASLG ITPR1 ITPR3 JUN KRAS MCL1 MAP3K5 ATM NFKB1 NFKBIA NRAS PDPK1 PIK3CA PIK3CD PIK3R1 PIK3R2 PMAIP1 CYCS MAPK1 MAPK8 MAPK9 MAP2K1 DIABLO BAK1 PTPN13 RAF1 BCL2 RELA ACTB BID ACTG1 TP53 TUBA1A CASP2 TUBA1C LMNB2 IKBKG TNFSF10 TNFRSF10B TNFRSF10A |
| 0.001093854 | 23 | 60 | 2.005728333 | Path:hsa04730 Long-term depression | PLA2G4F GNA12 GNAI1 GNAI2 GNAI3 GNAQ GNAS PLA2G4D GUCY1B1 IGF1R ITPR1 ITPR3 KRAS LYN NRAS PPP2R1A PPP2R1B PRKCA PRKCB PRKG1 MAPK1 MAP2K1 RAF1 |

Table S6. Pathway enrichment. Figure 5F detailed data.

| Enrichment FDR | nGenes | Pathway Genes | Fold Enrichment | Pathway | Genes |
|---|---|---|---|---|---|
| 0.003719282 | 5 | 6 | 4.360278985 | Neurons Necrosis Caused by Energy Deficiency | PDPK1 PLCG1 CYCS ATP2B1 SOD2 |
| 0.003719282 | 5 | 6 | 4.360278985 | Brain Cell Necrosis in Vascular Dementia | PDPK1 PLCG1 CYCS ATP2B1 SOD2 |
| 9.20E-06 | 16 | 25 | 3.34869426 | Blood-Brain Barrier Disruption in Epileptiform Disorders | PDPK1 RPS6 TSC2 TSC1 F2 MTOR TGFBR1 TGFBR2 RHEB RPS6KB1 GNAQ EIF4EBP1 ITPR1 GJA1 AKT1 EIF4E |
| 0.002762988 | 8 | 13 | 3.219898327 | Ca2+ Cytosolic Overload | PDPK1 ATP2B1 SLC8A1 SOD2 NFKBIA AKT1 PLCG1 CYCS |
| 0.002762988 | 8 | 13 | 3.219898327 | BDKRB1/2 Vasodilation | PDPK1 GNAI1 GNAI2 GNAI3 GNA12 GNAQ AKT1 GNAS |
| 0.001268909 | 11 | 20 | 2.87778413 | Vascular Smooth Muscle Cell/Pericyte Differentiation and Proliferation | TGFB1 SMAD3 SMAD2 SMAD5 SMAD4 SMAD1 SRF GATA6 TGFBR1 KLF4 TGFBR2 |

| | | | | | |
|---|---|---|---|---|---|
| 8.99E-08 | 30 | 55 | 2.85400079 | Endothelial Cell Dysfunction in Glomerulonephritis | JUN IRAK4 VCAM1 VEGFA MAPK14 NFKBIA IL6ST RAF1 CXCL8 ITPR1 IRAK1 AKT1 MAP3K7 MAP3K5 MAP2K4 MAP2K3 F2R MAP2K1 PDPK1 STAT3 FOS F2 PRKCA GNAQ F2RL3 HIF1A ICAM1 MAPK1 JAK1 JAK2 |
| 0.002577667 | 14 | 31 | 2.362989901 | Vascular Endothelial Cell Activation by NO | HSP90AA1 CAV1 F3 VEGFA NFKBIA GNAQ VDAC1 SERPINE1 SLC7A1 PTGS2 GJA1 AKT1 KDR PLCG1 |
| 9.48E-05 | 25 | 57 | 2.294883676 | Vascular Endothelial Cell Activation by Blood Coagulation Factors | JUN IRAK4 VCAM1 VEGFA NFKBIA RAF1 CXCL8 TFPI ITPR1 IRAK1 AKT1 F2R MAP2K1 PDPK1 STAT3 FOS F2 PRKCA GNAQ F2RL1 F2RL3 YBX1 ICAM1 MAPK1 JAK2 |
| 0.001634858 | 20 | 50 | 2.092933913 | Smooth Muscle Cell Dysfunction in Arterial Hypertension | RHOA JUN CACNA2D1 PIK3CA ITPR1 MYC AKT1 PRKCD MAP2K1 PDPK1 FOS MTOR CACNB2 PIK3R1 GNAQ KRAS GRB2 SRF ELK1 MAPK1 |

Table S7. Pathway enrichment. Figure 6A detailed data.

| Enrichment FDR | nGenes | Pathway Genes | Fold Enrichment | Pathway | Genes |
|---|---|---|---|---|---|
| 0.021441272 | 5 | 9 | 3.92093358 | P00015 Circadian clock system | CLOCK CRY2 PER1 PER2 PER3 |
| 0.001701263 | 13 | 31 | 2.959672444 | P00020 FAS signaling pathway | FAS FASLG CASP3 CASP7 CASP8 CASP9 PARP4 FAF1 JUN LMNB1 MAP2K4 MAP3K5 MAPK9 |
| 0.000466499 | 18 | 46 | 2.761701043 | P00046 Oxidative stress response | DUSP22 DUSP18 DUSP21 DUSP1 DUSP12 DUSP2 DUSP7 ELK1 JUN MAP2K3 MAP2K4 MAPK14 MAPK9 MEF2C MKNK1 MKNK2 MYC BCL2 |
| 2.37E-08 | 42 | 108 | 2.744653506 | P00006 Apoptosis signaling pathway | TMBIM6 TNFRSF10A TNFRSF10B FAS TNFSF10 FASLG |

| | | | | | |
|---|---|---|---|---|---|
| | | | | | TP53 CASP3 CASP7 CASP8 CASP9 CYCS DIABLO CRADD EIF2S1 ENDOG AKT1 BCL2L2 HSPA8 IGF2R XIAP JUN MADD MAP2K3 MAP2K4 MAP3K5 MAP4K3 MAPK1 MAPK9 MCL1 NFKB1 ATF3 AIFM1 PIK3CA PIK3CG PRKCB PRKCD BAG3 BCL2 BCL2L11 RELA RELB |
| 0.049348229 | 7 | 19 | 2.600198058 | P00007 Axon guidance mediated by semaphorins | DPYSL5 DPYSL2 FES FRK NRP1 PAK1 PLXNA1 |
| 3.12E-05 | 29 | 79 | 2.590794087 | P00059 p53 pathway | RRM2 SUMO2 TNFRSF10A TNFRSF10B FAS TP53 CCNB1 CCNE1 CHEK2 CDK2 CDKN1A CDKN2D DDB2 ZMAT3 EP300 AKT1 GADD45A HMGB1 IGFBP3 MDM2 MTA2 KAT2B PDPK1 SERPINB5 PIK3CA PIK3CG PMAIP1 PPM1D PTEN |
| 0.012534681 | 12 | 35 | 2.419776152 | P05911 Angiotensin II-stimulated signaling through G proteins and beta-arrestin | GRK2 GRK3 ELK1 AGT AGTR1 GNB1 GNB3 GNB5 MAPK1 ARRB1 PLCB2 RAF1 |
| 0.003498428 | 16 | 47 | 2.402614619 | P00054 Toll receptor signaling pathway | TBK1 TLR4 TNFAIP3 TLR7 IRAK4 TAB1 ELK1 IRAK1 JUN MAP2K3 MAP3K8 MAPK14 MAPK9 NFKBIE PTGS2 RELA |

| | | | | | |
|---|---|---|---|---|---|
| 0.000133653 | 30 | 91 | 2.326707839 | P00052 TGF-beta signaling pathway | BMP2 BMP8B BMPR1A BMPR2 SKI SKIL TGFBR1 TGFBR2 ACVR1 ACVR1B ACVR2A TAB1 BAMBI EP300 FKBP1B FKBP2 GDF10 GDF11 JUN JUND SMAD1 SMAD3 SMAD4 SMAD5 SMAD6 SMAD7 ZFYVE9 MAPK1 MAPK14 MAPK9 |
| 0.007339669 | 15 | 46 | 2.301417536 | P04398 p53 pathway feedback loops 2 | TP53 CCNE1 CDK2 CDKN1A AKT1 MAPK14 MDM2 MYC PDPK1 PIK3CA PIK3CG PPM1D PTEN RB1 RBL1 |

Table S8. Pathway enrichment. Figure 6B detailed data.

| Enrichment FDR | nGenes | Pathway Genes | Fold Enrichment | Pathway | Genes |
|---|---|---|---|---|---|
| 0.046568152 | 5 | 9 | 3.92 | DOID:0050852 limb ischemia | HGF HMOX1 GRK2 VEGFA HIF1A |
| 0.026479846 | 6 | 11 | 3.85 | DOID:3602 toxic encephalopathy | EPHA4 IGF1R ABCB1 ABCG2 GSTP1 EPHX1 |
| 0.015615717 | 10 | 24 | 2.94 | DOID:10941 intracranial aneurysm | CTSS CST3 MMP2 BCL2 CCL2 TIMP2 TIMP1 PKD2 PKD1 COL1A2 |
| 0.001326919 | 17 | 43 | 2.79 | DOID:3407 carotid artery disease | VCAM1 HSPD1 HGF IGF1 NAMPT PAPPA CTSS MMP2 MMP8 XDH TLR4 GNB3 AGTR1 PPARG SOD2 AGT GSTP1 |
| 0.025426888 | 11 | 30 | 2.59 | DOID:10908 hydrocephalus | ITGB1 TMEM67 ADD1 L1CAM HMGB1 SOX3 HSPD1 NME5 E2F5 |

| | | | | | |
|---|---|---|---|---|---|
| | | | | | CCDC88C MPDZ |
| 0.001967399 | 19 | 53 | 2.53 | DOID:3312 bipolar disorder | NDUFS7 L1CAM GRK3 PTGS2 SRSF3 ETV4 ATP1A3 POLG ETV1 TTK CLOCK CCL2 GSK3B AKT1 SYNE1 GABRA3 BRD1 SLC6A4 GCH1 |
| 1.30E-07 | 60 | 193 | 2.19 | DOID:224 transient cerebral ischemia | NAMPT BCL2L2 BCL2 SLC17A6 RACK1 GHSR MFN2 NFKB1 DUSP1 EED CAV1 JAK1 MMP2 PTPN13 OPTN MAP3K5 BACE1 PTGER4 GSR HTT CASP3 IGF1 HIF1A CBL HSPA8 PPARG SOD2 CCR6 MECP2 CCL2 ADAMTS1 FAS TIA1 PPP1R15A XRCC1 CASP9 ESR2 EP300 MDM2 GLRX STAT3 CREB1 C3 ATP5F1B MAP2K4 SMPD3 WNK1 AGT ADARB1 G6PD ELK1 MADD AIFM1 CASP7 GJA1 PCNA CASP8 EIF2S1 MFF DBN1 |
| 3.93E-05 | 43 | 142 | 2.14 | DOID:2316 brain ischemia | CST3 MDK ANXA1 IGF1 PTGIS NPTXR CREB1 LIF ESR2 LONP1 FASLG C3 PRKCD MMP2 PTGS2 IRAK1 AKT1 HIF1A |

| | | | | | |
|---|---|---|---|---|---|
| | | | | | BTG2 CTSV ABCB1 HMOX1 HSPA8 CCL2 APEX1 AKAP12 PPP1R15A PAK1 ADAMTS1 SLC29A1 PRKACB CLU ENDOG FAS DPYSL2 BCL2 IRAK4 F2R NR3C1 CAV1 GJA1 PCNA TLR4 |
| 0.00012129 | 40 | 134 | 2.11 | DOID:11832 visual epilepsy | APP FAS DRD3 PDE4A KDM3B BRD1 GRM8 VDAC1 PTGS2 SLC38A2 CCNB1 CCND1 MECP2 XRCC1 ANKH MAP1B GJA1 GRIK5 GLUL CIT IFNG BAG3 ABCB1 NR3C1 PRICKLE1 PDGFB APEX1 ADAMTS1 PCSK1 YWHAZ SH3GL2 MT-CYB NFE2L2 VTN DUSP1 MAP3K5 PAFAH1B1 ENDOG L2HGDH ASPM |
| 0.015615717 | 20 | 68 | 2.08 | DOID:2349 arteriosclerosis | TGFBR2 IGFBP3 IGF1 HGF PAPPA APOH CD36 MGP LDLR MMP2 HSPD1 TNFSF10 RELA PPARG TLR4 LCAT PTGS2 ITGA2 ABCG8 PTGER4 |

Table S9. Pathway enrichment. Figure 6C detailed data.

| Enrichment FDR | nGenes | Pathway Genes | Fold Enrichment | Pathway | Genes |
|---|---|---|---|---|---|
| 7.15E-18 | 70 | 161 | 3.068556715 | HALLMARK APOPTOSIS | CASP3 CASP9 CASP7 PMAIP1 CASP8 JUN BCL2L11 MCL1 DIABLO BID CDKN1A GADD45A TNFSF10 SQSTM1 FASLG EGR3 FAS CCND1 F2R CASP4 RHOB CCND2 XIAP TIMP1 ADD1 TIMP2 BTG2 TIMP3 IGF2R CDC25B PPP3R1 HGF CLU ATF3 CDK2 SMAD7 HMOX1 GCH1 MMP2 APP BRCA1 SOD2 EREG KRT18 RELA WEE1 PEA15 CASP2 CTH PLCB2 BMP2 HMGB2 TGFBR3 TXNIP ANKH CYLD GSR PPT1 IFITM3 CAV1 DNM1L ANXA1 TOP2A MADD SAT1 BCL2L2 PAK1 FDXR GPX4 ETF1 |
| 4.59E-06 | 22 | 54 | 2.875351292 | HALLMARK TGF BETA SIGNALING | TGFBR1 SMAD7 BMPR2 SKIL SKI ACVR1 NCOR2 SMAD1 SMAD6 PPP1R15A TGIF1 SMAD3 BMPR1A KLF10 BMP2 APC XIAP CDH1 ID1 CDK9 ARID4B PPP1CA |
| 6.56E-15 | 74 | 200 | 2.611341764 | HALLMARK MTORC1 SIGNALING | DDIT4 PGK1 SLC7A5 ACSL3 SLC1A5 M6PR TMEM97 IFRD1 PLOD2 PSAT1 MTHFD2 FADS2 VLDLR WARS1 SCD ACTR2 SERPINH1 NAMPT CDKN1A EGLN3 LGMN PNP SLC7A11 SSR1 SQSTM1 PDK1 SERP1 TRIB3 HMGCS1 ASNS GAPDH IMMT INSIG1 IGFBP5 FGL2 IDI1 SHMT2 TXNRD1 G6PD LDLR TES ITGB2 AK4 SLC6A6 BTG2 NMT1 PSMC6 FDXR RRM2 CTH POLR3G QDPR RPN1 HSPA4 NIBAN1 TBK1 PSMD13 GLRX ETF1 GSK3B PHGDH LTA4H FKBP2 CXCR4 TCEA1 MAP2K3 HSPD1 PPP1R15A GSR |

| | | | | | |
|---|---|---|---|---|---|
| | | | | | PDAP1 BUB1 ATP2A2 ABCF2 CCT6A |
| 6.56E-15 | 74 | 200 | 2.611341764 | HALLMARK TNFA SIGNALING VIA NFKB | ATF3 TNFAIP3 PTGS2 KLF6 JUN EGR3 PPP1R15A SOD2 RELB TRAF1 BTG2 DUSP1 MAP3K8 F3 KDM6B NFKB1 LIF JAG1 GCH1 CCL2 DUSP2 EHD1 NFKBIE NR4A3 PHLDA1 IER5 TNFSF9 GEM GADD45A EGR2 PTGER4 SERPINB2 TRIB1 TIPARP RELA CXCL6 MYC NAMPT PNRC1 IL7R PFKFB3 YRDC SQSTM1 BTG1 MCL1 VEGFA MAP2K3 CDKN1A CCN1 RHOB SAT1 GPR183 KLF10 TRIP10 F2RL1 LDLR TGIF1 RNF19B DNAJB4 PDE4B CCND1 SIK1 NFE2L2 PER1 NFAT5 ATP2B1 ABCA1 SMAD3 IFIH1 BCL3 FJX1 EIF1 BMP2 PDLIM5 |
| 1.81E-08 | 41 | 113 | 2.560751312 | HALLMARK UNFOLDED PROTEIN RESPONSE | HERPUD1 ATF3 ASNS DNAJB9 KHSRP CNOT4 ATP6V0D1 SERP1 SLC7A5 SSR1 EIF2AK3 MTHFD2 SRPRA ERN1 FKBP14 YIF1A CCL2 EIF4A1 EIF2S1 CHAC1 PDIA5 NABP1 LSM1 EIF4A2 ALDH18A1 DDIT4 EIF4A3 POP4 CEBPG BAG3 CNOT6 PSAT1 YWHAZ MTREX KIF5B DCP1A IMP3 SPCS1 VEGFA EEF2 PAIP1 |
| 0.00147491 | 13 | 36 | 2.548606827 | HALLMARK ANGIOGENESIS | FSTL1 LRPAP1 VEGFA TNFRSF21 CCND2 KCNJ8 APP JAG1 NRP1 VTN TIMP1 APOH CXCL6 |
| 0.001455244 | 16 | 49 | 2.304548716 | HALLMARK REACTIVE OXYGEN SPECIES PATHWAY | GSR TXNRD1 GPX4 SOD2 PDLIM1 G6PD OXSR1 GLRX2 FES PFKP NDUFB4 CDKN2D STK25 ERCC2 GLRX PRNP |
| 1.30E-05 | 31 | 96 | 2.279042643 | HALLMARK PROTEIN SECRETION | ARCN1 TMED10 IGF2R PPT1 KIF1B ARF1 OCRL ARFGEF2 TMED2 SH3GL2 RAB2A COG2 GNAS VPS45 SNX2 CLTA M6PR SGMS1 TSG101 DNM1L VAMP7 RAB22A GBF1 KRT18 CAV2 ATP1A1 |

| | | | | | |
|---|---|---|---|---|---|
| | | | | | GOLGA4 AP1G1 MAPK1 ABCA1 STX12 |
| 6.72E-10 | 64 | 200 | 2.258457742 | HALLMARK P53 PATHWAY | CDKN1A BTG2 MDM2 FAS TOB1 GADD45A ZMAT3 DDB2 EI24 DDIT4 ATF3 SESN1 FDXR PIDD1 SAT1 AEN PPM1D BTG1 TXNIP SLC19A2 TP53 AK1 CCND2 RALGDS SERPINB5 TNFSF9 PCNA RB1 PPP1R15A HSPA4L EPHX1 IER5 TAX1BP3 WRAP73 PLXNB2 HEXIM1 NOL8 SP1 CCP110 TRAFD1 RAD51C TRIB3 CTSD ELP1 EPHA2 JUN SLC7A11 IRAK1 LIF RPL36 RNF19B CDK5R1 PITPNC1 TPRKB MKNK2 RACK1 LDHB APP F2R ACVR1B BMP2 SLC3A2 DNTTIP2 HMOX1 |
| 2.63E-08 | 60 | 200 | 2.117304133 | HALLMARK HYPOXIA | PGK1 PDK1 MXI1 ADM PFKP AK4 PFKFB3 VEGFA GYS1 GAPDH JUN DDIT4 IGFBP3 RBPJ GALK1 WSB1 BTG1 VLDLR EXT1 F3 ANKZF1 PNRC1 HMOX1 ATF3 CAV1 RRAGD EDN2 CDKN1A RORA DUSP1 HS3ST1 CAVIN1 NR3C1 KLF6 GPC4 CCN1 TNFAIP3 HEXA PPP1R15A PIM1 NAGK SDC3 TIPARP CXCR4 CCN2 BCL2 KLHL24 PDGFB SRPX GLRX TGFBI PLAC8 MYH9 PCK1 INHA SLC6A6 AKAP12 TES COL5A1 SELENBP1 |

Table S10. Pathway enrichment. Figure 6D detailed data.

| Enrichment FDR | nGenes | Pathway Genes | Fold Enrichment | Pathway | Genes |
|---|---|---|---|---|---|
| 1.24E-05 | 14 | 24 | 4.116980259 | WP4925 Unfolded protein response | ERN1 TNFRSF10B NFE2L2 TP53 PMAIP1 EIF2AK3 PPP1R15A EIF2S1 BCL2L11 BCL2 RTCB TXNIP CASP2 BID |
| 0.000133735 | 11 | 19 | 4.08602552 | WP384 Apoptosis | NFKB1 AIFM1 FAS FASLG CASP7 CASP8 CASP9 CASP2 BID CASP3 CYCS |

| | | | | | |
|---|---|---|---|---|---|
| | | | | modulation by HSP70 | |
| 6.23E-05 | 13 | 24 | 3.822910241 | WP1539 Angiogenesis | TIMP2 SMAD1 HIF1A MAPK1 TIMP3 PDGFB VEGFA PDGFRA FGFR2 PIK3CA FGF2 MAPK14 AKT1 |
| 1.90E-05 | 15 | 28 | 3.780900238 | WP5036 Angiotensin II receptor type 1 pathway | TGFBR1 TGFBR2 COL1A2 SP1 RACK1 HIF1A MAPK1 SMAD3 SMAD4 RAF1 ACE2 AGT CCN2 AGTR1 JUND |
| 0.001295871 | 9 | 17 | 3.736419059 | WP368 Mitochondrial long chain fatty acid beta-oxidation | ACADM SCP2 ACADS CPT1A HADH ACSL4 ACSL3 ACSF2 ECI1 |
| 0.007237366 | 7 | 14 | 3.528840222 | WP4724 Omega-9 fatty acid synthesis | FADS2 FASN ACSL4 ACSL3 ACOT2 ELOVL2 SCD |
| 0.046115847 | 4 | 8 | 3.528840222 | WP1995 Effects of nitric oxide | MB XDH AOX1 MT-CO1 |
| 0.019231688 | 6 | 13 | 3.257390974 | WP4919 Neuroinflammation | TLR4 RELA MAPK14 MT-CO1 MT-CO2 JUN |
| 0.048515214 | 5 | 12 | 2.940700185 | WP4495 IL-10 anti-inflammatory signaling pathway | JAK1 BLVRA BLVRB STAT3 HMOX1 |
| 0.008604127 | 9 | 22 | 2.887232909 | WP4482 Vitamin D in inflammatory diseases | NFKB1 SMAD3 RELA SMAD4 DUSP1 MAPK14 NR3C1 PPP3R1 MAP2K3 |

Table S11. Pathway enrichment. Figure 6E detailed data.

| Enrichment FDR | nGenes | Pathway Genes | Fold Enrichment | Pathway | Genes |
|---|---|---|---|---|---|
| 6.73E-09 | 33 | 73 | 3.190458283 | Path:hsa04115 p53 signaling pathway | CDK6 CDKN1A CHEK2 SESN3 DDB2 GADD45A SESN1 IGF1 IGFBP3 FAS MDM2 SERPINB5 PMAIP1 CYCS PIDD1 CCND1 BCL2 RRM2 BID ZMAT3 TP53 TSC2 CASP3 CASP8 CASP9 PPM1D TNFRSF10B TNFRSF10A CCNB1 CCND2 CCNE1 EI24 TP53I3 |
| 0.000573138 | 14 | 34 | 2.906103712 | Path:hsa04710 Circadian rhythm | CREB1 CRY2 FBXL3 NPAS2 PER1 PRKAA1 PRKAB2 RORA RORB RORC BHLHE41 PER3 PER2 CLOCK |

| | | | | | |
|---|---|---|---|---|---|
| 0.027933172 | 8 | 23 | 2.454845372 | Path:hsa03060 Protein export | SEC61G SPCS1 OXA1L SEC61A2 SRP9 SRP14 SRP19 SRPRA |
| 0.010229073 | 11 | 32 | 2.426077653 | Path:hsa04215 Apoptosis-multiple species | XIAP PMAIP1 CYCS MAPK9 DIABLO BCL2 BID CASP3 CASP7 CASP8 CASP9 |
| 2.18E-05 | 31 | 93 | 2.352560148 | Path:hsa04350 TGF-beta signaling pathway | E2F5 EP300 BAMBI RGMB ID1 ID4 IFNG SMAD1 SMAD3 SMAD4 SMAD5 SMAD6 SMAD7 MYC PPP2R1A MAPK1 RBL1 RPS6KB2 GREM2 BMP2 BMP8B BMPR1A BMPR2 SP1 TGFBR1 TGFBR2 TGIF1 ACVR1 ACVR1B ACVR2A ZFYVE9 |
| 0.010229073 | 13 | 41 | 2.237801116 | Path:hsa04216 Ferroptosis | SAT2 ACSL3 ACSL4 SLC7A11 FTH1 GPX4 HMOX1 MAP1LC3C PCBP2 PRNP SAT1 SLC3A2 TP53 |
| 9.60E-05 | 33 | 109 | 2.136728942 | Path:hsa04066 HIF-1 signaling pathway | CDKN1A EGLN3 EP300 AKT1 GAPDH MKNK2 HIF1A HMOX1 IFNG IGF1 IGF1R INSR LDHB NFKB1 PDHA2 PDHB PDK1 PFKFB3 PFKP PGK1 PIK3CA PRKCB MAPK1 BCL2 RELA RPS6KB2 STAT3 ELOC TIMP1 TLR4 VEGFA CAMK2B MKNK1 |
| 0.000349919 | 28 | 93 | 2.124893037 | Path:hsa04657 IL-17 signaling pathway | TRAF3IP2 MAPK14 TBK1 GSK3B HSP90AA1 HSP90AB1 S100A7A IFNG JUN JUND NFKB1 IL17RB MAPK1 MAPK6 MAPK9 PTGS2 RELA S100A7 CCL2 CCL7 CXCL6 CXCL5 TNFAIP3 TRAF3 TRAF5 CASP3 CASP8 IL17RC |
| 2.08E-05 | 40 | 134 | 2.106770282 | Path:hsa00190 Oxidative phosphorylation | COX17 UQCR11 COX6A1 COX6B1 COX7B COX8A NDUFS7 MT-ATP6 MT-CO1 MT-CO2 MT-CO3 MT-CYB MT-ND2 MT-ND4 MT-ND5 NDUFA1 NDUFA2 NDUFA4 NDUFA5 NDUFA7 NDUFB1 NDUFB4 NDUFB5 NDUFB6 NDUFS8 ATP5F1B ATP6V1A ATP6V1B2 ATP6AP1 CYCS NDUFB11 SDHA SDHB UQCRC1 UQCRC2 UQCRFS1 |

| | | | | | |
|---|---|---|---|---|---|
| | | | | | ATP6V0E1 ATP6V0D1 COX7A2L COX5A |
| 0.000170873 | 33 | 112 | 2.079495131 | Path:hsa04668 TNF signaling pathway | DNM1L TAB1 MAP3K8 CREB1 MAPK14 JAG1 AKT1 VEGFD FAS JUN LIF MAP3K5 NFKB1 PIK3CA MAPK1 MAPK9 MAP2K3 PTGS2 RELA BCL3 CCL2 CXCL6 CXCL5 MAP2K4 TNFAIP3 TRAF1 TRAF3 TRAF5 VCAM1 CASP3 CASP7 CASP8 RPS6KA5 |

Table S12. Pathway enrichment. Figure 6F detailed data.

| Enrichment FDR | nGenes | Pathway Genes | Fold Enrichment | Pathway | Genes |
|---|---|---|---|---|---|
| 0.016245364 | 4 | 6 | 4.705120296 | Brain Cell Necrosis in Vascular Dementia | PDPK1 CYCS ATP2B1 SOD2 |
| 0.000260505 | 11 | 20 | 3.881724244 | Vascular Smooth Muscle Cell/Pericyte Differentiation and Proliferation | SMAD3 SMAD5 SMAD4 TAGLN SMAD1 SRF FN1 GATA6 TGFBR1 TGFBR2 ID1 |
| 0.001362508 | 11 | 24 | 3.234770204 | HIF1 Signaling | PFKFB3 EGLN3 TXNRD1 HIF1A SAT2 VEGFA EP300 APEX1 HIF1AN USP20 GAPDH |
| 0.007737927 | 8 | 18 | 3.136746864 | Exocytosis Vesicle Trafficking | RAB3A RAB3IP RAB27A RAB11A RAB10 KIF3A KIF5B RAB8A |
| 0.003441978 | 10 | 23 | 3.068556715 | Macroautophagy Decline | SH3GLB1 RB1CC1 PIK3R4 HMGB1 AMBRA1 ATG14 HMOX1 BCL2 ULK2 ULK1 |
| 9.56E-08 | 33 | 77 | 3.02472019 | Apoptosis | ENDOG CRADD HUWE1 FAS BCL2 GAS2 CYCS TP53 APP DIABLO CASP8 BCL2L11 CASP9 CASP7 CASP4 CHEK2 AKT1 CASP2 CASP3 MAP3K5 MCL1 TNFSF15 TNFRSF10B TNFRSF10A XIAP MDM2 BCL2L2 FAF1 FASLG AIFM1 TNFSF12 TNFSF10 BID |
| 0.00461199 | 10 | 24 | 2.940700185 | Antiphospholipid Antibodies in Endothelial Cell | MAP2K4 JUN IRAK4 VCAM1 F3 MAPK14 IRAK1 APOH MAP3K5 TLR4 |
| 0.004558904 | 12 | 32 | 2.646630167 | ROS in Triggering Vascular Inflammation | VCAM1 F3 AGT VAV1 VEGFA FAS IFNG AGTR1 |

| | | | | | |
|---|---|---|---|---|---|
| | | | | | RASGRF1 FASLG PTGS2 CCL2 |
| 0.009682924 | 11 | 31 | 2.504338222 | Vascular Endothelial Cell Activation by NO | HSP90AA1 CAV1 F3 AGT VEGFA AGTR1 VDAC1 SLC7A1 PTGS2 GJA1 AKT1 |
| 0.000576639 | 20 | 57 | 2.476379103 | Vascular Endothelial Cell Activation by Blood Coagulation Factors | JUN IRAK4 VCAM1 VEGFA TLR4 RAF1 RASGRF1 CDH5 IRAK1 AKT1 CCN2 CCN1 F2R PDPK1 STAT3 F2RL1 F2RL3 YBX1 CCL2 MAPK1 |

Table S13. Pathway enrichment. Figure 7A detailed data.

| Enrichment FDR | nGenes | Pathway Genes | Fold Enrichment | Pathway | Genes |
|---|---|---|---|---|---|
| 0.000119335 | 14 | 35 | 3.8135 | P00033 Insulin/IGF pathway-protein kinase B signaling cascade | TSC1 FOXO1 FOXO3 AKT1 GSK3B IGF1 IGF1R IGF2R INSR IRS4 MDM2 PIK3CG PIK3R1 PTEN |
| 0.000522211 | 12 | 31 | 3.690483871 | P00020 FAS signaling pathway | FAS FASLG CASP3 CASP7 CASP8 CASP9 PARP4 FAF1 APAF1 JUN LMNB1 MAP2K4 |
| 2.94E-05 | 18 | 47 | 3.651223404 | P00048 PI3 kinase pathway | RPS6KB2 CASP9 CCND1 CCND2 FOXO1 FOXO3 AKT1 GADD45A GNAI2 GNAI3 GNB2 GNB3 GNB5 GSK3B INPP5A INSR PIK3R1 PTEN |
| 1.02E-06 | 33 | 108 | 2.913090278 | P00006 Apoptosis signaling pathway | FAS TNFSF10 FASLG TP53 CASP3 CASP7 CASP8 CASP9 CHUK DIABLO CRADD EIF2S1 ENDOG FOS AKT1 HSPA8 IGF2R APAF1 XIAP JUN MADD MAP2K4 MAP4K3 MCL1 NFKB1 ATF3 AIFM1 PIK3CG PRKCD BAG3 BCL2 BCL2L11 RELB |
| 3.94E-05 | 24 | 79 | 2.896329114 | P00059 p53 pathway | FAS TP53 SIRT1 CCNE1 CCNG1 CHEK2 CDK2 CDKN2D DDB2 EP300 AKT1 GADD45A HMGB1 APAF1 MDM2 MTA2 ATM KAT2B SERPINB5 PIK3CG PIK3R1 PMAIP1 PPM1D PTEN |
| 0.005867034 | 13 | 46 | 2.694320652 | P00046 Oxidative stress response | TXN DUSP22 DUSP21 DUSP1 DUSP12 DUSP5 DUSP7 JUN MAP2K4 MEF2C MKNK1 MYC BCL2 |

| | | | | | |
|---|---|---|---|---|---|
| 0.000326662 | 22 | 80 | 2.62178125 | P00036 Interleukin signaling pathway | RPS6KA3 RPS6KA6 SRF STAT3 STAT5A STAT6 CDKN1B CHUK ELK4 FOS FOXO3 AKT1 GSK3B IL13RA2 IL1A IL20RA IL6R CXCL8 CXCR1 MAPK6 MKNK1 MYC |
| 0.000618025 | 23 | 91 | 2.409629121 | P00052 TGF-beta signaling pathway | BMP2 BMP8B BMPR1A BMPR2 SKIL TGFBR1 TGFBR2 ACVR1B TAB1 ZFYVE16 EP300 FKBP1A FKBP1B FKBP2 GDF10 GDF11 JUN SMAD1 SMAD3 SMAD5 SMAD6 SMAD7 ZFYVE9 |
| 0.000936768 | 28 | 125 | 2.13556 | P00047 PDGF signaling pathway | RPS6KA3 RPS6KA6 RPS6KB2 RPS6KC1 SRF STAT3 STAT5A STAT6 VAV1 VAV3 NIN CHUK ARHGAP42 ELF4 ELK4 ETS1 FOS GSK3B ELP1 JUN MAPK6 MKNK1 MYC PDGFB PDGFRA PDGFRL PIK3CG PIK3R1 |
| 8.06E-05 | 43 | 198 | 2.070460859 | P00031 Inflammation mediated by chemokine and cytokine signaling pathway | CCL2 CCL22 CCL27 CCL7 CCL8 STAT3 VAV1 ACTB C5AR1 ACTG1 PAK6 CCR6 CXCR6 SOCS6 CHUK COL12A1 CXCR4 FPR1 AKT1 GNAI2 GNAI3 GNB3 IFNG CXCL8 CXCR1 JUN KRAS RHOG ARPC5 ARRB1 ARRB2 MYH1 MYH10 MYH2 MYH4 PAK1 PIK3CG PLCB2 PLCB4 PRKX PTEN PTGS2 RELB |

Table S14. Pathway enrichment. Figure 7B detailed data.

| Enrichment FDR | nGenes | Pathway Genes | Fold Enrichment | Pathway | Genes |
|---|---|---|---|---|---|
| 0.000698292 | 15 | 43 | 3.325726744 | DOID:3407 carotid artery disease | VCAM1 HSPD1 HGF IGF1 NAMPT PAPPA MMP1 MMP8 XDH TLR4 GNB3 AGTR1 SOD2 AGT GSTP1 |
| 0.012043446 | 11 | 36 | 2.913090278 | DOID:767 muscular atrophy | CCNG1 RELB COL1A2 DAG1 TFRC TRIM63 ENDOG CHUK KRAS CTSV APAF1 |
| 0.028666128 | 10 | 35 | 2.723928571 | DOID:7998 hyperthyroidism | HMOX1 CTSV PPARGC1A VIM IGF1R ANXA5 GSR MCM7 ANXA1 PCSK1 |

| | | | | | |
|---|---|---|---|---|---|
| 0.01253446 | 14 | 53 | 2.518349057 | DOID:3312 bipolar disorder | NDUFS7 L1CAM PTGS2 ETV4 ATP1A3 POLG TTK CLOCK CCL2 GSK3B AKT1 SYNE1 GABRA3 GCH1 |
| 1.34E-07 | 50 | 193 | 2.46988342 | DOID:224 transient cerebral ischemia | NAMPT GOT1 BCL2 SLC17A6 GHSR NFKB1 DUSP1 EED CAV1 MCFD2 BACE1 GSR HTT CASP3 IGF1 HSPA8 SOD2 CCR6 LPAR1 MECP2 CXCL12 CCL2 ADAMTS1 FAS TIA1 CASP9 BBC3 EP300 MDM2 GLRX STAT3 TIAL1 C3 MAP2K4 SMPD3 WNK1 AGT ADARB1 ICAM1 MT2A G6PD MADD AIFM1 ESR1 CASP7 GJA1 PCNA CASP8 EIF2S1 DBN1 |
| 6.02E-10 | 87 | 379 | 2.188486148 | DOID:10763 hypertension | TIMP1 VCAM1 IL6R HSPA8 BCHE PAPPA ADM IGF1 ICAM1 PRKCD EDN2 MMP1 GSR SOD2 XDH BRCA1 HMOX1 CASP3 CASP8 PIK3R1 NFE2L2 CCL2 GJA5 PDGFRA ATP6AP2 ENO1 CYP4F11 BCL2 CYP4F8 CYP4A22 ADRA2A PTGS2 AGTR1 ACE2 GNAI3 HSPD1 ATP5F1A HSD3B1 MME ADD3 AGT TAC3 CASP9 JUN ATP2B1 CAV1 PNMT CHST12 CYP4A11 DUSP1 GSK3B CD36 STS IGF1R EP300 SH2B3 EIF4G2 CYP11B2 GNAI2 CCNE1 ANXA1 PTPRJ GRK2 ATP1A1 QDPR APP KCNA5 F3 FN1 PDGFB GJA1 CLOCK ARRB2 SRF DRD3 AKT1 FOS RGS5 C3 WNK1 HGF RASGRP3 GNB3 FMO3 ARNTL2 CACNB2 HTRA1 |
| 0.049783516 | 13 | 57 | 2.174364035 | DOID:0050589 inflammatory bowel disease | TPMT PDGFB FGF23 STAT3 SLC6A14 MST1 EPB41L2 IL2 EPB41L3 ALPI ICAM1 AGT ADAM17 |
| 0.041574226 | 15 | 68 | 2.103033088 | DOID:2349 arteriosclerosis | TGFBR2 IGF1 HGF PAPPA CD36 MGP LDLR HSPD1 MMP1 TNFSF10 TLR4 LCAT PTGS2 ITGA2 ESR1 |
| 0.002748818 | 28 | 127 | 2.101929134 | DOID:3525 middle cerebral artery infarction | PARK7 HMOX1 HDAC5 GHSR GSK3B HSPA4 IL7R APAF1 APP EIF2S1 IRF4 CASP3 SNAP91 CASP9 PDGFRA SIRT1 AGT AIFM1 |

| | | | | | |
|---|---|---|---|---|---|
| | | | | | HAS2 C3 DICER1 HMGB1 ACE2 NFE2L2 RPL28 NAE1 BCL2 NAMPT |
| 3.80E-09 | 86 | 393 | 2.086265903 | DOID:10652 Alzheimer's disease | VIM SNRNP70 EP300 DBN1 PAK1 C1QB IL7 AKAP5 EPHX1 PEBP1 NAE1 CASP3 ANXA5 CAST IL1A EEF2 IL3 HMOX1 CASP9 HSPD1 GSR DRD3 IL6R CST3 FOXO3 APP AGTR1 BCL2 BPTF IGF1R AIFM1 BCHE FAS EIF2S1 CDK5R1 CASP8 NDUFA5 CASP7 CTSD SNAP91 PPARGC1A ATM C5AR1 IGF1 CSNK1A1 PRKN L1CAM PDGFB HSP90AA1 PTGS2 AGT CCNG1 CD36 IL2 INSR MME ENO1 STAT3 SOD2 NFE2L2 MT2A RCOR1 HIVEP3 SOX5 BACE1 GSK3B CIB1 HIVEP1 CAV1 ATP5F1A SIRT1 ADAM17 TLR4 ABCA1 PIK3R1 LDLR C3 PCK1 GSTP1 LRPAP1 CLOCK ICAM1 ESR1 PPP3R1 APBB2 PNMT |

Table S15. Pathway enrichment. Figure 7C detailed data.

| Enrichment FDR | nGenes | Pathway Genes | Fold Enrichment | Pathway | Genes |
|---|---|---|---|---|---|
| 5.66E-15 | 56 | 161 | 3.316086957 | HALLMARK APOPTOSIS | CASP3 CASP9 CASP7 PMAIP1 CASP8 JUN BCL2L11 MCL1 DIABLO IL1A BID GADD45A CDKN1B TNFSF10 FASLG EGR3 FAS CCND1 CASP4 CCND2 XIAP TIMP1 IRF1 BTG2 TIMP3 IGF2R CDC25B PPP3R1 HGF ATF3 CDK2 SMAD7 HMOX1 GCH1 APP BRCA1 SOD2 BMF KRT18 WEE1 CTH PLCB2 BMP2 HMGB2 TXNIP GSR MGMT PPT1 IFITM3 CAV1 ANXA1 MADD PAK1 FDXR GPX4 ETF1 |
| 3.74E-09 | 36 | 113 | 3.037300885 | HALLMARK UNFOLDED PROTEIN RESPONSE | HERPUD1 HSP90B1 ATF3 EXOSC1 ASNS CNOT4 DCP2 PDIA6 ATP6V0D1 SERP1 SSR1 EIF2AK3 SRPRA NOLC1 FKBP14 YIF1A CCL2 EIF4A1 EIF2S1 |

| | | | | | |
|---|---|---|---|---|---|
| | | | | | TATDN2 CHAC1 PDIA5 NABP1 LSM1 DDIT4 EIF4A3 POP4 BAG3 CNOT6 PSAT1 MTREX DCP1A IMP3 TUBB2A EXOSC10 EEF2 |
| 9.05E-15 | 63 | 200 | 3.00313125 | HALLMARK TNFA SIGNALING VIA NFKB | ATF3 TNFAIP3 PTGS2 KLF6 ICAM1 JUN EGR3 SOD2 IL1A RELB TRAF1 BTG2 DUSP1 F3 NFKB1 LIF JAG1 GCH1 CCL2 EHD1 NFKBIE NR4A3 PHLDA1 IER5 TNFSF9 GEM GADD45A DUSP5 SERPINB2 TRIB1 TIPARP CXCL6 MYC NAMPT PNRC1 IL7R PFKFB3 BTG1 MCL1 CCN1 TUBB2A IRF1 FOS ZBTB10 PLPP3 GPR183 KLF10 LDLR DRAM1 DNAJB4 PDE4B STAT5A CCND1 TNFAIP8 FUT4 NFE2L2 ATP2B1 ABCA1 HES1 SMAD3 IFIH1 BCL3 BMP2 |
| 0.000192616 | 15 | 49 | 2.918494898 | HALLMARK REACTIVE OXYGEN SPECIES PATHWAY | GSR TXNRD1 GPX4 TXN SOD2 PRDX4 PDLIM1 G6PD OXSR1 GLRX2 FES PFKP NDUFB4 CDKN2D GLRX |
| 0.000183541 | 16 | 54 | 2.824814815 | HALLMARK TGF BETA SIGNALING | TGFBR1 SMAD7 BMPR2 SKIL SMAD1 SMAD6 SMAD3 FKBP1A BMPR1A HIPK2 KLF10 BMP2 XIAP CDH1 ID1 CDK9 |
| 1.01E-12 | 59 | 200 | 2.81245625 | HALLMARK P53 PATHWAY | BTG2 MDM2 CCNG1 FAS TOB1 GADD45A DDB2 EI24 DDIT4 ATF3 SESN1 FDXR PIDD1 AEN PPM1D FOXO3 BTG1 TXNIP SLC19A2 TP53 FOS CCND2 APAF1 SERPINB5 TNFSF9 PCNA RB1 EPHX1 IER5 TAX1BP3 RGS16 ZFP36L1 WRAP73 PLXNB2 HEXIM1 DRAM1 TRAFD1 RAD51C TRIB3 CTSD ELP1 POM121 EPHA2 JUN SLC7A11 IRAK1 LIF CDK5R1 PITPNC1 TPRKB IL1A CCNK LDHB APP ACVR1B BMP2 PTPN14 SLC3A2 HMOX1 |
| 1.08E-09 | 53 | 200 | 2.52644375 | HALLMARK MTORC1 SIGNALING | DDIT4 ACSL3 TFRC IFRD1 PLOD2 PSAT1 CORO1A FADS2 WARS1 SCD SERPINH1 NAMPT EGLN3 |

| | | | | | |
|---|---|---|---|---|---|
| | | | | | LGMN SLC7A11 SSR1 PDK1 SERP1 TRIB3 HMGCS1 GOT1 ASNS FGL2 ENO1 IDI1 TXNRD1 G6PD LDLR ITGB2 AK4 SLC6A6 ADD3 BTG2 CCNG1 PSMC6 FDXR CTH POLR3G QDPR RPN1 HSPA4 GLRX ETF1 GSK3B PHGDH GMPS LTA4H FKBP2 CXCR4 HSPD1 HSP90B1 GSR ATP2A2 |
| 7.81E-09 | 51 | 200 | 2.43110625 | HALLMARK INFLAMMATORY RESPONSE | CCL2 FPR1 IL1A CXCL8 CCL7 CCL22 CXCL9 ICAM1 LIF NAMPT ADM NFKB1 TNFSF10 TNFSF15 CXCL6 LYN LDLR F3 CD55 GCH1 MYC IL7R TNFSF9 TLR1 LPAR1 ABCA1 GNAI3 SLC7A1 TIMP1 GPR183 ATP2B1 ACVR1B KLF6 CXCR6 RGS16 SLAMF1 RHOG HAS2 DCBLD2 LAMP3 SLC31A1 PDE4B ATP2A2 FZD5 ROS1 BTG2 KIF1B C5AR1 EMP3 MEP1A IRF1 |
| 1.86E-08 | 50 | 200 | 2.3834375 | HALLMARK HYPOXIA | PDK1 MXI1 ADM ENO1 PFKP AK4 PFKFB3 GYS1 JUN DDIT4 FOS GALK1 BTG1 F3 PDK3 PNRC1 HMOX1 ATF3 CAV1 EDN2 DUSP1 KLF6 GPC4 CCN1 TNFAIP3 HEXA PIM1 NAGK CDKN1B MT2A SDC3 TIPARP CXCR4 CCN2 PPARGC1A BCL2 KLHL24 FOXO3 PDGFB SRPX GLRX TGFBI PLAC8 PCK1 INHA SLC6A6 COL5A1 HDLBP ETS1 SELENBP1 |
| 0.014413919 | 9 | 36 | 2.3834375 | HALLMARK ANGIOGENESIS | FSTL1 LRPAP1 CCND2 COL5A2 APP JAG1 VTN TIMP1 CXCL6 |

Table S16. Pathway enrichment. Figure 7D detailed data.

| Enrichment FDR | nGenes | Pathway Genes | Fold Enrichment | Pathway | Genes |
|---|---|---|---|---|---|
| 0.021540861 | 4 | 8 | 4.766875 | WP5133 Non-classical role of vitamin D | CYP27B1 AGT ACE2 AGTR1 |

| | | | | | |
|---|---|---|---|---|---|
| 0.000157379 | 11 | 24 | 4.369635417 | WP4925 Unfolded protein response | NFE2L2 TP53 PMAIP1 EIF2AK3 EIF2S1 BCL2L11 BCL2 RTCB BBC3 TXNIP BID |
| 2.39E-05 | 16 | 40 | 3.8135 | WP127 IL-5 signaling pathway | RPS6KB2 PTPN11 STAT3 FOS IL2 BCL2 MYC FOXO3 PIK3CG PIK3R1 LYN GSK3B STAT5A AKT1 KRAS JUN |
| 0.000987357 | 10 | 25 | 3.8135 | WP205 IL-7 signaling pathway | STAT3 CCND1 MYC IL7R PIK3R1 IL7 GSK3B CDKN1B STAT5A AKT1 |
| 0.04362728 | 4 | 10 | 3.8135 | WP3656 Interleukin-1 induced activation of NF-kB | NFKB1 UBE2N IL1A IRAK1 |
| 8.79E-10 | 34 | 87 | 3.725833333 | WP254 Apoptosis | IGF1R MCL1 BCL2L11 BCL2 FAS MYC IGF1 FASLG APAF1 BID CASP3 CASP4 DIABLO TNFSF10 CHUK CASP7 CASP8 CASP9 AKT1 CRADD JUN TRAF3 NFKB1 PMAIP1 NFKBIE IRF4 TRAF1 MDM2 XIAP IRF1 PIK3R1 TP53 BBC3 MAP2K4 |
| 0.026850452 | 5 | 13 | 3.666826923 | WP4919 Neuroinflammation | TLR4 FOS CHUK MT-CO2 JUN |
| 8.33E-06 | 20 | 55 | 3.466818182 | WP560 TGF-beta receptor signaling | TGFBR1 TGFBR2 FOS EP300 SMAD5 SMAD6 SMAD7 FKBP1A ZNF423 JUN NFKB1 SKIL STAT3 IFNG LIF ZEB2 SMAD1 SMAD3 ZFYVE9 RUNX2 |
| 0.035294613 | 5 | 14 | 3.404910714 | WP4724 Omega-9 fatty acid synthesis | FADS2 FASN ACSL3 ELOVL2 SCD |
| 0.021540861 | 6 | 17 | 3.364852941 | WP4493 Cells and molecules involved in local acute inflammatory response | VCAM1 IL1A ICAM1 CXCL8 ITGB2 C3 |

Table S17. Pathway enrichment. Figure 7E detailed data.

| Enrichment FDR | nGenes | Pathway Genes | Fold Enrichment | Pathway | Genes |
|---|---|---|---|---|---|
| 1.05E-07 | 27 | 73 | 3.526181507 | Path:hsa04115 p53 signaling pathway | CDK6 CHEK2 DDB2 GADD45A BBC3 SESN1 APAF1 IGF1 FAS MDM2 ATM SERPINB5 PMAIP1 PIDD1 CCND1 BCL2 BID TP53 CASP3 CASP8 CASP9 |

| | | | | | |
|---|---|---|---|---|---|
| | | | | | PPM1D CCND2 CCNE1 CCNG1 EI24 TP53I3 |
| 1.31E-07 | 39 | 136 | 2.733943015 | Path:hsa04210 Apoptosis | BCL2L11 CHUK PARP4 CTSD CTSV CTSZ GADD45A EIF2S1 ENDOG AKT1 FOS BBC3 APAF1 XIAP FAS FASLG IL3 JUN KRAS LMNB1 MCL1 ATM NFKB1 PIK3R1 PMAIP1 PIDD1 DIABLO BCL2 ACTB BID ACTG1 TP53 TRAF1 CASP3 CASP7 CASP8 CASP9 TNFSF10 AIFM1 |
| 1.04E-05 | 34 | 131 | 2.474408397 | Path:hsa04068 FoxO signaling pathway | BCL2L11 CDK2 CDKN1B CDKN2D GABARAP CHUK GADD45A EP300 AKT1 FOXG1 FOXO1 FOXO3 SIRT1 IGF1 IGF1R FASLG IL7R INSR KRAS SMAD3 MDM2 ATM PCK1 NLK PIK3R1 PRKAA1 CCND1 SOD2 STAT3 TGFBR1 TGFBR2 IRS4 TNFSF10 CCND2 |
| 0.000232929 | 24 | 93 | 2.460322581 | Path:hsa04657 IL-17 signaling pathway | CHUK FOS GSK3B HSP90AA1 S100A7A IFNG CXCL8 JUN MMP1 NFKB1 IL17RB MAPK6 PTGS2 S100A7 CCL2 CCL7 CXCL6 TNFAIP3 HSP90B1 TRAF3 TRAF5 CASP3 CASP8 IL17RC |
| 1.05E-07 | 53 | 214 | 2.361162383 | Path:hsa05417 Lipid and atherosclerosis | TLR6 VAV3 TAB1 CHUK ABCA1 EIF2S1 AKT1 FOS POU2F3 GSK3B APAF1 HSPA4 HSPA8 HSP90AA1 HSPD1 ICAM1 FAS FASLG CXCL8 IL12A IRAK1 JUN KRAS LDLR LYN MMP1 NFE2L2 NFKB1 PIK3R1 PLCB2 PLCB4 POU2F1 PPP3R1 RAP1B BCL2 CCL2 BID MAP2K4 SOD2 STAT3 TLR4 TP53 HSP90B1 TRAF3 VAV1 VCAM1 CASP3 CASP7 CASP8 CASP9 TNFSF10 EIF2AK3 CD36 |
| 2.54E-05 | 34 | 138 | 2.348894928 | Path:hsa05418 Fluid shear stress and atherosclerosis | CDH5 CHUK DUSP1 AKT1 FOS GSTA1 GSTP1 HMOX1 HSP90AA1 ICAM1 IFNG IL1A JUN MEF2C MGST3 NFE2L2 NFKB1 PDGFB PIK3R1 PRKAA1 BCL2 ACTB CCL2 MAP2K4 BMPR1A BMPR2 ACTG1 |

| | | | | | |
|---|---|---|---|---|---|
| | | | | | TP53 HSP90B1 TXN VCAM1 IL1R2 CAV1 CAV2 |
| 0.026510202 | 10 | 41 | 2.325304878 | Path:hsa04216 Ferroptosis | ACSL3 SLC7A11 FTH1 GPX4 HMOX1 MAP1LC3C PCBP2 SLC3A2 TFRC TP53 |
| 0.000448838 | 26 | 109 | 2.274105505 | Path:hsa04066 HIF-1 signaling pathway | CDKN1B EGLN3 ENO1 EP300 AKT1 HMOX1 IFNG IGF1 IGF1R IL6R INSR LDHB NFKB1 PDHA2 PDK1 PFKFB3 PFKP PIK3R1 BCL2 RPS6KB2 STAT3 ELOB TFRC TIMP1 TLR4 MKNK1 |
| 0.001399866 | 22 | 93 | 2.255295699 | Path:hsa04350 TGF-beta signaling pathway | EP300 ID1 ID4 IFNG SMAD1 SMAD3 SMAD5 SMAD6 SMAD7 MYC PPP2R1A RPS6KB2 GREM2 BMP2 BMP8B BMPR1A BMPR2 TGFBR1 TGFBR2 ACVR1B ZFYVE9 ZFYVE16 |
| 0.000692674 | 26 | 112 | 2.213191964 | Path:hsa04668 TNF signaling pathway | TAB1 CHUK JAG1 AKT1 VEGFD FOS ICAM1 FAS IRF1 JUN LIF NFKB1 PIK3R1 PTGS2 BCL3 CCL2 CXCL6 MAP2K4 TNFAIP3 TRAF1 TRAF3 TRAF5 VCAM1 CASP3 CASP7 CASP8 |

Table S18. Pathway enrichment. Figure 7E detailed data.

| Enrichment FDR | nGenes | Pathway Genes | Fold Enrichment | Pathway | Genes |
|---|---|---|---|---|---|
| 0.000162565 | 10 | 20 | 4.766875 | Vascular Smooth Muscle Cell/Pericyte Differentiation and Proliferation | SMAD3 SMAD5 TAGLN SMAD1 SRF FN1 GATA6 TGFBR1 TGFBR2 ID1 |
| 0.000827138 | 10 | 24 | 3.972395833 | Antiphospholipid Antibodies in Endothelial Cell | MAP2K4 CXCL8 JUN FOS VCAM1 F3 ICAM1 IL1A IRAK1 TLR4 |
| 0.000139705 | 13 | 32 | 3.873085938 | ROS in Triggering Vascular Inflammation | VCAM1 F3 AGT VAV1 FAS IFNG AGTR1 CXCL8 RASGRF1 FASLG ICAM1 PTGS2 CCL2 |
| 0.028233056 | 5 | 13 | 3.666826923 | IL1R STAT3 Signaling | MAP2K4 PRKCD STAT3 IL1A IRAK1 |
| 1.31E-07 | 28 | 77 | 3.466818182 | Apoptosis | ENDOG APAF1 CRADD FAS BCL2 GAS2 ATM TP53 APP DIABLO BBC3 CASP8 BCL2L11 CASP9 CASP7 CASP4 CHEK2 AKT1 CASP3 |

| | | | | | |
|---|---|---|---|---|---|
| | | | | | MCL1 TNFSF15 XIAP MDM2 FAF1 FASLG AIFM1 TNFSF10 BID |
| 0.006658644 | 8 | 22 | 3.466818182 | Low-Density Lipoproteins and Chemokines in Atherosclerosis | ITGB2 VCAM1 ICAM1 CCL2 CD36 TLR6 TLR4 LDLR |
| 0.001635157 | 11 | 31 | 3.382943548 | ER Stress (Unfolded Protein Response) | HSP90AA1 APAF1 EIF2AK3 PPP2R1A EIF2S1 BCL2 PPP1CC CASP9 CASP7 AKT1 CASP3 |
| 0.034649758 | 6 | 19 | 3.010657895 | Leptin in Insulin Synthesis and Secretion | INSR STAT3 IGF1 IGF1R IAPP AKT1 |
| 0.034649758 | 6 | 19 | 3.010657895 | Exocytosis Vesicle Docking | RAB3A STXBP3 RAB27A RHOQ RAB8A STX1A |
| 0.002920022 | 13 | 44 | 2.816789773 | Arterial Hypertension | NCOA3 JUN CAV1 FOS ESR1 PIK3R1 KRAS CXCL8 PTGS2 CCND1 EP300 AKT1 CCL2 |

Table S19. Spearman's correlation. Figure 2 detailed data.

| | miR-15a-5p | miR-16-5p | miR-17-5p | miR-20a-5p | miR-92a-3p | miR-93-5p | miR-153-3p | miR-185-5p | miR-186-5p | miR-210-3p | miR-222-3p | miR-424-5p | miR-486-5p |
|---|---|---|---|---|---|---|---|---|---|---|---|---|---|
| Sex (0-M, 1=F) | -0,17 | -0,13 | -0,29 | -0,21 | -0,07 | -0,13 | 0,05 | 0,04 | -0,15 | -0,21 | 0,06 | -0,08 | -0,09 |
| Diabetes Melitus | 0,01 | 0,01 | 0,10 | 0,17 | 0,04 | 0,09 | 0,23 | -0,19 | 0,15 | -0,15 | 0,18 | 0 | -0,08 |
| Dyslipidemia | 0,26 | 0,28 | 0,18 | 0,28 | 0,29 | 0,30 | 0,17 | 0,27 | 0,18 | 0,31 | 0,21 | 0,38 | 0,36 |
| Atrial Fibrillation | -0,22 | -0,19 | -0,18 | -0,12 | -0,34 | -0,22 | -0,03 | -0,11 | -0,11 | -0,14 | 0 | -0,12 | -0,25 |

Table S20. Spearman's correlation. Figure 4 detailed data.

| | miR-17-5p | miR-20a-5p | miR-23a-3p | miR-26b-5p | miR-181c-5p | miR-186-5p | miR-222-3p |
|---|---|---|---|---|---|---|---|
| sex (0-M, 1=F) | -0,07 | -0,1 | -0,19 | -0,19 | -0,12 | -0,09 | -0,1 |
| Diabetes Melitus | -0,04 | -0,04 | 0,07 | 0,1 | 0,05 | -0,04 | 0,16 |
| Dyslipidemia | 0,08 | 0,06 | 0,02 | 0,09 | 0,09 | 0,01 | -0,01 |
| Atrial Fibrillation | 0,04 | -0,02 | 0,04 | -0,03 | 0,1 | -0,02 | 0,11 |

**Complete Abbreviation List**

| | |
|---|---|
| ABHD2 | α/β Hydrolase Domain-Containing Protein 2 |
| ABL2 | Abelson Tyrosine-Protein Kinase 2 |
| ACTR2 | Actin-Related Protein 2 |
| AGO4 | Argonaute RISC Component 4 |
| Ang II | Angiotensin II |
| APP | Amyloid Precursor Protein |
| ARHGAP12 | Rho GTPase-Activating Protein 12 |
| ASA | Aspirin |
| ASPECTS | Alberta Stroke Program Early CT Score |
| ATG14 | Autophagy-Related Protein 14 |

| | |
|---|---|
| ATXN7L3B | Ataxin-7 Like Protein 3B |
| BDKRB ½ | Bradykinin Receptor B1/B2 |
| BDNF | Brain-Derived Neurotrophic Factor |
| BTG2 | BTG Anti-Proliferation Factor 2 |
| ABHD2 | α/β Hydrolase Domain-Containing Protein 2 |
| BTN3A3 | Butyrophilin Subfamily 3 Member A3 |
| BZW1 | Basic Leucine Zipper and W2 Domains 1 |
| CAPZA2 | Capping Actin Protein of Muscle Z-Line Subunit Alpha 2 |
| CCDC88C | Coiled-Coil Domain Containing 88C |
| CCND1 | Cyclin D1 |
| CCND2 | Cyclin D2 |
| CHIC1 | Cysteine-Rich Hydrophobic Domain-Containing Protein 1 |
| CLIP4 | CAP-Gly Domain Containing Linker Protein Family Member 4 |
| CNKSR3 | Connector Enhancer of Kinase Suppressor of Ras 3 |
| CNOT4 | CCR4-NOT Transcription Complex Subunit 4 |
| CRIM1 | Cysteine-Rich Motor Neuron 1 |
| CRK | CT10 Regulator of Kinase |
| CSNK1A1 | Casein Kinase 1 Alpha 1 |
| DCBLD2 | Discoidin, CUB and LCCL Domain Containing 2 |
| DCTN5 | Dynactin Subunit 5 |
| DIRECT | SAFE Direct Endovascular Treatment Versus Standard Bridging Therapy in Large Artery Anterior Circulation Stroke |
| DNAJC10 | DnaJ Heat Shock Protein Family Member C10 |
| EIF2B2 | Eukaryotic Translation Initiation Factor 2B Subunit Beta |
| EIF4G2 | Eukaryotic Translation Initiation Factor 4 Gamma 2 |
| ELK4 | ETS Transcription Factor ELK4 |
| FAS | Fas Cell Surface Death Receptor |
| FOXK1 | Forkhead Box Protein K1 |
| FoxO | Forkhead Box O |
| FZD9 | Frizzled Class Receptor 9 |
| HIF | Hypoxia-Inducible Factor |
| HIF-1 | Hypoxia-Inducible Factor 1 |
| HSP70 | Heat Shock Protein 70 |
| HSPA4L | Heat Shock Protein Family A (Hsp70) Member 4 Like |
| HSPA8 | Heat Shock Protein Family A (Hsp70) Member 8 |
| IGF | Insulin-like Growth Factor |
| IL-1 | Interleukin 1 |

| | |
|---|---|
| IL-5 | Interleukin 5 |
| IL-7 | Interleukin 7 |
| IL-10 | Interleukin 10 |
| IL-17 | Interleukin 17 |
| IL1R | Interleukin 1 Receptor |
| ITGA2 | Integrin Subunit Alpha 2 |
| KIF23 | Kinesin Family Member 23 |
| KLHL15 | Kelch Like Family Member 15 |
| L2HGDH | L-2-Hydroxyglutarate Dehydrogenase |
| LAMC1 | Laminin Subunit Gamma 1 |
| LDL | Low-Density Lipoproteins |
| MAP2K3 | Mitogen-Activated Protein Kinase 3 |
| MCL1 | Myeloid Cell Leukemia 1 |
| MINK1 | Misshapen-Like Kinase 1 |
| MTMR3 | Myotubularin-Related Protein 3 |
| mTORC1 | Mechanistic Target of Rapamycin Complex 1 |
| N4BP1 | NEDD4 Binding Protein 1 |
| NBIA | Neurodegeneration with Brain Iron Accumulation |
| NFκB | Nuclear Factor Kappa B |
| NO | Nitric Oxide |
| NUFIP1 | Nucleo-Cytoplasmic FMR1 Interacting Protein 1 |
| NUFIP2 | Nuclear FMRP Interacting Protein 2 |
| OCRL | Inositol Polyphosphate-5-Phosphatase |
| p53 | Tumor Protein p53 |
| PANK3 | Pantothenate Kinase 3 |
| PBMC | Peripheral Blood Mononuclear Cells |
| PDGF | Platelet-Derived Growth Factor |
| PDGFR-β | Platelet-Derived Growth Factor Receptor Beta |
| PHLPP2 | PH Domain And Leucine-Rich Repeat Protein Phosphatase 2 |
| PI3 kinase | Phosphoinositide 3-Kinase |
| PLRG1 | Pleiotropic Regulator 1 |
| PLS1 | Plastin 1 |
| PMAIP1 | Phorbol-12-Myristate-13-Acetate-Induced Protein 1 |
| PPP1R15B | Protein Phosphatase 1 Regulatory Subunit 15B |
| PPP6R3 | Protein Phosphatase 6 Regulatory Subunit 3 |
| PTP4A1 | Protein Tyrosine Phosphatase Type IVA, Member 1 |
| RAB3IP | RAB3A Interacting Protein |

| | |
|---|---|
| RACGAP1 | Rac GTPase-Activating Protein 1 |
| RBM12B | RNA Binding Motif Protein 12B |
| RFK | Riboflavin Kinase |
| RNASEH1 | Ribonuclease H1 |
| ROS | Reactive Oxygen Species |
| RPL14 | Ribosomal Protein L14 |
| RPRD2 | Regulation Of Nuclear Pre-MRNA Domain Containing 2 |
| SHOC2 | Leucine-Rich Repeat Protein SHOC2 |
| SIK1 | Salt-Inducible Kinase 1 |
| SLC28A1 | Solute Carrier Family 28 Member 1 |
| SMAD7 | SMAD Family Member 7 |
| SNTB2 | Syntrophin Beta 2 |
| SOCS5 | Suppressor Of Cytokine Signaling 5 |
| SPRED1 | Sprouty-Related, EVH1 Domain-Containing Protein 1 |
| SREK1IP1 | SREK1 Interaction Protein 1 |
| SSRP1 | Structure Specific Recognition Protein 1 |
| STAT3 | Signal Transducer and Activator of Transcription 3 |
| SWIFT | Solitaire With the Intention for Thrombectomy as Primary Endovascular Treatment |
| TGF-beta | Transforming Growth Factor Beta |
| TMEM100 | Transmembrane Protein 100 |
| TMEM245 | Transmembrane Protein 245 |
| TNF$\alpha$ | Tumor Necrosis Factor Alpha |
| TNRC6B | Trinucleotide Repeat Containing 6B |
| TXNIP | Thioredoxin Interacting Protein |
| USP48 | Ubiquitin-Specific Peptidase 48 |
| VEGF | Vascular Endothelial Growth Factor |
| VEGFA | Vascular Endothelial Growth Factor A |
| VEGFR2 | Vascular Endothelial Growth Factor Receptor 2 |
| VPS13C | Vacuolar Protein Sorting 13 Homolog C |
| WEE1 | WEE1 G2 Checkpoint Kinase |
| WNK3 | WNK Lysine Deficient Protein Kinase 3 |
| YTHDC1 | YTH Domain Containing 1 |
| ZMAT3 | Zinc Finger Matrin-Type 3 |
| ZNF426 | Zinc Finger Protein 426 |
| ZNF652 | |

Supplementary references

120. Exploring Common Distance Measures for Machine Learning and Data Science: A Comparative Analysis | by Sahel Eskandar | Medium [Internet]. [cited 2025 Feb 22]. Available from: https://medium.com/@eskandar.sahel/exploring-common-distance-measures-for-machine-learning-and-data-science-a-comparative-analysis-ea0216c93ba3
